# Development and Benchmarking of Open Force Field 2.0.0: The Sage Small Molecule Force Field

**DOI:** 10.1021/acs.jctc.3c00039

**Published:** 2023-05-11

**Authors:** Simon Boothroyd, Pavan Kumar Behara, Owen C. Madin, David F. Hahn, Hyesu Jang, Vytautas Gapsys, Jeffrey R. Wagner, Joshua T. Horton, David L. Dotson, Matthew W. Thompson, Jessica Maat, Trevor Gokey, Lee-Ping Wang, Daniel J. Cole, Michael K. Gilson, John D. Chodera, Christopher I. Bayly, Michael R. Shirts, David L. Mobley

**Affiliations:** † Boothroyd Scientific Consulting Ltd., London WC2H 9JQ, U.K.; ‡ Department of Pharmaceutical Sciences, University of California, Irvine, California 92697, United States; ¶ Chemical & Biological Engineering Department, University of Colorado Boulder, Boulder, Colorado 80309, United States; § Computational Chemistry, Janssen Research & Development, Turnhoutseweg 30, Beerse B-2340, Belgium; ∥ Chemistry Department, The University of California at Davis, Davis, California 95616, United States; ⊥ OpenEye Scientific Software, Santa Fe, New Mexico 87508, United States; # Computational Biomolecular Dynamics Group, Department of Theoretical and Computational Biophysics, Max Planck Institute for Multidisciplinary Sciences, Am Fassberg 11, D-37077, Göttingen, Germany; @ The Open Force Field Initiative, Open Molecular Software Foundation, Davis, California 95616, United States; △ School of Natural and Environmental Sciences, Newcastle University, Newcastle upon Tyne NE1 7RU, U.K.; ∇ Datryllic LLC, Phoenix, Arizona 85003, United States; ◇ Department of Chemistry, University of California, Irvine, California 92697, United States; ○ Skaggs School of Pharmacy and Pharmaceutical Sciences, The University of California at San Diego, La Jolla, California 92093, United States; ◆ Computational & Systems Biology Program, Sloan Kettering Institute, Memorial Sloan Kettering Cancer Center, New York, New York 10065, United States

## Abstract

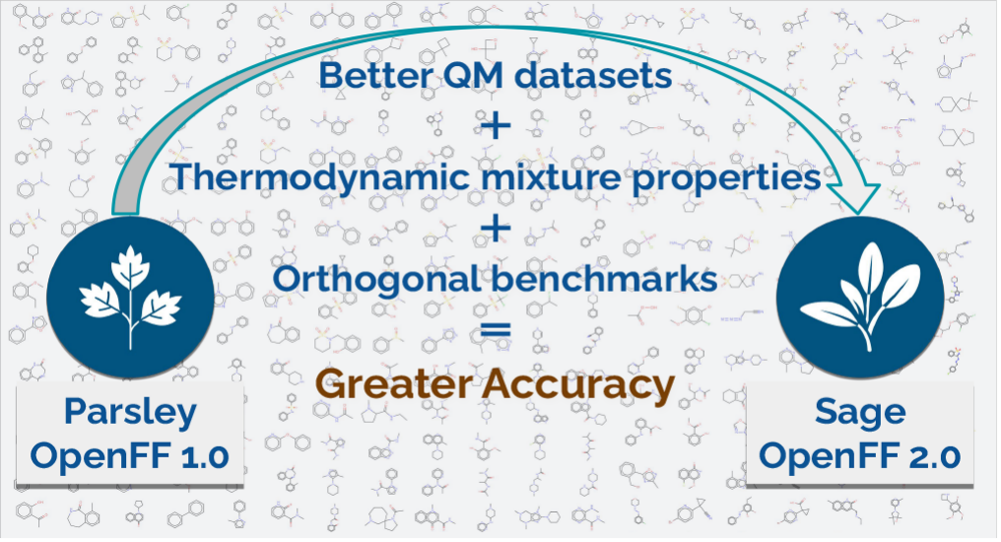

We introduce the Open Force Field (OpenFF) 2.0.0 small molecule force field for drug-like molecules, code-named Sage, which builds upon our previous iteration, Parsley. OpenFF force fields are based on direct chemical perception, which generalizes easily to highly diverse sets of chemistries based on substructure queries. Like the previous OpenFF iterations, the Sage generation of OpenFF force fields was validated in protein–ligand simulations to be compatible with AMBER biopolymer force fields. In this work, we detail the methodology used to develop this force field, as well as the innovations and improvements introduced since the release of Parsley 1.0.0. One particularly significant feature of Sage is a set of improved Lennard-Jones (LJ) parameters retrained against condensed phase mixture data, the first refit of LJ parameters in the OpenFF small molecule force field line. Sage also includes valence parameters refit to a larger database of quantum chemical calculations than previous versions, as well as improvements in how this fitting is performed. Force field benchmarks show improvements in general metrics of performance against quantum chemistry reference data such as root-mean-square deviations (RMSD) of optimized conformer geometries, torsion fingerprint deviations (TFD), and improved relative conformer energetics (ΔΔ*E*). We present a variety of benchmarks for these metrics against our previous force fields as well as in some cases other small molecule force fields. Sage also demonstrates improved performance in estimating physical properties, including comparison against experimental data from various thermodynamic databases for small molecule properties such as Δ*H*_mix_, ρ(*x*), Δ*G*_solv_, and Δ*G*_trans_. Additionally, we benchmarked against protein–ligand binding free energies (Δ*G*_bind_), where Sage yields results statistically similar to previous force fields. All the data is made publicly available along with complete details on how to reproduce the training results at https://github.com/openforcefield/openff-sage.

## 1 Introduction

Atomistic force fields describe the potential energy surface of molecular systems as a function of atomic positions. Force fields, while often relatively simple in functional form, have been widely adopted in computational chemistry and biophysics due to their balance between chemical accuracy and computational efficiency.^1−13^

Molecular dynamics simulations performed with force fields have been used to study the mechanisms of many biological phenomena, including protein folding,^14−16^ membrane transport,^17,18^ identification of active sites,^19,20^ docking of ligands,^21^ and protein–ligand binding.^22−24^ Protein–ligand interactions are of particular interest to the pharmaceutical industry, as such methods can accelerate drug discovery by identifying promising candidates *in silico*.^25,26^ This process, known as computer-aided drug design (CADD), requires quantitatively accurate descriptions of many chemically diverse drug candidates and their interactions with different chemical moieties and accurate predictions of their physical properties in various environments. In order to model the protein–ligand complex, we need accurate force fields for both the specific protein chemistries (many of which have been proposed, validated, and tested^8,11,12,27−34^) and the small molecule ligands.^3,10,13^ As the space of potential drug-like molecules is chemically complex and combinatorially large,^35,36^ a small molecule force field should be able to model a diverse set of molecules with high accuracy. OpenFF Sage 2.0.0 achieves these goals by combining the generality of SMIRNOFF direct chemical perception with extensive parameter refitting to improve accuracy.

### 1.1 OpenFF Innovations: Journey from Parsley to Sage

OpenFF Parsley 1.0.0, the first version of our Parsley generation of small molecule force fields^10^ achieved excellent coverage of chemical space with a novel direct chemical perception scheme^37^ and similar accuracy to other small molecule force fields, as measured on protein–ligand binding free energies.^10,38^ In contrast to indirect chemical perception, or atom typing, direct chemical perception involves substructure based parameter assignment that brings together complex chemistries under one physically intuitive chemical grouping. SMIRKS patterns^39^ are used to define these groups, and the associated parameters can be applied to any substructure match in any molecule, thus making it more general. Rather than having the chemical environment around a parameter being encoded in the atom types, the chemical environment is used to directly assign the parameters via these SMIRKS patterns. This direct chemical perception scheme greatly reduces the number of empirical force field parameters, facilitating rapid refit of parameters to improve chemical accuracy. As atomic partial charges are due to more global effects of chemical environment than SMIRKS strings can provide, another key ingredient of OpenFF force fields is the use of the AM1-BCC model for partial charge assignment, a fast and very widely used atomic charge model for organic molecules.^40,41^

Our underlying philosophy throughout the OpenFF effort is to combine modern force field optimization techniques and data set selection pipelines to rapidly produce and update new small molecule force fields. A previous paper^37^ outlined the concept of parameter-type based force fields using the SMIRNOFF format. In Parsley 1.0.0, most of the initial set of nonbonded parameters was ported from parm@Frosst^7^ into what we called SMIRNOFF99Frosst, an informal AMBER family small molecule force field. SMIRNOFF99Frosst was used as a starting point for Parsley with significant optimization of the valence parameters through fits with an extensive set of QM calculations.^10^ Since both GAFF and Parsley share roots in the AMBER family of force fields, this meant that their nonbonded parameters were virtually identical.^10,42^

Since the release of Parsley, we have made several updates to our force field, consisting of improved valence parameters as well as bug fixes. Parsley 1.1.0 included the addition of new nitrogen-centered improper torsion terms to better describe the planar and pyramidal structures that are often difficult to differentiate.^43,44^ This was followed by Parsley 1.2.0^45^ which included a major redesign of the quantum chemical training data sets and a full valence parameter refit to this new data set. This training data curation^46^ resulted in significant improvement in relative conformer energies, optimized geometries, and torsional profiles with respect to accurate high-level *ab initio* data when compared to Parsley 1.0.0. Revisions after Parsley 1.2.0 include new torsion parameters for dialkyl amides in Parsley 1.3.0 to improve amide torsional energy profiles;^47^ in Parsley 1.3.1, a minor regression in the accuracy of the description of sulfonamides was corrected.^48^

Building on the foundation of Open Force Field Parsley generation of force fields, we now introduce the OpenFF Sage 2.0.0 small molecule force field which extends our previous work by continued refining of valence terms and, for the first time, refitting the Lennard-Jones (LJ) parameters. Like Parsley, Sage is applicable to drug-like molecules covering the chemical space C, H, O, N, P, S, F, Cl, Br, and I, and the monoatomic ions Li^+^, Na^+^, K^+^, Rb^+^, F^–^, Cl^–^, Br^–^, and I^–^. Sage again included substantial work retraining the valence parameters used in Parsley, but the most significant update to Sage is the retraining of select Lennard-Jones (LJ) parameters to physical properties. LJ parameters in previous generation Open Force Fields were taken from AMBER parm99^49^ and parm@Frosst^7^ force fields. The LJ parameters in Sage 2.0.0 were optimized against condensed phase physical properties, including enthalpy of mixing and densities measured for both pure and binary mixtures. The inclusion of such properties measured for mixtures has been shown to be critical to accurately capture interactions of unlike chemistries.^50^ Physical properties of aqueous systems using TIP3P water^51^ were directly included in the training set ensuring maximal self-consistency between the small molecule and water interactions. TIP3P was chosen as the water model, as it has typically been considered the default for AMBER-style force fields. Sage is most similar (though not equivalent to) an AMBER force field because of its roots in the AMBER-variant parm@Frosst, and AMBER force fields for proteins and nucleic acids are tested and recommended with OpenFF small molecule force fields. Thus, it seemed preferable to use TIP3P at least until we are able to refit a fully consistent biopolymer and small molecule force field for compatibility with an alternate optimized water model.

### 1.2 Sage Training Data and Methods

We give full details of our training and fitting procedures in the Methods (section 2), below, but here we provide a brief overview of the key training and test data used to produce the Sage force field.

#### 1.2.1 Overall Optimization Strategy

**Figure 1:**
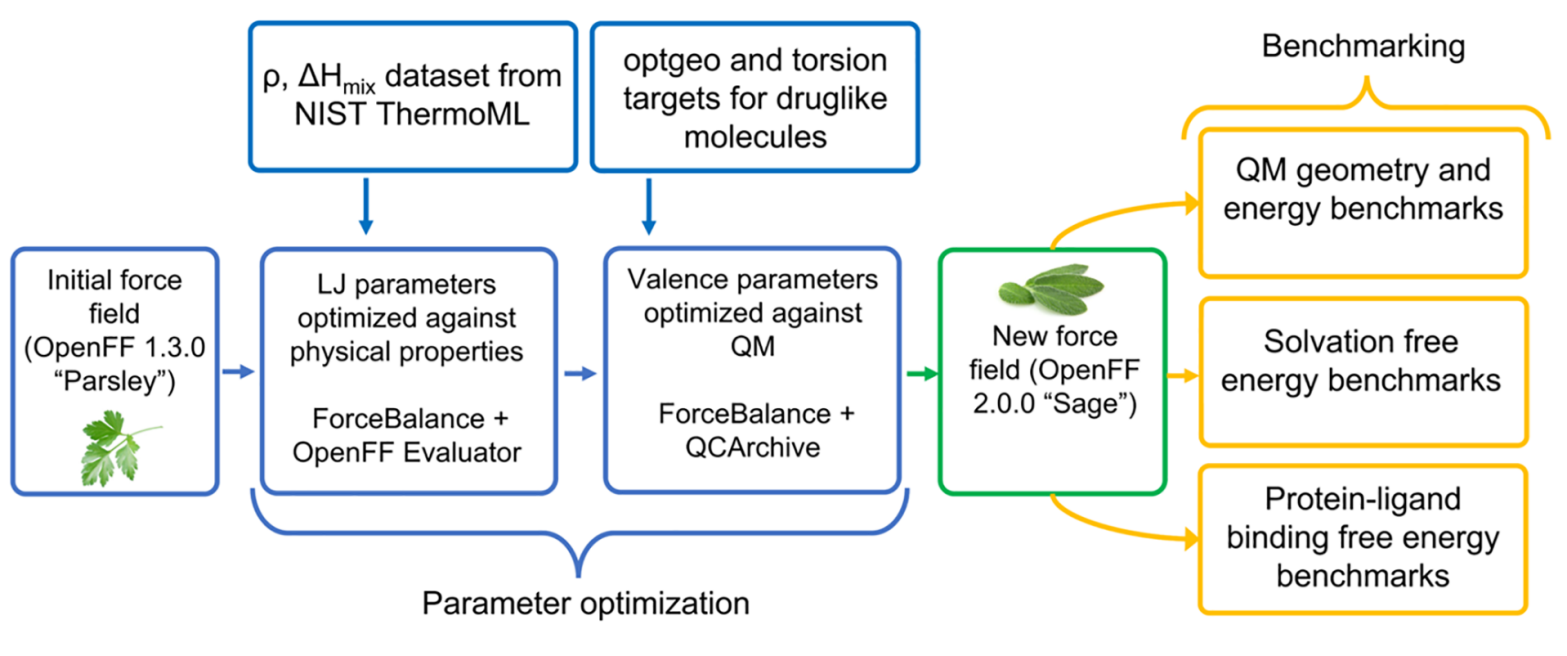
Sage 2.0.0 fitting pipeline is composed of three stages. Beginning with an initial force field (Parsley 1.3.0), in the first step, selected LJ parameters were refit against liquid densities and enthalpies of mixing sourced from the NIST ThermoML archive, producing an intermediate force field (*vdw-v1*). In the second step, valence parameters from *vdw-v1* were trained against optimized geometries and torsion energy profiles of drug-like molecules, resulting in a new force field (Sage 2.0.0) trained against curated quantum chemical and condensed-phase property data. Both steps optimize force field parameters using a regularized least-squares approach implemented in ForceBalance. Sage 2.0.0 was then benchmarked against test sets of gas phase QM equilibrium structures, solvation free energies, and protein–ligand binding free energies.

Force field optimization and validation were performed in three stages (workflow shown in Figure 1):

1. Training of selected LJ parameters against experimental measurements of physical properties (densities and enthalpies of mixing) using Parsley 1.3.0 valence parameters.
2. Starting from a force field with the refitted LJ parameters (*vdw-v1*), training valence parameters with QM data with fixed LJ parameters.
3. Validation of the new force field using a variety of quantum mechanical, physical property, and protein–ligand binding data.

The starting point for this refit is Parsley 1.3.0, which includes valence parameters fit against improved training data (compared to Parsley 1.0.0) and several new bond, angle, and torsion types that address specific chemistries. The reversion of the sulfonamide angle in Parsley 1.3.1 was not included in this refit, as development on Sage 2.0.0 was already underway by the time Parsley 1.3.1 was released. However, testing the parameters from Sage on the reported discrepancy, in “O∼S∼N” angles of sulfonamides, showed that this problem was resolved by the Sage workflow and did not require further fixes.

#### 1.2.2 LJ Training Data

A large subset of the condensed phase physical property measurements of binary mixtures from the NIST ThermoML Archive^52^ was used to refit the LJ parameters. Our choice of mixture data was motivated by a previous pilot study^50^ that demonstrated advantages to training LJ parameters against condensed phase mixtures as opposed to pure liquid properties. Mixture data readily captures interactions between unlike molecules, allows for the selection of data at multiple concentrations, and is more readily available in modern, validated databases such as the NIST ThermoML Archive. The pilot study demonstrated that LJ parameters refitted against mixture densities and enthalpies of mixing better capture interactions between both like and unlike molecules than those refitted against pure liquid densities and enthalpies of vaporization. Given that Sage marks the first time the LJ parameters of an OpenFF force field were refit, and especially as they were refit to properties of mixtures for the first time, the training set was selected to encompass as broad a chemical space for which there was data, rather than targeting specific groups. Identifying problematic groups will likely be the focus of future studies once the performance and deficiencies of the refit parameters are better understood. As the amount of experimental data is rather limited, the number of functional groups covered by the LJ training set was fewer than that by the valence training set. For the full refit of the Sage force field, a total of 30 LJ parameters (*R*_min/2_ and ϵ for 15 LJ types) representing carbon, hydrogen, oxygen, nitrogen, chlorine, and bromine environments were included.

#### 1.2.3 Valence Parameter Training Data

Quantum mechanical (QM) data in the form of optimized conformer geometries and one-dimensional torsional profiles were used to train valence parameters, outlined in Table 1. 3663 optimized conformer geometries and 713 one-dimensional torsion scans from Generation 2 data sets were used for training. Out of 302 valence parameters (angles, bonds, and proper torsions), 184 parameters were refitted. In Sage, two main changes in the choice of valence parameter training data were (1) balancing the contributions of conformers from each molecule and (2) removing vibrational frequencies as training targets. We pruned the number of conformers in optimized geometry targets in order to balance contributions to the objective function. This balancing was necessary as some training set molecules have >50 conformers while others have <10, introducing a large bias toward molecules with many minima. We also removed vibrational frequencies as training targets, as we found that misalignment of vibrational modes degraded performance and in some cases led to pathologies in the parameters, such as the problems with sulfonamide in Parsley 1.3.0 described above.

**Table 1:** Generation 2 Optimization and TorsionDrive Data Sets, Listed Here, Were Used in Training Valence Parameters of Sage^a^

TorsionDrive data sets
OpenFF Gen 2 Torsion Set 1 Roche 2 (122 1D scans)
OpenFF Gen 2 Torsion Set 2 Coverage 2 (117 1D scans)
OpenFF Gen 2 Torsion Set 3 Pfizer Discrepancy 2 (69 1D scans)
OpenFF Gen 2 Torsion Set 4 eMolecules Discrepancy 2 (234 1D scans)
OpenFF Gen 2 Torsion Set 5 Bayer 2 (151 1D scans)
OpenFF Gen 2 Torsion Set 6 supplemental 2 (20 1D scans)

Optimization data sets
OpenFF Gen 2 Opt Set 1 Roche (278 conformers)
OpenFF Gen 2 Opt Set 2 Coverage (159 conformers)
OpenFF Gen 2 Opt Set 3 Pfizer Discrepancy (153 conformers)
OpenFF Gen 2 Opt Set 4 eMolecules Discrepancy (1407 conformers)
OpenFF Gen 2 Opt Set 5 Bayer (1666 conformers)

a From these data sets, around 3663 optimized geometries and 713 1D torsion scans were used in training, and the explicit number targets from each subset are enumerated in this table. The QCA ids of the records are available on the github repo, https://github.com/openforcefield/openff-sage/tree/2.0.0-rc.1/data-set-curation/quantum-chemical/data-sets. The json files 1-2-0-opt-set-v3.json and 1-2-0-td-set.json for *opt-geo* targets and *torsion-profile* targets, respectively, contain the QCA record information.

### 1.3 Benchmarking the Sage Force Field

After refitting, the new force field was benchmarked against several test sets designed to assess parameter quality and transferability. These data sets provide a holistic benchmark for the force field, including QM geometries for drug-like molecules to assess valence parameter quality, solvation and transfer free energies for small organic molecules to assess nonbonded parameter quality, and protein–ligand binding free energies to ensure the refit did not adversely affect performance on this critical measure. These benchmark data sets are sourced from high-quality public databases like FreeSolv,^53^ QCArchive,^54^ and the NIST ThermoML archive.^55−60^ The scripts to access the data and run the benchmarks are available at https://github.com/openforcefield/openff-sage/tree/2.0.0-rc.1/inputs-and-results/benchmarks. The release assets of the repository, https://github.com/openforcefield/openff-sage/releases, contain the QC benchmark structure files in SDF format (https://github.com/openforcefield/openff-sage/releases/download/2.0.0-rc.1/QM_Benchmarks_qc_opt_geo.tar.gz), as well as csv files with the reference and estimated property data for the vdW benchmark (https://github.com/openforcefield/openff-sage/releases/download/2.0.0-rc.1/sfe-results.tar.gz).

Unlike the training set, the test set did not include either enthalpy of mixing or density measurements. As the amount of such data was limited, a focus was given to building as diverse a training set as possible. We believe that the inclusion of related properties such as transfer free energies should yield similar insight however. We also opted not to include enthalpies of vaporization in the test set. In previous work,^50^ we showed that training to enthalpies of mixing and densities of binary mixtures does not in general degrade the performance of such properties. Further, due to the force field not containing polarizability terms, we do not expect to see significant improvements to the enthalpy of vaporization; indeed, a force field that gives better condensed-phase properties might yield worse enthalpies of vaporization.

## 2 Methods

### 2.1 Software and Data Infrastructure Used to Build Force Fields

The open source software stack that supports the development of our force fields includes several components. The most crucial of these are workflows for generation of QM data sets (including optimized geometries, torsion scans, and vibrational frequencies), a publicly accessible database with seamless data retrieval, a force field optimizer, and benchmarking infrastructure. The major software components that are used in building our force fields are

- ForceBalance: A versatile package for force field optimization^61−63^
- GeomeTRIC: Geometry optimizer for molecular structures with translation-rotation-internal coordinate (TRIC) system^64^
- Nonbonded: Automated workflow for the optimization and assessment of the nonbonded interaction parameters of force fields against physical property data sets^65^
- OpenFF-Toolkit: Chemistry toolkit for working with SMIRNOFF format force fields, as well as interface to various cheminformatics back-ends (RDKit^66^ and OpenEye^67^), and molecular dynamics engines (OpenMM)^68^
- OpenFF-QCSubmit: Data set building, validation, and data retrieval from QCArchive^69^
- OpenFF-Evaluator: Automated and highly scalable physical property evaluator^70^
- OpenFF-BespokeFit: Processing QCArchive data and creating ForceBalance inputs^69^
- OpenMM: High performance molecular dynamics package with a variety of enhanced sampling methods^71^
- PMX: Toolkit for free-energy calculation setup/analysis and biomolecular structure handling^72^
- Psi4: Highly parallel electronic structure code covering a large range of methods, density functional/basis set combinations, and property evaluations^73^
- QCEngine: A common Python interface to various Quantum Chemistry packages^74^
- QCFractal: Server for facilitating Quantum Chemistry calculations on large compute clusters and archiving the results in a database^54^
- QCArchive: Openly accessible QCFractal server of Quantum Chemistry calculations, operated by MolSSI^54^
- TorsionDrive: Highly efficient, wavefront propagation based torsion potential scanner^75^

### 2.2 Description of LJ Training

#### 2.2.1 Details of LJ Training Method

We refit a total of 30 Lennard-Jones parameters (LJ *R*_min/2_ and ϵ for 15 LJ interaction SMIRKS types); these types and the chemistries they describe are listed in Table 2. The parameters for another 20 LJ types (including 9 LJ types for ions) were left unchanged.

**Table 2:** All LJ SMIRKS Types Adjusted in the Training of Sage 2.0.0, along with Descriptions of the Chemical Contexts They Describe^a^

refit SMIRKS type	description
[#1:1]-[#6X4]	hydrogen attached to tetravalent carbon
[#1:1]-([#6X4])-[#7,#8,#9,#16,#17,#35]	hydrogen attached to tetravalent carbon attached to an electronegative atom
[#1:1]-[#6X3]	hydrogen attached to trivalent carbon
[#1:1]-[#6X3]∼[#7,#8,#9,#16,#17,#35]	hydrogen attached to trivalent carbon attached to an electronegative atom
[#1:1]-[#6X3](∼[#7,#8,#9,#16,#17,#35])∼[#7,#8,#9,#16,#17,#35]	hydrogen attached to trivalent carbon attached to two electronegative atoms
[#1:1]-[#7]	hydrogen attached to nitrogen
[#1:1]-[#8]	hydrogen attached to oxygen
[#6:1]	Generic carbon
[#6X4:1]	tetravalent carbon
[#8:1]	generic oxygen
[#8X2H0+0:1]	divalent oxygen with no hydrogens attached
[#8X2H1+0:1]	divalent oxygen with one hydrogen attached
[#7:1]	generic nitrogen
[#17:1]	generic chlorine
[#35:1]	generic bromine

a LJ ϵ and σ are adjusted for each of these types.

Our original goal was to refit all LJ SMIRKS types, but there were insufficiently diverse physical property training data (determined as having fewer than 5 data points for either density or enthalpy of mixing) for some chemistries. This refit covered most parameters for the chemical space of hydrogen, carbon, nitrogen, oxygen, chlorine, and bromine; notable types that were not refit describe fluorine, phosphorus, and sulfur. The training data, described in detail in section 2.2.2, consisted of measurements of densities of pure liquids ρ, of binary liquid mixtures ρ_mix_, and enthalpies of mixing of binary liquid mixtures Δ*H*_mix_. Optimization was performed by iteratively minimizing a ForceBalance objective function *L*_LJ_(θ), a weighted least-squares objective comparing simulation estimates of training data points with their experimental values, shown in eq 1.
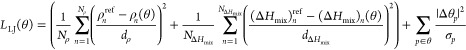
1In this equation, *N*_ρ_ and *N*_Δ*H*_mix__ represent the total number of measurements of ρ and Δ*H*_mix_ in the training set, respectively; θ represents the set of LJ parameters optimized and Δθ_*p*_ represents the change from the initial values taken from Parsley 1.3.0. Mixture and pure densities are pooled together in this objective function, so ρ here represents the set of all densities. The constants *d*_ρ_ and *d*_Δ*H*_mix__ represent scaling factors for those two data types and are set to *d*_ρ_ = 0.05 g/mL and *d*_Δ*H*_mix__ = 1.6 kJ/mol. These scaling factors represent the relative weight given to each data type and are set such that both ρ and Δ*H*_mix_ contribute roughly equally to the objective function for the initial Parsley 1.3.0 force field. The scale σ_*p*_ in the regularization term is set to 0.1 kcal/mol for all vdW ϵ and 1 Å for all vdW *R*_min/2_ and were chosen based on values that led to successful optimizations in our previous study.^50^

Each optimization iteration consists of:

1. Estimating all physical properties in the training data set by simulation and their gradients with respect to the LJ parameters being optimized, using the OpenFF Evaluator software package.^70^
2. Calculating the value of the objective function at the current parameter set.
3. Selecting a new set of parameter values using the L-BFGS-B algorithm.

This optimization is allowed to continue until the objective function is observed to fluctuate around a constant minimum.^50^ The minor fluctuations around the minimum in the objective function are expected due to noise in the gradients of the physical properties with respect to the LJ parameters caused by finite simulation lengths. In practice, it was found that 15 iterations was sufficient to consistently meet this criterion, which was completed in roughly 1 week using a pool of 60 GPUs.

All simulations used to estimate physical properties in the LJ training and test data sets are performed with the OpenFF Evaluator^70^ software package version 0.3.4,^76^ using the default simulation workflow schemas, described below. Where possible, simulation results are used to estimate multiple properties (e.g., using the same pure liquid simulations in both a ρ and Δ*H*_mix_ calculation). All liquid simulations used in the optimization are performed with 1000 molecule simulation boxes created with PackMOL.^77^ After energy minimization and a 0.2 ns equilibration run, each box is simulated for 2 ns in the NPT ensemble. Ensemble averages used in physical property calculations are taken from uncorrelated snapshots, subsampled with the method proposed by Chodera.^78^ Physical property calculations are calculated with the same procedures used in Boothroyd et al.^50^

#### 2.2.2 Details of LJ Training Data

All data used to train the LJ parameters is sourced from the NIST ThermoML Archive,^52^ a machine-readable collection of thermophysical property data maintained by NIST that draws from several scientific journals. The training data set consists of measurements of 70 neat liquid densities (ρ), 485 densities of binary mixtures (ρ_mix_), and 477 enthalpies of mixing of binary mixtures (Δ*H*_mix_). These measurements are selected at close-to-ambient conditions (99.9–101.4 kPa, 288.15–318.15 K) and are selected from molecules containing only hydrogen, carbon, nitrogen, oxygen, chlorine, and bromine. The measurements represent a diverse range of functional groups, chosen so that each included functional group includes at least 5 measurements. Long-chain alkanes and ethers were excluded due to difficulty packing simulation boxes and long correlation times in simulation, while 1,3-diketones were excluded due to their propensity for ketone–enol tautomerism. For physical properties of binary mixtures, we attempt to select 3 measurements at concentrations close to (*x*_1_ = 0.25, *x*_2_ = 0.75), (*x*_1_ = 0.5, *x*_2_ = 0.5), and (*x*_1_ = 0.75, *x*_2_ = 0.25). We enforced a minimum concentration of *x*_*i*_ = 0.05, where *x*_*i*_ is the mole fraction of either component, to avoid problems with sampling and convergence caused by a low absolute number of molecules of that component in a simulation box. Data in the ThermoML Archive includes expanded 95% CI uncertainty estimates provided by NIST, estimated either through uncertainty propagation or internal validation of methods/data consistency. While these uncertainty estimates are not directly used in the training process, they provide additional confidence in the data. This data set is available at https://github.com/openforcefield/openff-sage/tree/main/data-set-curation/physical-property/optimizations/data-sets.

### 2.3 Description of Valence Parameter Training

#### 2.3.1 Expansion of QM Training Data Sets

Valence parameters (angles, bonds, and proper torsions) in Sage were trained on QM data from a diverse range of data sets. We used three categories of QM data sets: optimized geometries of conformers, torsion scans of rotations around a specific central bond in molecules, and Hessian calculations on equilibrium geometries. The data sets can be broadly classified into two generations: the first generation data sets, whose main focus was full coverage of all force field parameters, and the second generation data sets, which improved chemical diversity. Training data sets used in one or more of the force fields discussed here, as referenced on MolSSI’s QCArchive, are listed in Table 3. Section 2.3.2 has details on the data used from these data sets in training a specific version of the force field, and Table 1 lists Sage-specific training data.

**Table 3:** List of QM Data Sets of Optimized Geometries and 1D Torsion Scans, Curated and Used for Training One or More of the Force Fields Discussed Here, as Referenced on MolSSI’s Publicly Accessible Repository QCArchive^a^

generation	TorsionDrive data set	optimization data set (each set has a corresponding basic data set)
Generation 1 training sets (<Parsley 1.2.0), 620 unique molecules	OpenFF Group 1 Torsions (820 1D scans)	OpenFF Optimization Set 1 (937 conformers)
	SMIRNOFF Coverage Torsion Set 1 (585 1D scans)	SMIRNOFF Coverage Set 1 (1132 conformers)
	OpenFF Group 1 Torsions 2 (19 1D scans)	
	OpenFF Group 1 Torsions 3 (6 1D scans)	
Generation 2 training sets (≥Parsley 1.2.0), 1526 unique molecules	OpenFF Gen 2 Torsion Set 1 Roche 2 (142 1D scans)	OpenFF Gen 2 Opt Set 1 Roche (298 conformers)
	OpenFF Gen 2 Torsion Set 2 Coverage 2 (157 1D scans)	OpenFF Gen 2 Opt Set 2 Coverage (373 conformers)
	OpenFF Gen 2 Torsion Set 3 Pfizer Discrepancy 2 (82 1D scans)	OpenFF Gen 2 Opt Set 3 Pfizer Discrepancy (197 conformers)
	OpenFF Gen 2 Torsion Set 4 eMolecules Discrepancy 2 (272 1D scans)	OpenFF Gen 2 Opt Set 4 eMolecules Discrepancy (2201 conformers)
	OpenFF Gen 2 Torsion Set 5 Bayer 2 (219 1D scans)	OpenFF Gen 2 Opt Set 5 Bayer (1850 conformers)
	OpenFF Gen 2 Torsion Set 6 supplemental 2 (22 1D scans)	

a As discussed in the text, Generation 1 data sets were the first set generated with coverage of all parameters as the main objective, whereas Generation 2 data sets were generated to increase the chemical diversity. Hessian data sets (termed as “basic data set”) for the equilibrium geometries of all the optimization data sets listed here are also available on QCArchive. Each of the Hessian data sets has the exact same data set name as the corresponding optimization data set but Hessians for the final optimized geometries. A complete list of OpenFF data sets, including those not used in fitting here, can be found at https://github.com/openforcefield/qca-dataset-submission#dude-wheres-my-dataset.

The second generation data sets were sourced from molecules of interest from our industry partners. A large compendium of molecules was curated using fingerprint-based clustering. MACCS keys fingerprints^79^ with a default path length of four bonds were generated for all, and a matrix of graph similarity scores (Tanimoto) was evaluated. For each of the bonded parameters, clustering with DBSCAN^80−82^ based on these graph similarity scores was done, with cluster sizes of at least 5 molecules, and representative molecules were picked randomly from each cluster, with the goal of ensuring that if multiple training set molecules used the same parameter, these molecules would be chemically diverse. The tautomeric and isomeric states were expanded for the filtered molecules using the CMILES and Fragmenter packages.^83^ The final list of molecules in the optimization data sets listed in Table 3 were generated following these steps.

Along with an increase in chemical diversity, additional large molecules (>20 heavy atoms) were included in Generation 2 sets compared to Generation 1. These larger molecules also included more flexible molecules with many rotatable bonds sampling a range of structurally diverse local minima, as well as manifesting complex nonbonded intramolecular interactions arising from diverse chemistries, thus better sampling from possible complexities in training torsional space. A comparison of number of heavy atoms between Generation 1 and 2 data sets is shown in SI Figure S3, and an extended tail in the region of >20 heavy atoms can be observed.

We tested the coverage of Generation 2 data sets of approximately 200 pharmaceutically relevant functional groups. In order to explore the functionality, we constructed a graph representation of the data set. Functional groups are represented as nodes, and edges were constructed between nodes if both the functional groups represented by the nodes were present in the same molecule. Higher level abstractions of chemical environments such as aromatic, heterocycle, and so forth, were not considered as a functional group to avoid clutter. From this analysis, the Generation 2 data set had 108 nodes which covers an additional 45 functional groups compared to Generation 1, which had 63 nodes. Generation 2 data set’s network of functional groups had 5533 edges, whereas Generation 1 had 739 edges. This increase in number of edges shows that a larger combinatorial mixing of functional groups was achieved with Generation 2 training data. The difference in connectivity between different clusters is shown in SI Figure S1, and the functional groups were tabulated in SI Table S1.6.

Torsion drive data sets were generated by enumerating all the torsions in molecules from Generation 2 optimization data sets and picking select molecules for a one-dimensional torsion scan. These torsions were chosen by listing all torsion definitions applied to each rotatable central bond in each molecule. This list of molecules was filtered, with each torsion scan ideally scanning a torsion exercising a single torsion in the force field. However, this was not always possible given the set of available molecules, so when no suitable molecules could be found, the number of allowed overlaps with other torsions was incremented by one and the process repeated until qualifying molecules were found.^46^ For the 1-dimensional torsional scans, the dihedral angle was sampled on a grid of 24 points spanning the range [−180, 180] with a spacing of 15°, and the torsion potential scans were performed using the TorsionDrive package.^75^ For some of the molecules with in-ring torsions only a subset of the 24 grid points were retained as a full rotation takes the system into unphysically high energy regions and distorts the torsion drive. This energy cutoff on the grid was 0.05 hartree (∼31.4 kcal/mol). TorsionDrive uses wavefront propagation to find the minimum energy conformation at each torsion angle along a torsion scan.^75^

The QM data sets described above were generated using the B3LYP-D3BJ/DZVP level of QM theory,^84−87^ the same level of theory used to generate training data for Parsley.^10^ The choice of QM theory level at which the training data is generated should be sufficiently accurate for metrics such as conformer energies and torsion profile energetics for a wide range of molecules. This choice of QM theory level includes the choice of functional as well as the basis set. Prior benchmarks using this theory level^88^ showed optimal performance on conformer energetics of the MPCONF196 data set,^89^ which contains small peptides and medium-sized macrocycles, and on the YMPJ data set,^90^ which is a data set of natural amino acids. We have also conducted our own work benchmarking levels of theory for this data and reached a similar conclusion, which will be reported in a separate study.

All the OpenFF-generated QM data sets reside on MolSSI’s QCArchive public data repository and are accessible via its Python API (QCPortal) or by using OpenFF-QCSubmit.^69^ The Sage release github repository (https://github.com/openforcefield/openff-sage) has Python scripts for data set download and processing the downloaded records, using the OpenFF-QCSubmit package.

#### 2.3.2 QM Training Data Used in Training Valence Parameters

Training for the Sage 2.0.0 release built on training data sets for the 1.2.0 and 1.3.0 data sets, which have not been previously reported in detail, so these are briefly described here.

In training Parsley 1.2.0, 4745 optimized geometries, 710 1D torsion scans, and 1189 Hessians (for vibrational frequencies targets) from Generation 2 data sets were used. The explicit target files used in training Parsley 1.2.0 and the ForceBalance output can be found in the release tarball for the 1.2.0 force field.^91^

For training Parsley 1.3.0, which was a minor release to correct discrepancies in amide torsional profiles, a mix of data from both generations was used. Molecules for torsion profile targets were picked from Generation 1 data sets due to lack of molecules in Generation 2 data sets that have a planar amide bond along with planar geometries. The presence of mostly nonplanar molecules with amides in Generation 2 data sets was due to strong steric interactions from nearby substituents and other chemical interactions pushing the amide group out of plane. Therefore, a set of 62 1D torsions were selected from Generation 1 TorsionDrive data sets, and 2347 optimized geometries and 532 Hessian targets were selected from Generation 2 data sets. The explicit target files used in training Parsley 1.3.0 and the ForceBalance output could be found from the release tarball for this force field.^92^ The 1.3.0 training data set was smaller than that used for 1.2.0 or 2.0.0 and contained molecules chosen from both Generation 1 and Generation 2 sets, whereas 1.2.0 and 2.0.0 force fields used only Generation 2 sets.

For training valence parameters in Sage 2.0.0, around 3663 optimized conformer geometries and 713 1-dimensional torsion scans were used as training targets from Generation 2 data sets, shown in Table 1. For any given molecule, we use no more than 10 optimized conformers in fitting, so that molecules with a higher number of conformations were not weighted higher than other chemistries. A greedy selection algorithm was applied to select the conformers which were most distinct as measured by their RMSD.

For all force fields trained above, a few filtering steps were common. Molecules with changes in connectivity between initial and final structures after geometry optimization were filtered out from the training targets. Most of these include molecules with an intramolecular proton transfer occurring during the optimization. Additionally, for *torsion-profile* targets, molecules with strong intramolecular hydrogen bonds were excluded based on Baker–Hubbard criteria as implemented in MDTraj.^93,94^ Molecules with intramolecular hydrogen bonds were excluded to avoid training against conformations with internal H-bonds that, though very strong in the gas-phase, are less dominant in the condensed phase.

#### 2.3.3 Valence Parameters Trained in Sage 2.0.0

Valence parameters that were applied to at least five molecules in the target QM data set were chosen for optimization. Parameters that are not chosen for optimization retain the same values as the Parsley 1.3.0 force field. With a new automated setup, using OpenFF-BespokeFit and OpenFF-QCSubmit, the set of training targets and the parameters to be optimized with Force Balance were stored in a json file for reproducibility. The data file, https://github.com/openforcefield/openff-sage/blob/2.0.0-rc.1/schemas/optimizations/vdw-v1-ms-v1-td-opt-v3.json, includes the selected optimized geometries and torsion scans tagged by their QCArchive record numbers and a list of SMIRKS patterns of valence parameters to be optimized.

The numbers of each type of valence parameters optimized were as follows:

- Harmonic bond stretches: 56 out of 88 total parameters were retrained.
- Harmonic angle bending: 33 out of 40 total parameters were optimized. In case of angles for linear bonds (e.g., triple bonds), only the force constant was optimized, keeping the equilibrium angle constant at 180°.
- Proper torsions: Force constants of 95 torsion parameters out of 167 total parameters were optimized. Torsion parameters (t165, t166, and t167 in Sage 2.0.0) that were used to describe linear substructures, such as in acetylene, were not optimized and they retain the value of zero for their force constants, as all enumerated rotatable bonds must be assigned force constants. We note that the number of Fourier terms was set separately for each torsion parameter, with the number of the Fourier terms chosen manually for each parameter based on chemical typing, with periodicities that were expected to give appropriate minima at appropriate dihedral angles as observed in QM torsion profiles. Neither the number of terms nor the periodicities were adjusted in fitting; only the force constants were varied in fitting.
- Improper torsions: There were 7 improper torsions, and none were optimized for the 2.0 release. Instead, they were held at the same values as in prior force fields, chiefly because torsions were deemed to be too broadly defined, i.e., covering too diverse a range of chemistries, and therefore in need of further refinement before refitting.

#### 2.3.4 ForceBalance Targets and Loss Function Definitions for Valence Term Training

ForceBalance^61^ is a force field optimizer which constructs an objective function taking into account the deviations in MM estimated properties with respect to reference QM properties. A very large number of approaches are possible in weighting the different components of the fit from QM to MM structures. We describe below the current OpenFF approach, and how the parameters and justifications have evolved over time. Although other approaches to fitting are possible and may be worth exploring, we have found that the approach reported here yields reasonable results that agree better—for both training and test sets—with benchmarks than our starting point force fields, and it performs better than any other fitting approach we have tried to date. However, we welcome experimentation from the scientific community, and our data sets and infrastructure are readily available to help facilitate such work.

The optimization procedure used for Sage was similar to that used for Parsley,^10^ where the goal was to minimize the deviations in internal coordinates for optimized geometries and to minimize the deviations in relative energies with respect to a reference QM torsion profile. Each QM optimized geometry of a molecule is called an *opt-geo* target. Consideration of the *opt-geo* target involves evaluating a sum of the deviations in internal coordinates of MM optimized geometry at the current set of MM parameters at each iteration with respect to the QM reference. The QM torsional profile of a molecule, in which a specific central bond is rotated on a grid of dihedral angles, is called a *torsion-profile* target. Evaluating a *torsion-profile* target involves taking the differences in relative energies between a MM generated torsion profile at the current set of MM parameters, at each iteration, with respect to QM relative energies, at each of the grid points of the torsion scan (eq 3).

For the optimized geometry targets, the objective function contribution is defined as
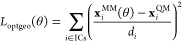
2where θ stands for force field parameters in the current iteration used in the MM calculation, **x**_*i*_^QM^ and **x**_*i*_^MM^ are the internal coordinates of the QM optimized reference minimum and the MM optimized geometry, respectively, and *d*_*i*_ refers to the scaling factors of 0.05 Å, 8°, and 20° for bond lengths, bond angles, and improper torsion angles, respectively. The values of dimensional scaling factors here were chosen based on chemical intuition given the size scale of typical atomic fluctuations, with the goal that each term in the objective function contributes similarly to the overall objective function and that fluctuations larger than “normal” in a particular coordinate would be penalized. Deviations in proper torsion angles were not included in this objective function since those were fitted with the torsion profile energetics by keeping the dihedral angle constant on a grid of angles and fitting torsion profiles solely with optimized geometries might introduce numerical artifacts. This is because equilibrium geometries do not provide information about the higher energy regions of energy landscapes that are encountered in a dihedral rotation.

Improper torsion parameters were not retrained but instead were held constant, as noted above. However, deviations in improper angles were included in the *opt-geo* target objective function to minimize discrepancies in improper angles of MM optimized structures, since planarity is dictated by a balance of angle bending parameters and our (unchanged) improper torsion parameters. Without including some metric of planarity in the fits, angle parameters would be free to change in a way which bends planar groups out-of-plane without this contributing to the objective function, so we included this metric in the *opt-geo* objective function. The situation should be improved in subsequent work as we introduce a more chemically specific set of improper torsion parameters and begin to specifically refit these.

For *torsion-profile* targets, relative energies were calculated with respect to the minimum energy on the grid for the reference QM torsion profile, as well as MM torsion profile. While evaluating the MM torsion profiles, to avoid large structural changes a harmonic positional restraint with a force constant of 1 (kcal/mol)/Å^2^, was applied on atoms not involved in the torsion. The energy contributions from the restraints were removed before comparing with the QM energies. The four atoms involved in the torsion were constrained during MM optimization.^10^
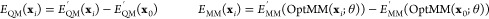
3where the primes indicate the absolute energies at each grid point *i*, and the weighted differences in relative energy profiles serve as the objective for minimization:
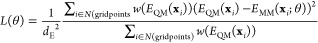
4where **x**_*i*_ represents the coordinates of the *i*th conformer, the 0th conformer is the minimum energy conformer in respective potential energy landscapes, θ is the force field parameter set at that iteration, and OptMM(**x**_*i*_, θ) corresponds to the MM energy obtained via constrained minimization and *d*_E_ = 1 kcal/mol is a conversion factor to make the sum over deviations dimensionless.

The weights *w*(*E*_QM_) in eq 4 were applied to prioritize matching the torsion profile near the minima rather than the barriers. Boltzmann sampling favors low energy regions of state space, so agreement of potentials in low energy regions is typically of higher importance than agreement in high energy regions for thermodynamic measures. The choice of weights as a function of energy deviation from the minima *E*_QM_ was similar to that of Parsley and was based on a prior study that used a Boltzmann distribution with *T* = 2000 K (*k*_B_*T* ≈ 4.0 kcal/mol) to weight energies in torsion fitting and found that these weights led to the best performance relative to other choices.^95^ Thus, we used an energy cutoff as a function of QM energy difference from the minima *E*_QM_ as follows:
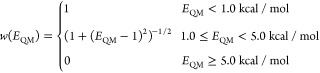
5

The total objective function was a weighted sum of normalized contributions of both *opt-geo* and *torsion-profile* targets,
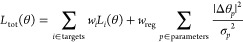
6where *w*_*i*_’s were weights of 0.1 and 1 for *opt-geo* and *torsion-profile* targets, respectively. These weights were chosen such that the final contributions from each of the different targets stay on the same scale, on the order of 1.0 after weighting, and do not skew the optimization in favor of either of the objective function components. The corresponding loss functions *L*_*i*_ for each of the targets were as defined in eqs 2 and 4. *w*_reg_ is the regularization penalty weight, whereas Δθ_*p*_ is the deviation from initial parameter values. The denominator σ_*p*_ gives the penalty for the parameters to deviate significantly from the starting point, for each type of parameter, and is described in more detail in the next subsection.

#### 2.3.5 Regularization of the Parameters

Regularization was used in the optimization to achieve smooth convergence and to prevent the parameters from moving too far from the starting point to potentially unphysical local minima. For this optimization, the starting point was the 1.3.0 force field. Regularization with a harmonic term can be seen in a Bayesian sense as imposing a Gaussian prior on each parameter with mean of the starting point and standard deviation equal to the regularization scale σ_p_. We used a data-driven approach to determine the regularization scales used in the fitting procedure. The distribution of parameters in SMIRNOFF99Frosst for each parameter type was first plotted. Since the distributions were not bell-shaped (as seen in SI Figure S4a), we decided to use IQR (interquartile range) values instead of standard deviation of the distributions to set the regularization scale σ_p_, given in Table 4.

**Table 4:** Regularization Scales Used in Optimizing Force Field Parameters with ForceBalance^a^

parameter	regularization scale σ_p_
bond force constant *K*_r_	100 kcal/(mol/Å^2^)
bond equilibrium length *r*_0_	0.1 Å
angle force constant *K*_θ_	100 (kcal/mol)·rad^2^
equilibrium angle θ_0_	20°
proper torsion barrier height *K*	1 kcal/mol
vdW well depth ϵ	0.1 kcal/mol
vdW minimum *R*_min/2_	1 Å

a Regularization helps when we are training on smaller data sets, as the final optimized parameter values apply generally to a wider chemical space. The values were chosen based on chemical intuition and also by looking at the distribution of parameter values in SMIRNOFF99Frosst.

The overall optimization of the force field through this process was considered to have converged when it satisfied at least two out of the following three criteria: total objective function value, including the regularization penalty, to reach a value ≤0.1; the norm of gradient on parameters to reach a value ≤0.1; and the optimization step size to be ≤0.01.

### 2.4 Benchmarking Methods

Calculations of solvation free energies (Δ*G*_solv_) used the YANK alchemical simulation software package, version 0.25.2^96^ and the same OpenFF Evaluator workflow as used in Boothroyd et al.^70^ These calculations used a thermodynamic cycle that involved 2 simulation steps: (1) the removal of a solute molecule from a box of solvent and (2) the annihilation of a solute molecule from a vacuum box. For step 1, the simulation box contained 2000 molecules of solvent and a single molecule of solute. The solute was removed along an alchemical pathway which gradually turned off the nonbonded interactions along a soft-core alchemical schedule.^97^ The implementations of the alchemical pathway and values of λ are handled by the openmmtools software package version 0.20.3.^98^

We also assessed the performance of the newly fitted Sage force field in relative binding free energy calculations based on molecular dynamics simulations following suggested best practices for benchmarking binding affinities.^99^ Relative binding free energies were calculated employing alchemical perturbations between pairs of ligands in water and the protein complex. These calculations employed a nonequilibrium workflow based on GROMACS and *pmx* as described previously.^72,100^ For the ligand molecules, the Sage 2.0.0 force field was used. The protein was parametrized with the AMBER ff99sb*-ILDN force field,^27,28,101^ and a TIP3P explicit water model^51^ was employed. We chose AMBER ff99sb*-ILDN as the protein force field because Parsley and Sage are essentially AMBER-family force fields and should be compatible, or nearly so, with AMBER protein force fields.^10^ The water model was chosen as TIP3P due to the widespread use of this water model with the AMBER family of protein force fields and because TIP3P was used in fitting to condensed phase properties described in this paper. To mimic physiological conditions, ions (150 mM NaCl^102^) and additional counterions to neutralize the system were added to the dodecahedral simulation boxes.

The analysis workflow used for analyzing the calculations is available in Hahn et al.^103^ The statistics in this workflow were calculated using Arsenic (repackaged as Cinnabar),^104^ which is a package implementing best practices for consistently calculating statistics and reporting results from relative binding free energy calculations. The test set consisted of 22 different series of congeneric ligands binding to 20 protein targets with a total of 599 ligands. All calculations used the input structures provided in the protein–ligand-benchmark repository.^105^ More detailed discussion of the workflow, the employed parameters, analysis, and benchmark sets can be found in the Supporting Information.

## 3 Results

### 3.1 Changes in Parameters As a Result of Training

#### 3.1.1 Changes in LJ Parameters

**Figure 2:**
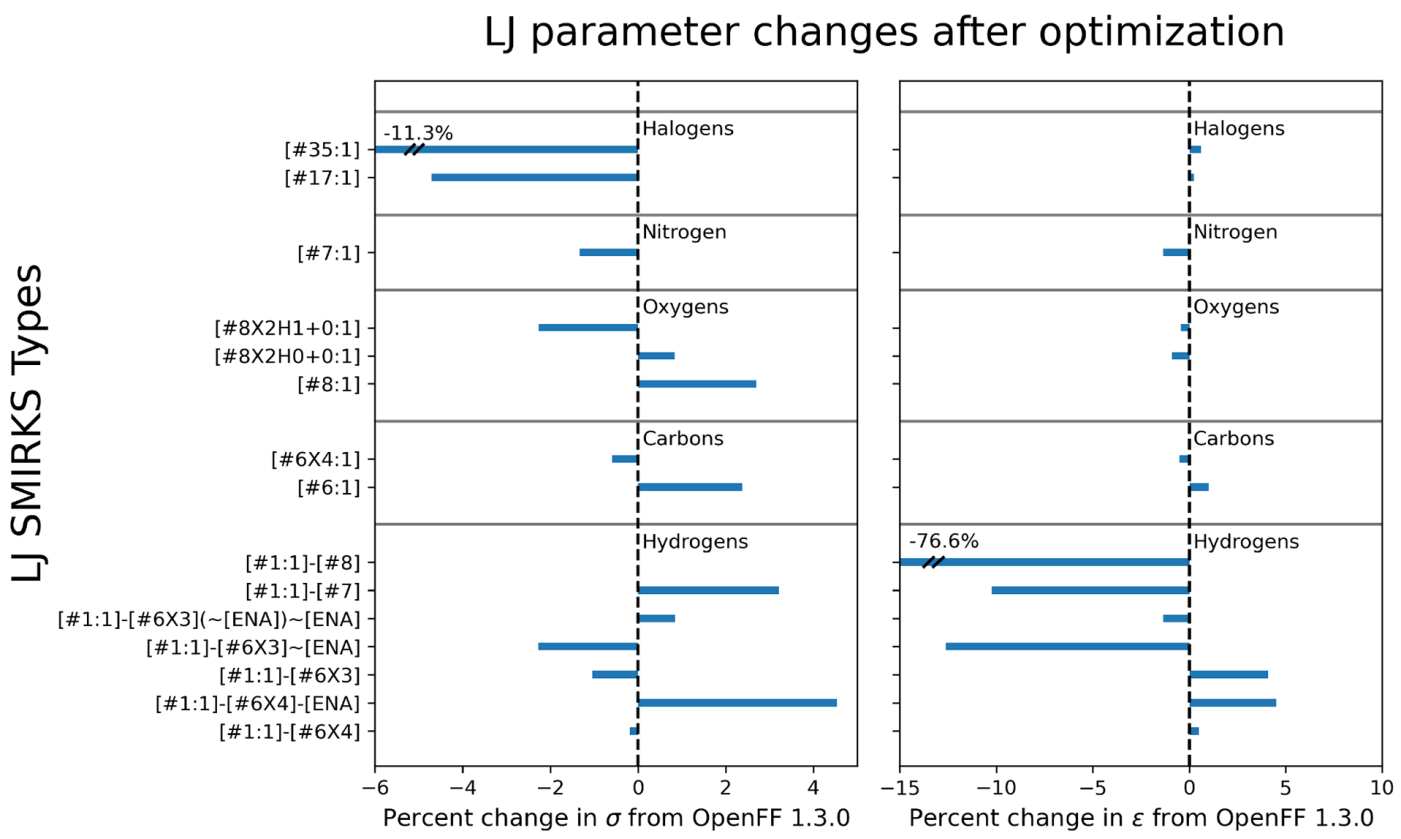
Changes in LJ parameter values for refit LJ types. Plot shows % change of σ (left panel) and ϵ (right panel) for each of the 15 LJ types refit in Sage 2.0.0. In this plot, “ENA” refers to electronegative atom, corresponding to the SMIRKS string [#7,#8,#9,#16,#17,#35].

Optimization against densities and enthalpies of mixing led to significant changes in many of the LJ parameters after refitting, detailed in Figure 2. These changes, as detailed below, provide clear evidence that using condensed phase mixture properties in the optimization can have significant effects on force field performance.

**Figure 3:**
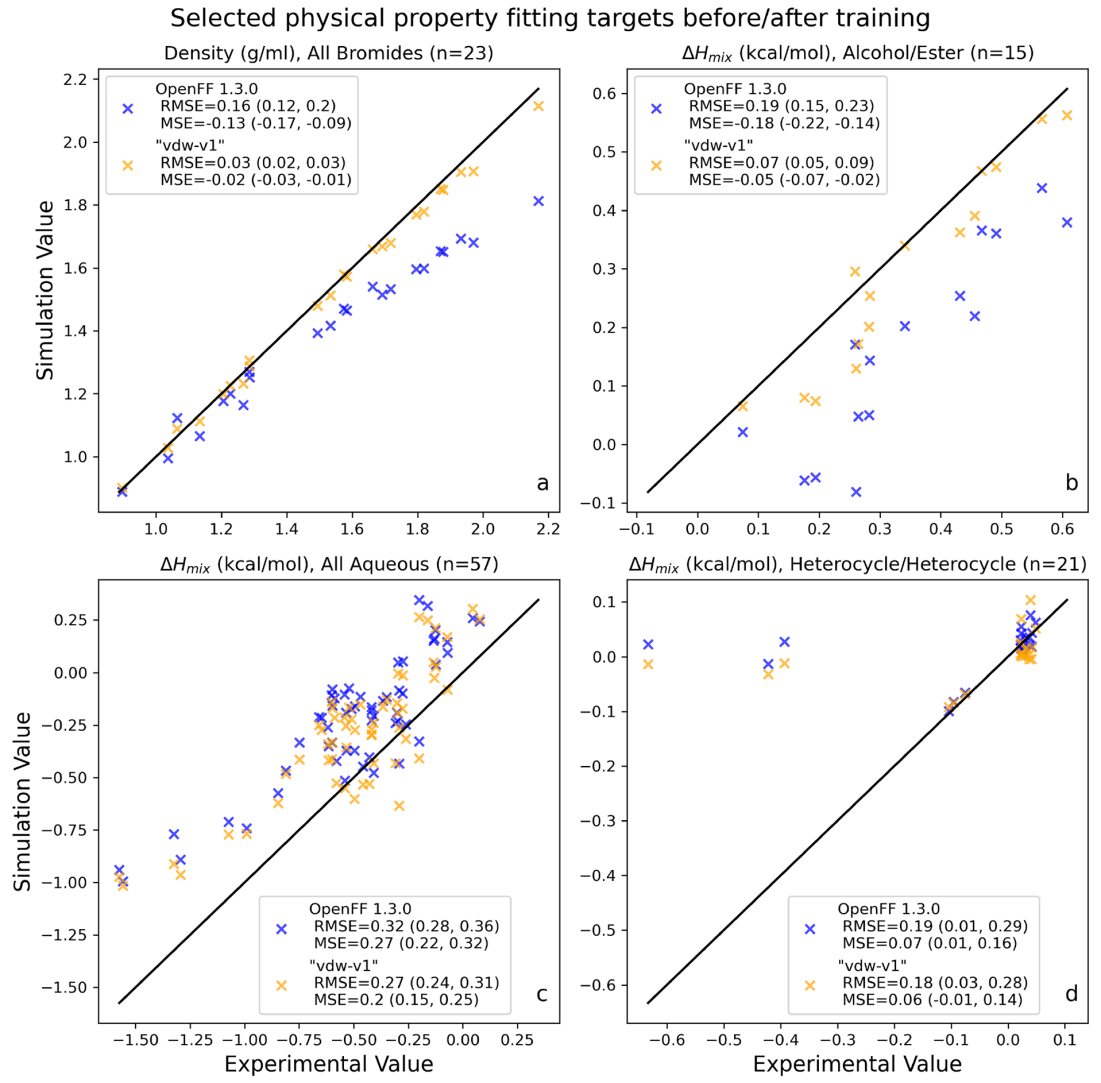
Selected categories of physical property training data, before and after LJ optimization. These plots show parity between experiment and simulation for physical properties in the training set, before (Parsley 1.3.0) and after (*vdw-v1*) LJ training. This shows how LJ refitting impacts the computed properties, before refitting valence terms, so that the effects of LJ refitting are isolated from other factors. “MSE” in the panel legends refers to the mean signed error (bias) of the data set. This plot highlights successful refits as well as challenges remaining. Panel a shows correction of systematic error in bromide density prediction after reduction in [#35:1]*R*_min/2_. Panel b shows correction in Δ*H*_mix_ of alcohol/ester mixtures after training. Panel c shows significant overprediction of Δ*H*_mix_ for aqueous mixtures, which is reduced but not eliminated in Sage 2.0.0. Panel d shows Δ*H*_mix_ for mixtures of heterocycles. The three data points in the upper left corner are mixtures of pyrrole and pyridine, which have large negative values of Δ*H*_mix_ that our force field does not currently reproduce, potentially solvable with lone pairs on heterocycle nitrogens. Values in parentheses indicate bootstrapped 95% confidence intervals.

Overall, almost all new values of LJ *R*_min/2_ and ϵ are within ±5% and ±10% of the Parsley 1.3.0 values, respectively. Exceptions include the *R*_min/2_ for [#35:1] (Br), which decreased significantly. This decrease is associated with a correction in bromide densities, which were generally underpredicted relative to experiment in Parsley 1.3.0. The reduction in *R*_min/2_ and therefore molecular volume leads to an increase in densities after the optimization that corrects the underprediction. The dramatic correction in densities for bromides and bromide-containing mixtures after optimizing the LJ parameters is illustrated in Figure 3, panel a.

Figure 3 shows performance on the training set before and after LJ refitting, focusing on several notable cases where the optimization was successful or unsuccessful; a similar plot for all training data is available in SI Figure S1. Figure 3 shows the force field with refitted LJ parameters but unchanged valence parameters, referred to as *vdw-v1*.

This intermediate refitted force field shows improvement on the densities and enthalpy of mixing measurements in the training set. Density root mean square error (RMSE) is reduced from 0.041 g/mL (95% CI 0.033, 0.049) in Parsley 1.3.0 to 0.017 g/mL (0.015, 0.019) in *vdw-v1*; percentage error is reduced from 2.13% (95% CI 1.93, 2.33) in Parsley 1.3.0 to 1.28% (1.18, 1.38) in *vdw-v1*. For enthalpy of mixing, RMSE is reduced from 0.65 kJ/mol (95% CI 0.59, 0.72) in Parsley 1.3.0 to 0.53 kJ/mol (0.47, 0.60) in *vdw-v1*; percentage errors for enthalpies of mixing are not computed because many heats of mixing are near zero, resulting in numerical inconsistencies. The performance of *vdw-v1* on liquid state properties is expected to be nearly identical to Sage 2.0.0, as these properties are most dependent on LJ parameters and electrostatics, which are unchanged between *vdw-v1* and Sage 2.0.0, and the valence parameters, upon which these properties only weakly depend, are only optimized slightly in the final fitting. Additionally, the performance of *vdw-v1* on the solvation/transfer free energy benchmark set described in section 3.2 (shown in Supporting Information Table S1) was not statistically different than that of Sage 2.0.0. The data in this table is presented not as the final force field, as the valence parameters in *vdw-v1* were not yet reoptimized in the presence of new van der Waals parameters, but to illustrate the direct effect of LJ refitting on performance with mixture data.

The other substantial percent change is the ϵ for the hydroxyl hydrogen [#1:1]-[#8] type, which is less notable on an absolute scale, as it is reduced from 5.27 × 10^–5^ kcal/mol to 1.22 × 10^–5^ kcal/mol. This parameter was discussed previously in Mobley et al.,^37^ and its value is essentially designed to be “small but non-zero”, in order to avoid unphysical effects; it did not originally result from a fit to condensed phase properties. We therefore do not assign a significant physical meaning to the reduction of this value.

Among the other LJ types retrained, we see a notable reduction for ϵ of the [#1:1]-[#7] type, which is for hydrogens attached to nitrogens, as well as the [#1:1]-[#6X3]∼[ENA] type, which is associated in this training with aromatic heterocycles containing nitrogens. “ENA” in this context refers to the SMIRKS string [#7,#8,#9,#16,#17,#35] and represents a bond to an electronegative atom. These changes are likely made to reduce overpredictions in densities and enthalpies of mixing for many nitrogen-containing compounds, although they were not entirely successful. We also note that while we cover several nitrogen chemistries, there is only one nitrogen LJ type, and so adjustment of these parameters (as well as the [#1:1]-[#6X4]-[ENA] type) is the main method of accounting for different nitrogen environments.

Additionally, we see a reduction in *R*_min/2_ for [#8X2H1+0:1], which corresponds to a hydroxyl oxygen. In previous work,^50^ we hypothesized that this change might be related to improved treatment of mixtures of alcohols and hydrogen bond acceptors like esters and ketones. We see those same improvements here, where alcohol/ester mixtures are initially underpredicted relative to experiment, but that underprediction is reduced after retraining, as shown in Figure 3, panel b.

Aside from parameter changes, another notable trend from the training targets is the systematic overprediction of enthalpies of mixing for aqueous mixtures, as shown in Figure 3, panel c. That this systematic overprediction was slightly reduced, but not corrected, points to a larger issue with the aqueous mixtures. Since the water model used in refitting (TIP3P) was not refit, the optimization algorithm was likely unable to eliminate this error by adjusting the nonaqueous components alone. This indicates that in order to significantly improve aqueous mixtures, we would likely need to retrain a water model in a future version of the force field or pursue a more aggressive optimization of these aqueous physical properties.

Examining training data split out by chemical context allows us to detect failures and rapidly propose solutions for future releases. One prominent example is illustrated in Figure 3, panel d. The three data points in the upper left corner of this plot represent mixtures of pyrrole and pyridine at several concentrations. While the experimental Δ*H*_mix_ of these mixtures should be significantly negative, simulations with both Parsley 1.3.0 and Sage 2.0.0 produce a Δ*H*_mix_ of roughly 0, indicating nearly ideal mixing. A potential reason for this failure to capture the molecular behavior is the force field’s inability to correctly capture the orientation of the pyridine lone pair. A possible solution is the introduction of an off-site charge for pyridine; preliminary tests with off-site charges show promising initial results in correcting this issue.^106^

#### 3.1.2 Changes in Valence Parameters

As mentioned in section 1.2.1, the LJ parameters were optimized first, and the resulting force field from step 1 was used as input for valence parameter training. With the optimization procedure described in section 2.3, the ForceBalance run satisfied the convergence criteria after 14 steps, and the drop in objective function value is shown in SI Figure S5.

Examining the changes in valence parameter values, we find that bond lengths barely changed between Parsley 1.3.0, the starting point for this optimization, and the final optimized values in Sage, with none changing more than 2%. There are two bond parameters with a more significant change in force constant values, b56 ([#16X4,#16X3!+1:1]-[#6:2]), which changed by 13%, equivalent to +69 (kcal/mol)/Å^2^, and b57 ([#16X4,#16X3:1]∼[#7:2]) with a 24% change in value or +142 (kcal/mol)/Å^2^. For all other bond parameters, changes in bond force constant values are around 5% or less.

**Figure 4:**
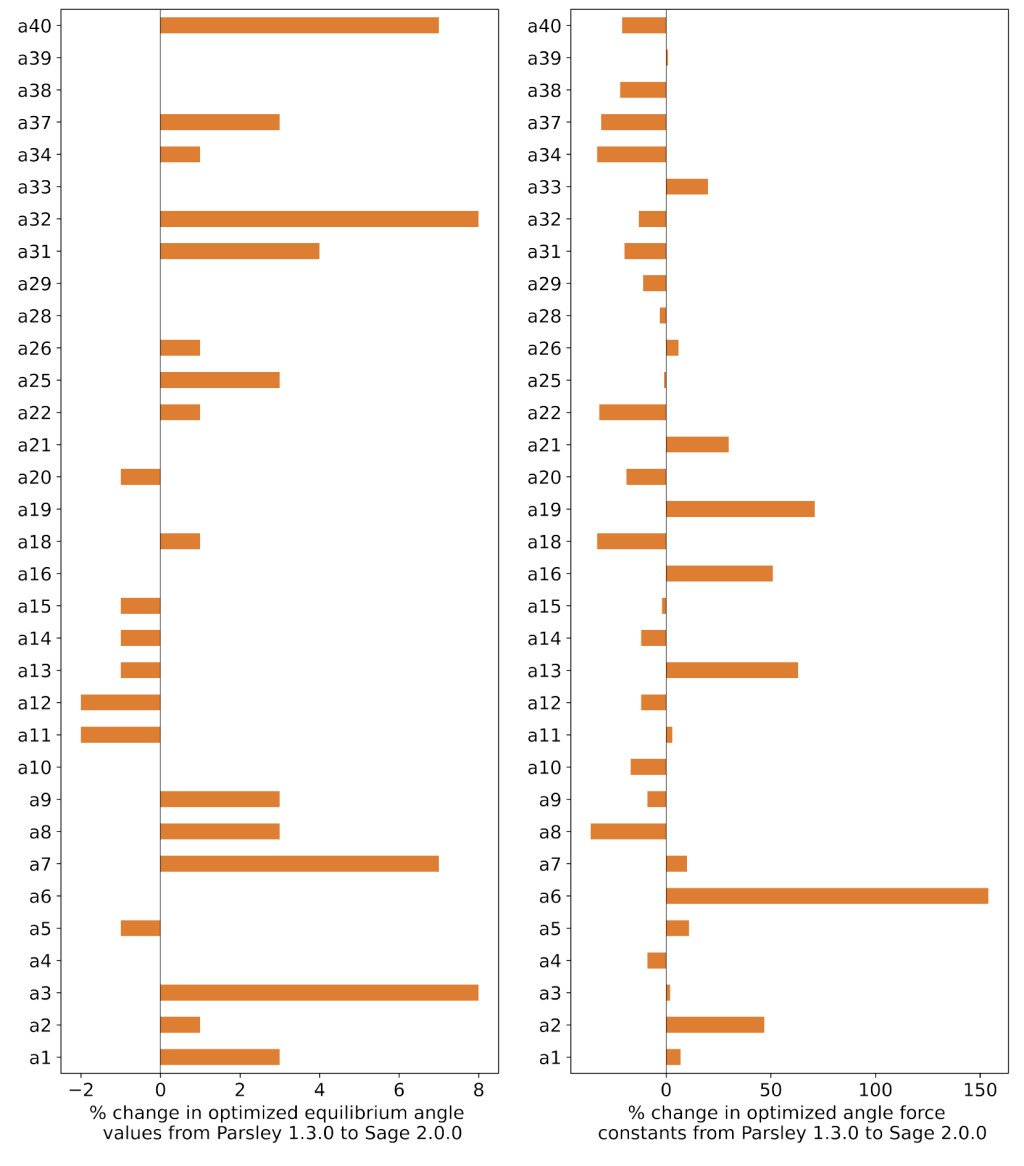
Percent change in angle parameters (angle θ, force constant *k*) from the starting points in Parsley 1.3.0 to the optimized values in Sage 2.0.0. The maximum change in equilibrium angle values was 10.16° for the parameter a40 ([*:1]∼[#15:2]∼[*:3]), which was a change of 7%. The maximum change in angle force constant values was 78.74 (kcal/mol)/rad^2^ for the parameter a19 ([*:1]–[#7X4,#7X3,#7X2–1:2–[*:3]), which was a change of 71%. The parameter IDs were with respect to the notation in Sage, and SMARTS strings corresponding to each parameter id are given in SI Table S3.

For angle parameters, the equilibrium angle values again barely changed, with all changes less than 8%. There were significant changes in some angle force constants, as high as 154%, for *k* of a6 ([#1:1]-[*;r3:2]∼;!@[*:3]), and these are shown in Figure 4. Changes in angle force constants are expected as they are coupled strongly with torsions, which are mainly affected by changes in LJ parameters.

Torsion parameters are the most flexible, and it is difficult to derive any insights by looking at the change in magnitude of force constants of torsion parameters. In general, torsional energy contributions are lower magnitude terms and a distribution of force constants with most values near zero is not unexpected, which can be seen SI Figure S6. In this figure, distributions of torsion force constants for Parsley 1.3.0, the starting point for the fit, and Sage 2.0.0 overlap pretty well with a peak near zero. Benchmarking torsion profile energetics and dihedral deviations with respect to QM geometries is a better way to assess torsion parameter quality, which we discuss in section 3.3.

### 3.2 Benchmarking Results: Solvation Free Energies

A set of solvation free energies (Δ*G*_solv_) served as a target to evaluate the performance of Sage 2.0.0 on condensed phase properties. This data set consists of (1) 87 solvation free energies for small molecules in aqueous solution from the FreeSolv database,^53^ referred to here as aqueous solvation free energies (Δ*G*_solv_(aq)), and (2) 382 solvation free energies of small molecules in nonaqueous solution from the MNSol database,^107^ referred to here as solvation free energies (Δ*G*_solv_(nonaq)). The uncertainties of the Δ*G*_solv_(aq) measurements from FreeSolv are generally 0.6 kcal/mol or below, and the uncertainties for the Δ*G*_solv_(nonaq) values in MNSol are listed as 0.2 kcal/mol for neutral solutes. The data sets are available at https://github.com/openforcefield/openff-sage/tree/main/data-set-curation/physical-property/benchmarks/data-sets.

We also computed aqueous to nonaqueous transfer free energies (Δ*G*_trans_(aq → nonaq)) from these reference data, where Δ*G*_solv_ values for a single solute are available in multiple solvents. Δ*G*_trans_(aq → nonaq) can be calculated from its individual components, as shown in eq 7.
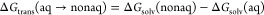
7Using eq 7 and the test data from MNSol and FreeSolv, we calculated reference transfer free energies Δ*G*_trans_(aq → nonaq) for 313 systems, where a system consists of a solute, an aqueous solvent, and a nonaqueous solvent.

Each set of free energies was calculated with Parsley 1.3.0, Sage, and GAFF 2.11 in order to provide comparisons to other widely used small molecule force fields. In each case, TIP3P is used as the water model for the solvent phase in aqueous mixtures and partial charges were assigned with the AM1-BCC method. For clarity, we will refer to the resulting GAFF force field as GAFF 2.11/AM1-BCC. While the recommended charge model for GAFF 2.11 is RESP, and others have indicated that GAFF 2.11/RESP often offers more accurate predictions than GAFF 2.11/AM1-BCC,^42^ we find that AM1-BCC charges are a reasonable charge model with significantly lower computational expense than RESP for large data sets.^108^ We also note that a new AM1-BCC-like charge model for GAFF 2.11, ABCG2,^109^ was recently developed; this charge model may offer improved performance on this benchmark set but is not yet publicly available.

To measure the improvement in performance due to the refit, we employ the *mean shift* performance metrics developed in Boothroyd et al.^50^ with Parsley 1.3.0 as the baseline. The mean shift metric, described in eq 8, measures how much the average error (relative to experiment) of a prediction changes when moving from one force field to another. In essence, it is the difference in unsigned errors between the refitted force field (Sage 2.0.0) and the reference force field (Parsley 1.3.0) for the physical property calculation for each molecule, averaged over the test set.
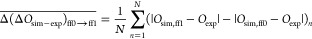
8

Here, *O*_sim_ is the simulated value of the observable, and *O*_exp_ is the experimental value of that same observable. A negative value of the mean shift indicates that predictions with Sage are improving over Parsley 1.3.0, whereas a positive value indicates that the predictions are regressing. We present this metric along with the kernel density estimate of the distribution of shifts, to visualize changes in improvement over the training sets. Kernel bandwidths are set to the defaults in the scipy package.^82^

**Figure 5:**
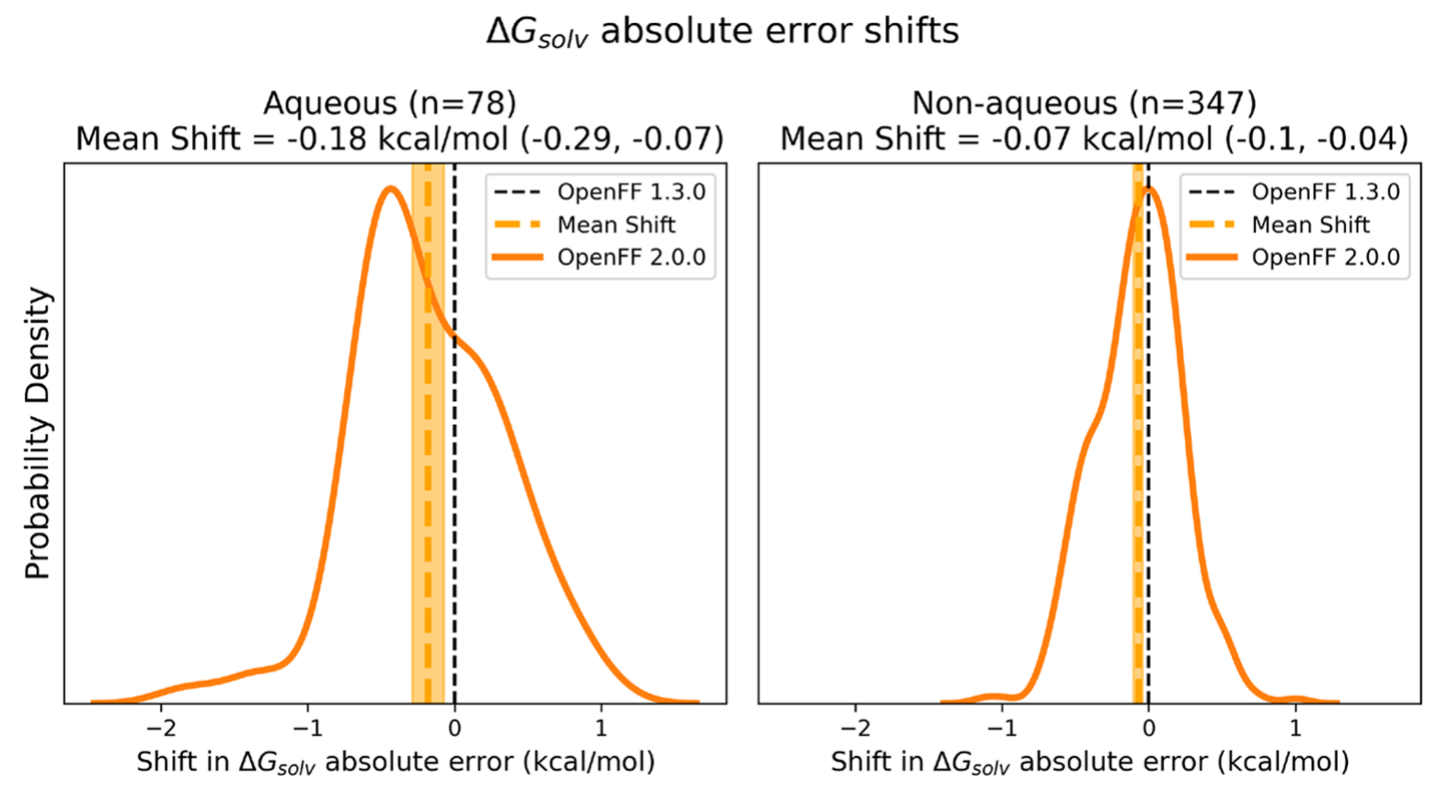
Mean shifts in absolute error indicate Sage has improved performance relative to Parsley 1.3.0 on both aqueous and nonaqueous Δ*G*_solv_. The plot shows the distribution of shifts in errors in Sage(OpenFF 2.0.0) relative to those in Parsley 1.3.0, as well as the mean shift in absolute error of Sage relative to Parsley 1.3.0 with 95% confidence intervals bootstrapped over pairs of molecules (“mean shift”). Here, more negative shifts indicate more accurate results. The performance of Parsley 1.3.0 is shown as a vertical line at ΔΔ*G*_solv_ = 0 (Parsley 1.3.0). Left panel shows performance on aqueous targets; right panel shows performance on nonaqueous targets.

Figure 5 shows that the mean shifts are negative (indicating improvement) and statistically significant for both aqueous and nonaqueous Δ*G*_solv_, though the effect is most pronounced for aqueous solvation. This indicates that Sage significantly improves predictions of Δ*G*_solv_ compared to Parsley 1.3.0. Note that the number of Δ*G*_solv_ measurements in the comparison is slightly lower than the total number in the test set; this is due to several Δ*G*_solv_ simulations that failed or had errors, and the comparison is only done on the set of free energy calculations which were successful with both force fields.

Although the mean bias is clearly improved, the distribution shows that a minority of the calculations were worse with Sage. An important question is what portion of the shifts are due to changes in the force field versus being due to statistical uncertainty in the simulations. To estimate the proportion of variance due to force field changes, we performed a deconvolution analysis, fitting a Gaussian distribution to the distribution of shifts and assuming the simulation error is Gaussian with 0 mean, and a standard deviation equal to the average propagated simulation uncertainty of a shift. Outlier analysis indicated that the Gaussian assumption is reasonable, with 95% of points (across both nonaqueous and aqueous Δ*G*_solv_) falling with the 2-sigma limit vs the expected 95.4%. Using these assumptions, we estimate the percentage of variance due to force field changes to be 85% for nonaqueous Δ*G*_solv_ and 96% for aqueous Δ*G*_solv_, with the rest due to statistical noise. This means the changes due to optimization are primarily due to shifting of force field error from one set of molecular solvations to another but with an overall reduction in total error.

**Figure 6:**
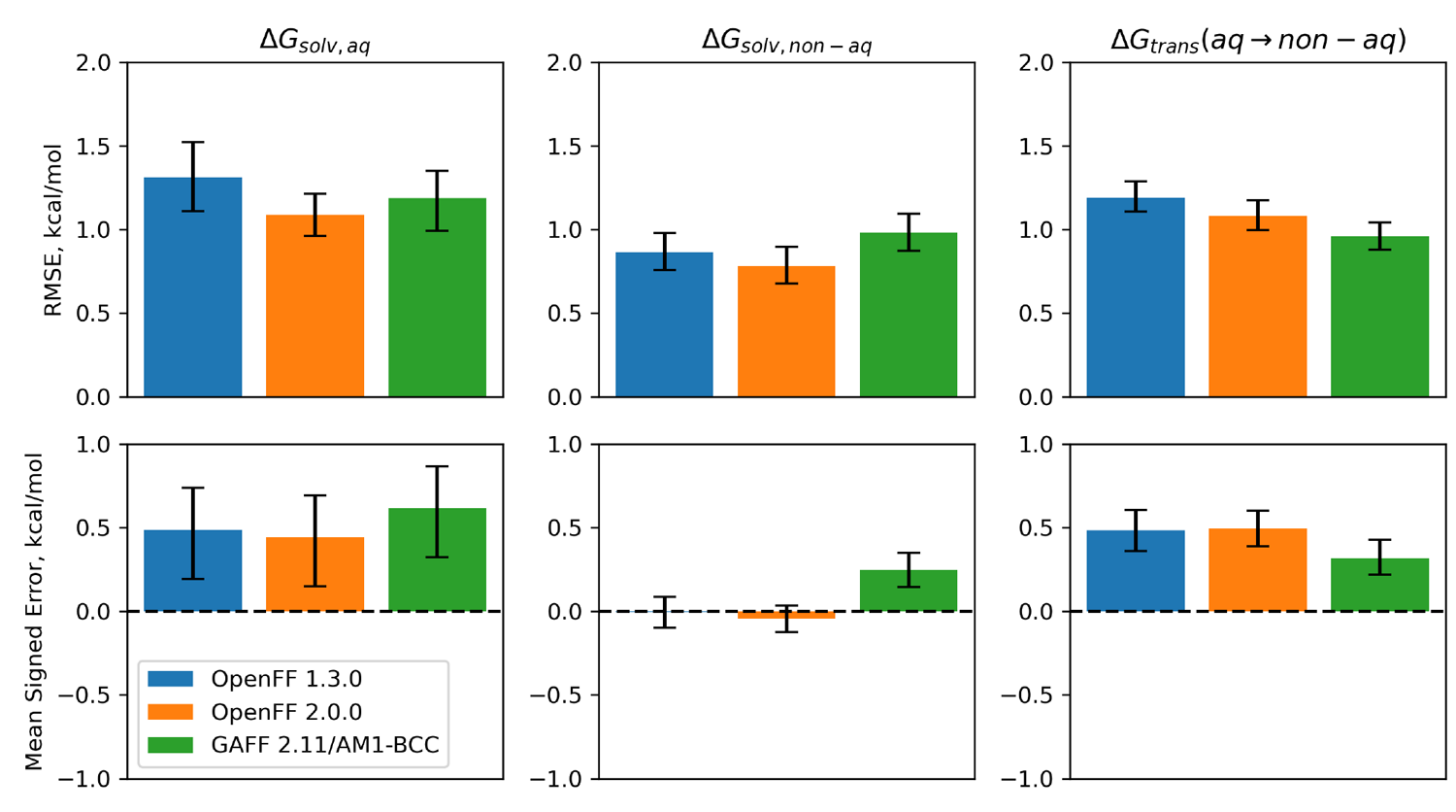
Benchmarks against small molecule solvation and transfer free energies. Benchmarks for Parsley 1.3.0, Sage 2.0.0, and GAFF 2.11/AM1-BCC against solvation free energies (aqueous, left column, and nonaqueous, middle column) and transfer free energies (right column) are shown. Top panels show RMSE against experimental free energies, with bootstrapped 95% confidence intervals. Bottom panels show mean signed error against experiment, which is less sensitive to individual outliers, with bootstrapped 95% confidence intervals.

Figure 6 (data also shown in Supporting Information Table S1) compares performance between Parsley 1.3.0, Sage 2.0.0, and GAFF 2.11/AM1-BCC, a widely used small molecule force field, paired with the AM1-BCC a fast charge model generally considered to be sufficiently accurate for pharmaceutical applications. In addition to calculating benchmarks on Δ*G*_solv_ for aqueous and nonaqueous solvents, we use the results of those calculations to calculate aqueous to nonaqueous transfer free energies (Δ*G*_trans_(aq → nonaq)) for solutes that have measurements of Δ*G*_solv_ for water and nonaqueous solvents. Transfer free energies are a useful benchmark target for a small molecule force field because they are analogous to the process of a small molecule ligand being transferred from an aqueous bulk phase to a nonaqueous binding pocket.

For the aqueous Δ*G*_solv_ test set, Sage is slightly improved over Parsley 1.3.0 and comparable to GAFF 2.11/AM1-BCC. A portion of the improvement in Sage relative to Parsley 1.3.0 is likely due to the inclusion of aqueous mixtures in the LJ training set. For the nonaqueous Δ*G*_solv_ test set, Sage again performs slightly better, with RMSE lower than Parsley 1.3.0 or GAFF 2.11/AM1-BCC. Both OpenFF force fields have a significantly lower bias (measured as mean signed error) when compared to GAFF 2.11/AM1-BCC.

While Sage 2.0.0 performs slightly better on both aqueous and nonaqueous Δ*G*_solv_, GAFF 2.11/AM1-BCC has the best performance for Δ*G*_trans_(aq → nonaq). This is apparently due to a cancellation of error between aqueous and nonaqueous solvents in GAFF 2.11/AM1-BCC; in both environments, solvation free energies are overpredicted relative to experiment. In Sage, aqueous solvation free energies are overpredicted in aqueous solution but not nonaqueous solution. This indicates that GAFF 2.11/AM1-BCC’s superior prediction of Δ*G*_trans_(aq → nonaq) benefits from a cancellation of error between aqueous and nonaqueous phases. This suggests that future OpenFF force fields could benefit from the training of a companion water model.

Finally, we note caution in the direct comparison between GAFF2 and OpenFF, as partial charge generation protocols can be different, such as the difference between RESP and AM1-BCC, or between different AM1-BCC implementations.^42^ Some of the differences in GAFF2 versus OpenFF performance presumably result from differences in how they were trained; however, it is difficult to comment on this, as the GAFF2 series training and test procedures and data sets have not yet been disclosed.

### 3.3 Benchmarking Results: Force Field Benchmarks Relative to QM Data

**Figure 7:**
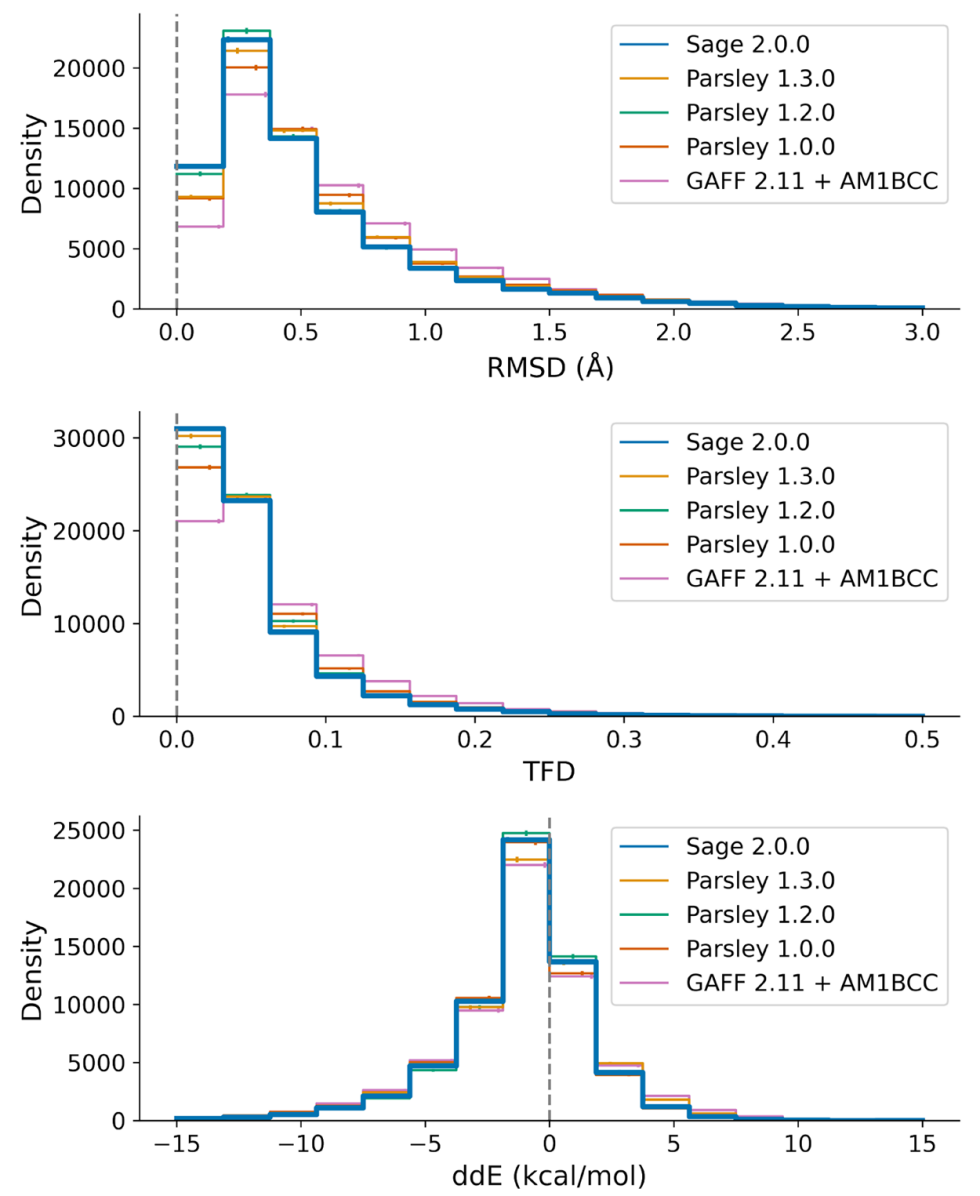
Step plots showing improvement in RMSD and TFD of optimized conformer geometries and a closer match of ΔΔ*E* with previous generations of force fields. The error bars are bootstrapped errors for each bin. The force field Sage 2.0.0 is highlighted with a bold line, while other force fields are shown with narrower lines. Overall, Sage appears to do substantially better than previous releases based on geometric measures (RMSD, TFD), while performing only marginally worse than the best prior force field (Parsley 1.2.0) on ΔΔ*E*. The slight drop in ΔΔ*E* values can likely be attributed to the reduced set of *opt-geo* training targets used to train Sage.

Overall, benchmarking of quantum chemical geometries and energetics shows Sage substantially improves results relative to our earlier force fields, with particularly substantial improvements by geometric measures (Figure 7), as we further discuss below. Here, we provide details of our benchmark data set and then overall performance relative to quantum chemical data, and finally we identify areas where the force field needs further improvement so these can be addressed in subsequent studies.

#### 3.3.1 Benchmark Data Set Composition and Parameter Coverage

Following a previous benchmark study,^38^ we compared performance of Sage 2.0.0 to earlier generation OpenFF force fields, as well as general small molecule force fields. Specifically, we use a set of QM optimized conformer geometries and energies to assess how well we can reproduce conformer energetics and geometries with MM parameters. A much larger data set of QM-optimized gas-phase geometries, named “OpenFF Industry Benchmark Season 1 v1.1” on QCArchive, is now used for benchmarking MM optimized geometries.^110^ We built this data set in collaboration with our industry partners to benchmark force fields more generally, and it consists of nearly 9847 unique molecules, and a total of 76713 conformers. The data set also has a wider distribution of charged entities than our training set, including formal charges of [−2, −1, 0, 1, 2]. The mean molecular weight of the molecules is 348 Da, and a maximum molecular weight is 1104 Da, showing a large range of molecular size. This data set is generated at B3LYP-D3BJ/DZVP, the same level of theory as our training data. Following conformer generation and geometry optimization, we processed this data set to filter out connectivity changes during optimization, cases with stereochemistry which cannot be perceived, as well as any calculation failures due to convergence issues. This filtering brings down the final set used in the benchmarking to 73301 conformers.

Although this data set is quite large, it consists mainly of drug-like molecules that are of interest to our industry partners. As a consequence, not all parameters are covered by the molecules in this data set, as Sage is general enough to represent chemistries which are rare in drug-like molecules. The following parameters are not applied on any molecule in this data set (SMARTS for each bond ID are given in SI Table S3):

- Bond parameters: b23, b29, b40, b47, b48, b49, b50, b55, b63, b66, b74, b75, b78, b79, b80, b81, b82, b83 (18 out of 88 parameters not covered).
- Angle parameters: a30, a35, a36 (3 out of 40 parameters not covered).
- Proper torsion parameters: t8, t63, t89, t102, t112, t113, t114, t164 (8 out of 167 parameters not covered)

The benchmark is therefore slightly skewed toward the most commonly occurring chemistries and may miss some exotic chemistries, such as bridgehead nitrogens, which occur frequently in the VEHICLe set of heterocyclic molecules. This set is also available in QCArchive.^111^ However, it covers more valence parameters than those trained in this iteration of the force field (refer to section 2.3.3 for more information).

#### 3.3.2 Global Metrics of Merit

As global metrics of merit, we use root mean squared deviation in geometries between MM optimized and QM optimized conformers (RMSD), torsion fingerprint deviation (TFD), and error in relative conformer energies (ΔΔ*E* or ddE) as described in a previous work by Lim et al.^38^ TFD is a weighted metric of deviations in dihedral angles which overcomes the limitations of RMSD.^112^ Only 24 molecules out of the whole set of 73301 molecules fail to generate TFDs; this happens when a molecule has no nonterminal rotatable bonds. Overall, errors in torsions are well captured by the TFD metric, and a lower TFD value means the geometry is close to the reference structure.

The error in relative conformer energies, ΔΔ*E*, is defined for the *i*th conformer in a molecule by
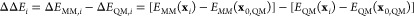
9where the relative conformer energies were calculated with respect to the QM minimum energy conformer (labeled 0th) within each molecule, We exclude the minimum energy conformer, which has a ΔΔ*E* of 0.0, in calculating ΔΔ*E* statistics so that final results were not skewed toward zero. We also note that the energies of the MM conformers were compared directly to their QM counterparts from which they were optimized.^38^ Such a direct comparison may result in higher errors since the MM optimized structure may be very different from the QM reference structure. However, a comparison of ΔΔ*E* with MM conformers that match to any of the QM conformers within an RMSD cutoff of 1 Å (shown in SI Figure S7) depicts the same trends as observed in Figure 7.

Improvements over generations of force fields can be seen in the step plots in Figure 7, where the population density in the bins closer to minimal error are increasing for RMSD and TFD metrics. In the case of ΔΔ*E* the histogram stays close to the best among earlier generations, Parsley 1.2.0. The slight degradation observed here could be due to the reduced number of optimized geometry targets used in training the force field; this reduction means that the overall fit places slightly less weight on energetic agreement relative to geometric agreement. In particular, there is a reduction of around 1082 optimized conformers between Parsley 1.2.0 and Sage 2.0.0 training sets, and a comparable performance is achieved with a reduced set of targets.

### 3.4 What Lies in the Outliers, a *Post Hoc* Study, and the Plan Ahead

We now examine what we can learn from the outliers in force field benchmarks relative to QM conformers and energetics and from analysis of various bond, angle, and torsion parameters applied in these outlier cases. In particular, an analysis of failures/poor performers will point the way forward for future work. Such future work may require generating new quantum chemistry data, improving parameter typing (e.g., assessing the possibility of parameter splits and the quality of new parameters), or adding more torsion periodicities. Subsequent work will explore each of these areas, and additional force field releases are planned once such improvements are ready.

#### 3.4.1 Bond and Angle Deviations

**Figure 8:**
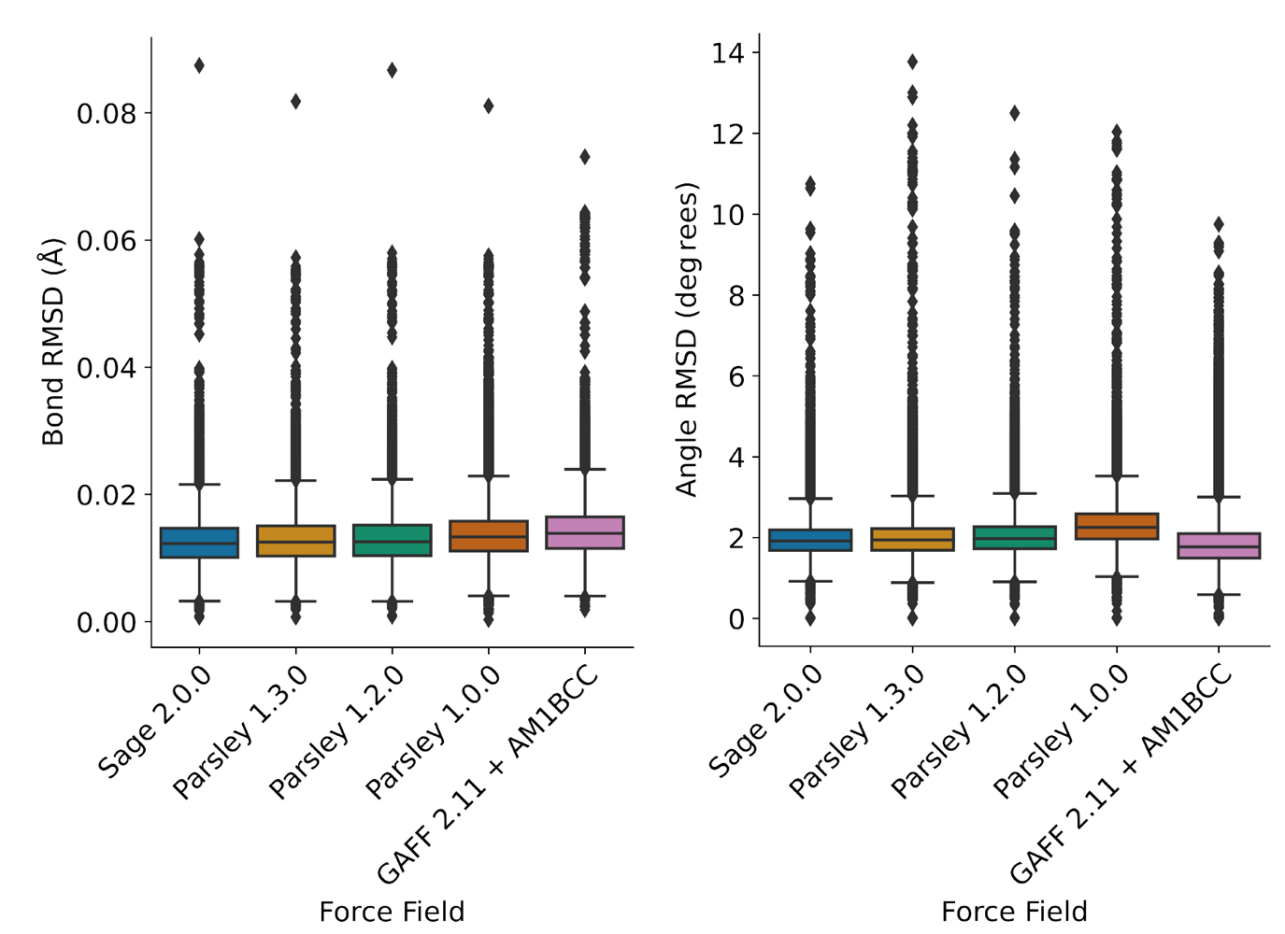
Box plots of the distribution of bond and angle RMSDs of each conformer for all the molecules in the OpenFF Industry Benchmark set. The edges of the boxes show the first and third quartiles and the whiskers are at 1.5× (interquartile range) away from the box edges. All bond RMSDs are less than 0.1 Å and all angle RMSDs are less than 11° using Sage 2.0.0.

The first set of granular metrics are the deviations in equilibrium bonds and angles, as close agreement with QM is necessary for bonded interactions. Unphysical structures with elongated bonds or shortened angles indicate a pathology in describing the chemistry. Distributions of bond and angle RMSD within each conformer with respect to the QM reference are shown as box plots in Figure 8. The bond lengths in MM optimized geometries with different generations of OpenFF force fields are in close agreement with QM values. In particular, the mean of the bond RMSDs is 0.01 Å and the standard deviation is 0.004 Å. For angles, the mean of the angle RMSDs is 1.98° and the standard deviation is 0.50°.

**Figure 9:**
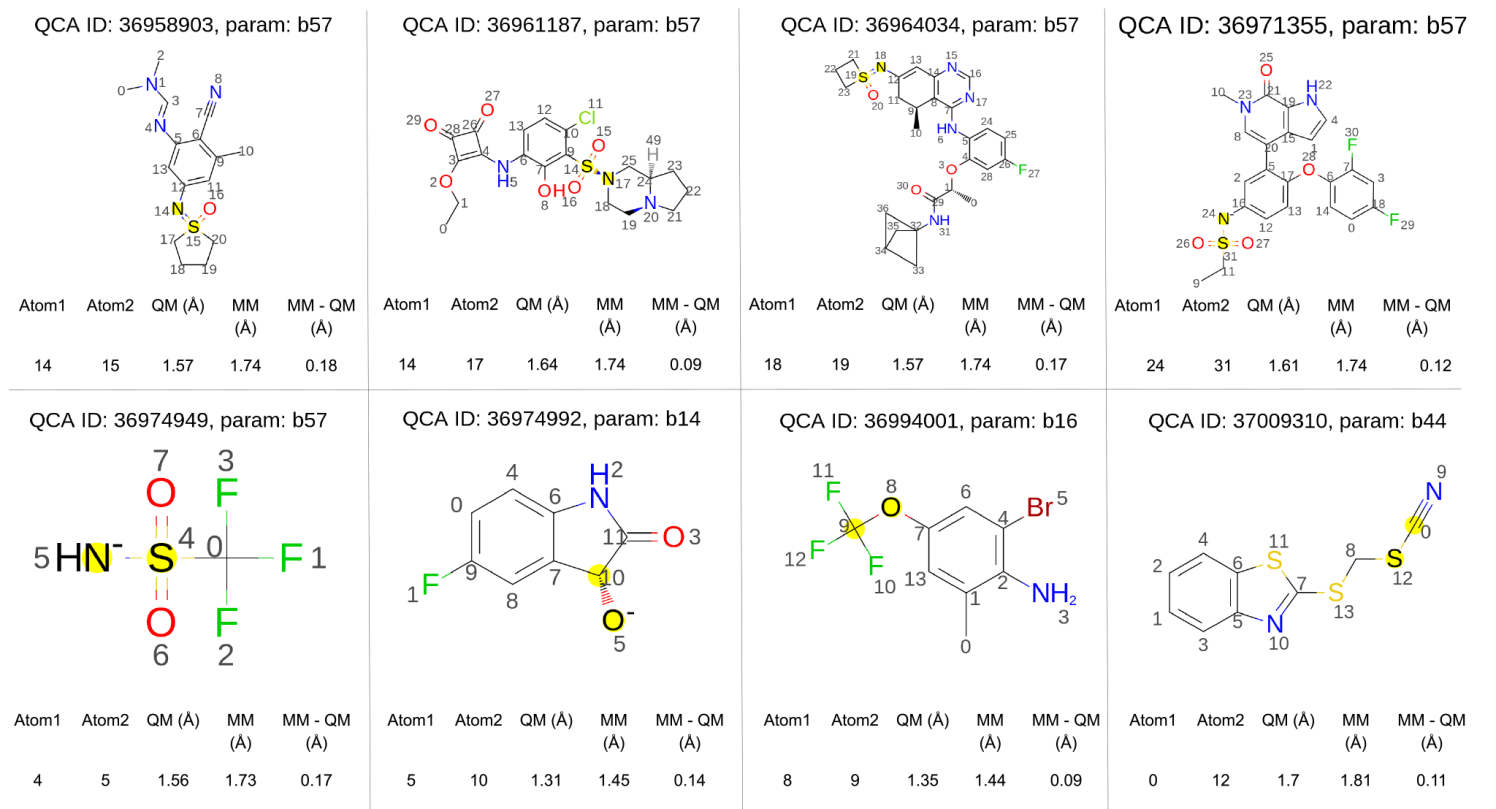
Representative molecules from the outliers in the bond-stretch RMSD box plot (Figure 8) using Sage 2.0.0. The major bond deviation with respect to the QM minimized structure is quantified below each molecule, and the bond parameter applied is given above each molecule, along with its QCArchive record id. Bond parameter b57, with the SMIRKS pattern [#16X4,#16X3:1]∼[#7:2], is a frequent offender.

Although the global averages of bond RMSDs are generally far lower than 0.05 Å, there are some outlier chemistries with slightly higher bond deviations of around 0.1 Å, and angles with larger deviations of 30° or more that may need additional force field refinement in the future. Representative molecules for larger bond deviations are shown in Figure 9. Most of them involve a nitrogen attached to a hypervalent sulfur, such as in imino-oxo-sulfanes and sulfonamides. Other cases of slightly higher bond deviations include a carbon in trifluoromethyl connected to an oxygen. The bond parameters assigned to these are b14 ([#6:1]-[#8:2]), b16 ([#6X4:1]–[#8X2H0:2]), b44 ([#16:1]–[#6:2]), and b57 ([#16X4,#16X3:1]∼[#7:2]). Among these outliers, b57, which applies to “sulfur∼nitrogen” bond, is a frequent offender, with deviations from QM structures as large as 0.18 Å.

**Figure 10:**
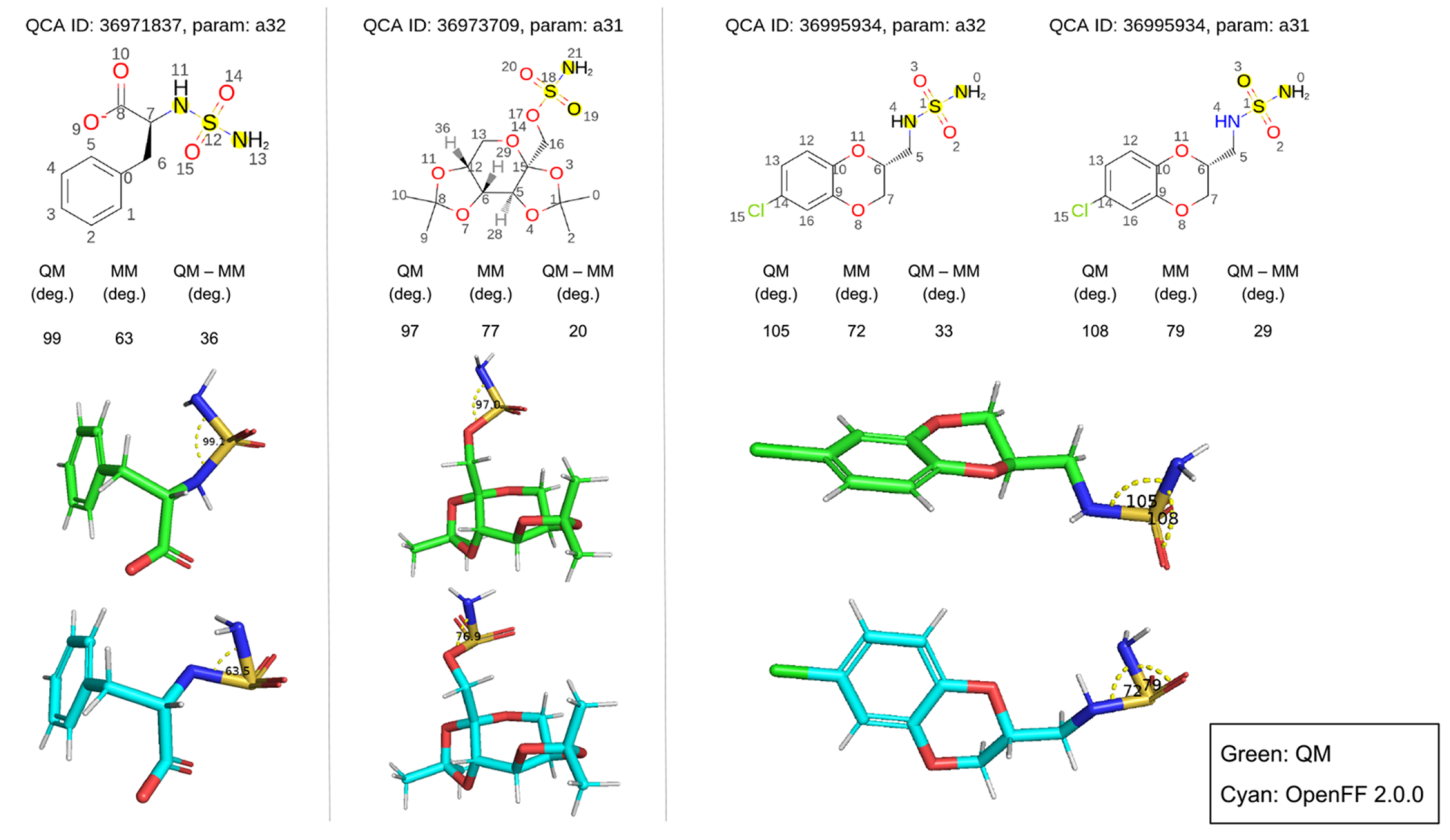
Representative molecules from the outliers in the bond-angle RMSD box plot for Sage 2.0.0. The major angle deviation with respect to the QM minimized structure is quantified below each molecule, and the angle parameter applied is given above the name of each molecule, along with its QCArchive record id. Angle parameters a31 and a32 (with the SMIRKS patterns [*:1]∼[#16X4:2]∼[*:3] and [*:1]–[#16X4,#16X3+0:2]–[*:3] respectively), for angles with hypervalent sulfur at their center are applied in these cases. The green colored structures are QM optimized geometries (reference) and the cyan colored structures are Sage 2.0.0 optimized geometries.

Outliers in angle RMSDs reveal a discrepancy with Sage 2.0.0 in describing a subset of sulfonamides that have a heteroatom neighbor. Angle parameters a31 ([*:1]∼[#16X4:2]∼[*:3]) and a32 ([*:1]-[#16X4,#16X3+0:2]-[*:3]), were applied to hypervalent sulfurs in these outliers. The [Heteroatom]∼S∼N angle (where Heteroatom is not the oxygen in a sulfonamide group) in MM optimized geometries shrunk to the range of 63°–79° in contrast to QM expected range of around 97°–105°, the deviations in MM with respect to the QM reference, were as large as 36°. The same parameters when applied to sulfonamides without a heteroatom neighbor closely reproduce the pyramidal angles of “C∼S∼N”. This issue is distinct from the sulfonamide discrepancy that corrected in Parsley 1.3.1 and again in Sage 2.0.0. The distorted structures with Sage 2.0.0 are shown in Figure 10. Other chemistries with larger angle deviations include highly flexible molecules, such as larger heterocycles that get optimized to a different ring pucker than the QM reference. These molecules with rapid interconversion of cycle conformations are a low priority to fix since the MM optimizer is free to choose one potential well over the other when there are almost equivalent minima.

#### 3.4.2 Torsion Deviations

**Figure 11:**
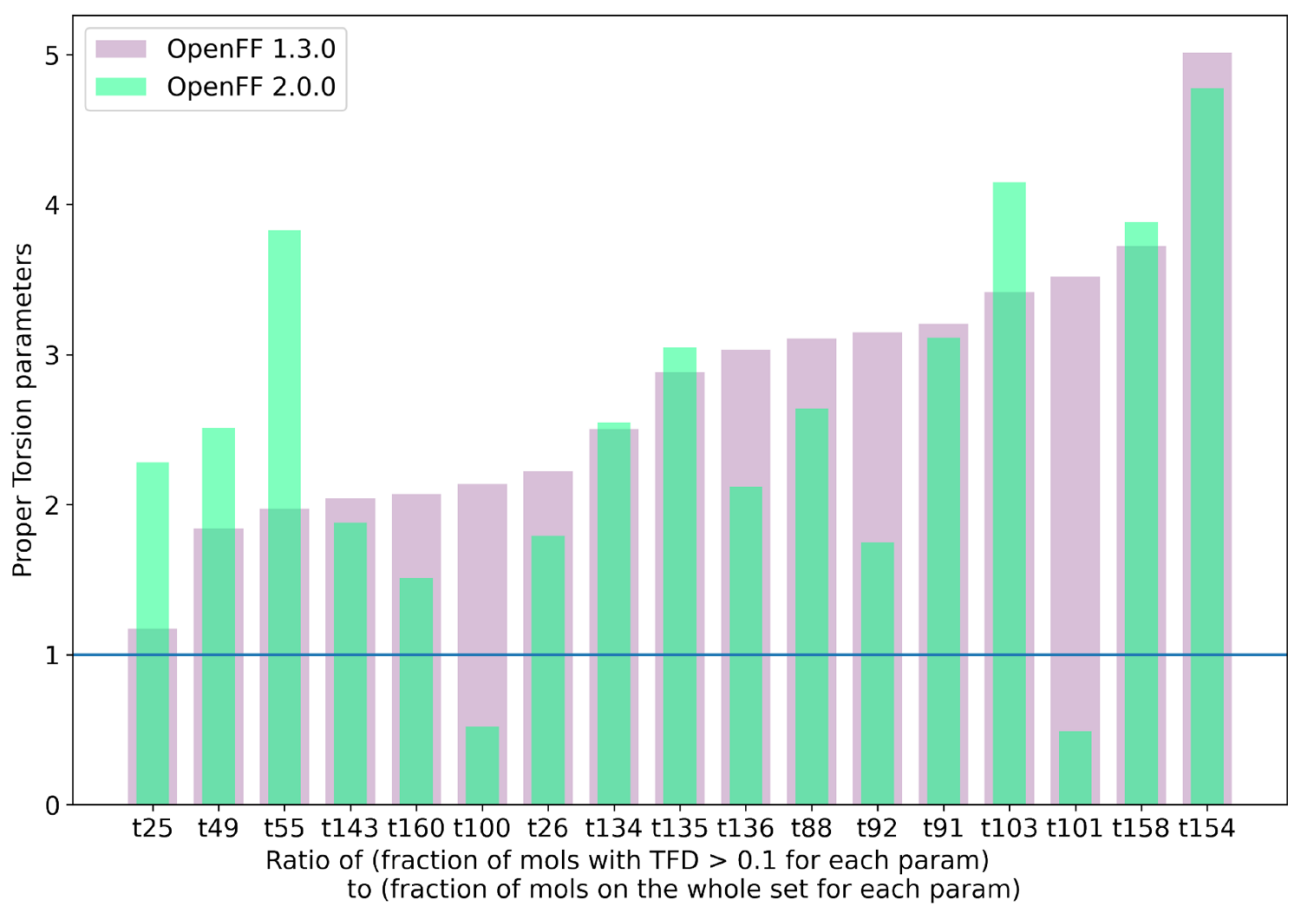
Over-representation of proper torsion parameters in molecules with TFD > 0.1, defined as the ratio of number of molecules with higher TFD where the torsion parameter is applied, to the number of molecules on the whole set where the same torsion parameter is applied. Parameters which are highly over-represented in molecules with larger TFD may be responsible for errors in their geometry. Parameters with over-representation values of 2 or higher are shown here. Parameters t25, t49, and t55, got slightly worse and the remaining got slightly better in Sage 2.0.0 compared to Parsley 1.3.0.

It is difficult to determine which torsion parameters need improvement from optimized geometries since any given optimized geometry is a result of all applied parameters and not simply a single torsional parameter. In an effort to determine which parameters might be particularly problematic, one helpful analysis is to tabulate the torsion parameters that are over-represented in the molecules that show a higher TFD when compared to the rest of the molecules. Figure 11 shows the distribution of torsion parameters in higher TFD cases when compared to the distribution of that same torsion parameter on the whole set of molecules under consideration. Torsion parameters t25, t49, t55, t88, t91, t103, t134, t135, t154, and t158 are the top ten parameters that are over-represented in cases with higher deviations, with the corresponding SMIRKS patterns tabulated in SI Table 4. t154 shows particularly high overrepresentation and is another case of a torsion involving hypervalent sulfur, with the SMIRKS pattern [*:1]∼[#16X4,#16X3+0:2]=,:[#7X2:3]-,:[*:4].

**Figure 12:**
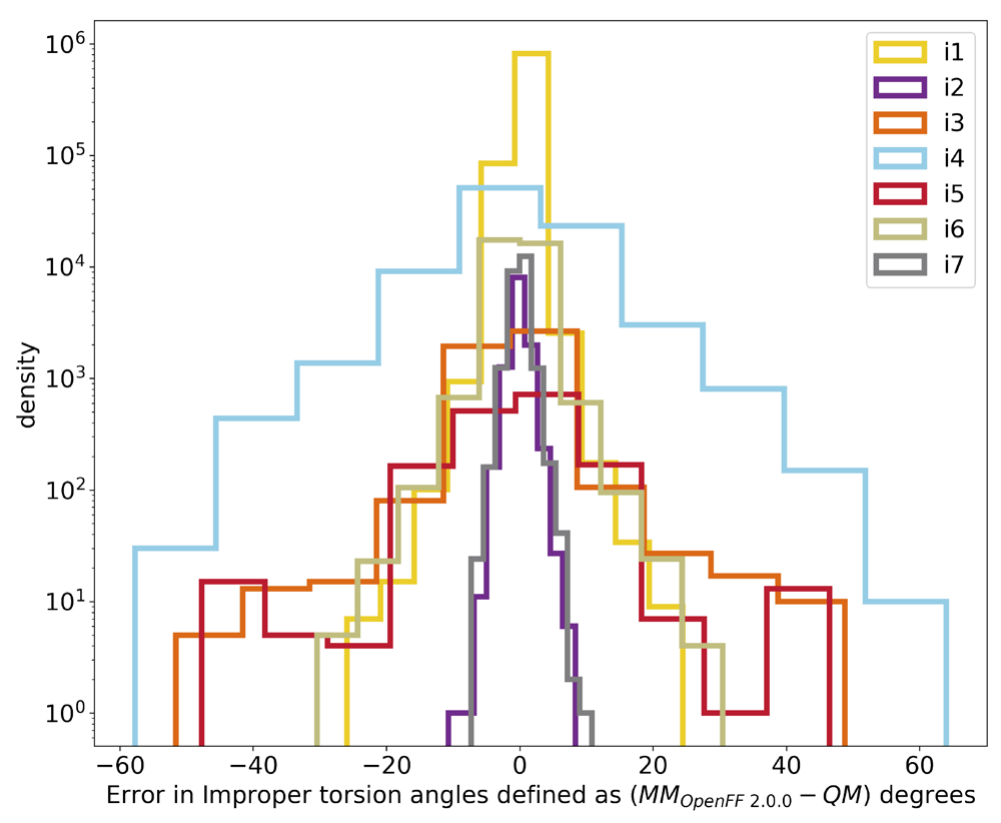
Deviations in improper angles of Sage 2.0.0 optimized geometries with respect to the reference QM structures for the whole benchmark set. The nitrogen centered improper i4 ([*:1]∼[#7X3(*∼[#6X3]):2](∼[*:3])∼[*:4]”) has a wider distribution as it covers both planar and pyramidal instances and can be improved by binning chemistries based on structural differences and splitting the current parameter to apply to subsets with distinct behaviors. This data suggests that, as we expect, there is substantial room to improve improper torsions in subsequent iterations of the force field.

Improper torsions need a special explanation as they were not reoptimized and therefore remain at their legacy (Parsley 1.3.0) values. Figure 12 shows the error distribution among the improper angle values when compared to QM structures on the whole benchmark set. Some of the deviations in improper angles around chiral atoms have a larger value when the out-of-plane atom in the MM optimized conformer is a mirror image of the QM conformation, but no filter was applied to weed out these cases, which thus may appear to be in substantial error even when they are not. Nitrogen-centered impropers, especially parameter i4 ([*:1]∼[#7X3(*∼[#6X3]):2](∼[*:3])∼[*:4]), have a wider distribution of improper angle disagreements. This particular parameter has a small force constant of 1 kcal/mol, which allows the geometry to range from planar (0°) to pyramidal (109.5°) in different chemical contexts. Thus, we see a wider distribution of deviations from −60 to +60 degrees in the improper angles where i4 is applied when compared to QM.

**Figure 13:**
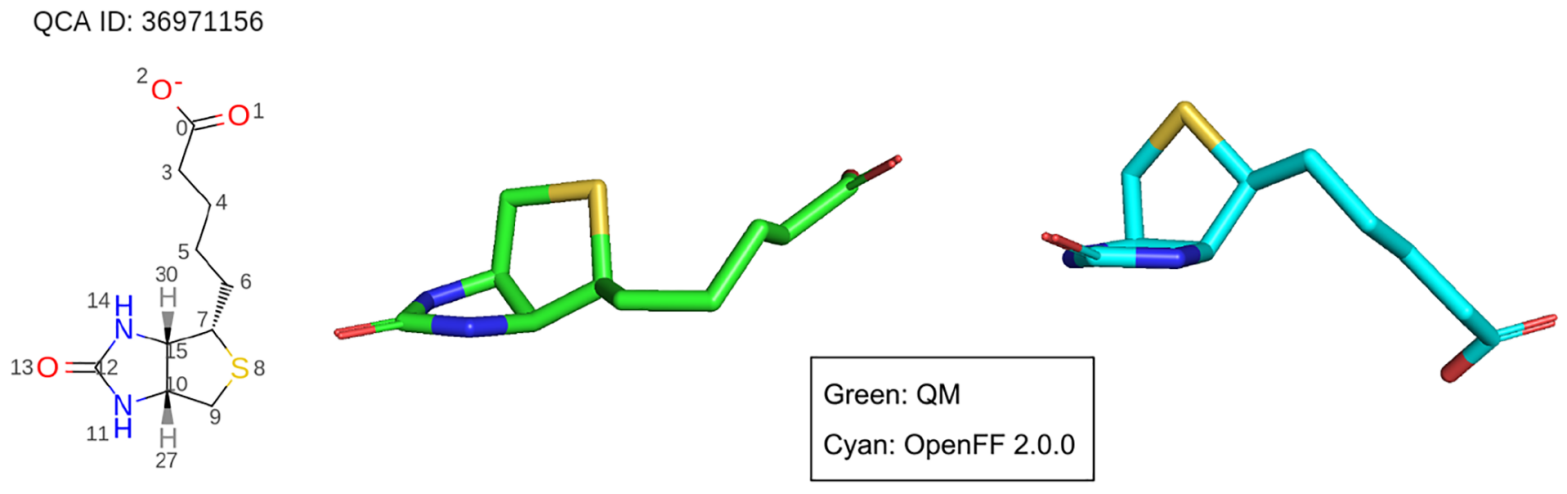
For the fused heterocycle, puckering in QM (green) is not reproduced well in the MM (cyan) optimized geometry, which remains flat. One of the bridgehead carbons (atom 10 in 2D depiction) can be seen to be out of plane in QM and almost in the plane of the ring with nitrogens for MM. We observe similar behavior in several other cases of bridgehead atoms with heteroatom neighbors.

Among other inaccuracies, puckering of small fused heterocycles is of concern where MM structures keep them flat while the QM expects them to be puckered. Incorrect descriptions of puckered rings as planar geometries would result in erroneous intramolecular and intermolecular nonbonded interactions, especially in hydrogen bonding interactions and π-stacked configurations. One such deviation is shown in Figure 13.

#### 3.4.3 How to Resolve Problems Affecting Specific Chemistries

To address such problematic chemistries, we must make specific changes in the process and then refit the entire force field. The refit can be done in a straightforward manner, but prior to this refitting, we must first do the following:

- Generate more QM training data, focusing on poorly described molecules as well as similar instances applicable to other chemistries. For example, to tackle the deficiency in sulfonamides we may need to create a data set of sulfonamides which have heteroatom neighbors for training and testing purposes. A similar procedure can be applied to other functional groups that are susceptible to strong changes in local chemical environment, such as conjugation, when heteroatom neighbors are present.
- Determine whether there is a need to split the bond, angle or torsion parameters in question into more specific SMIRKS patters, and whether to split either
  - based on geometry features of particular subsets and whether they are being described well or
  - based on the range of values each of the applied parameters would sample if we performed a custom fit of parameters on each of the subset molecules
- Fix any issues with missing periodicities in torsion parameters that are being applied on problematic molecules, as periodicities must be added manually rather than automatically during the fitting.

Such improvements will be incorporated into a subsequent force field releases after comprehensive reevaluation with results pass our overall benchmark metrics. One additional option may be to create unit tests for chemistries with systematic errors so that if subsequent force fields introduce failures on these chemistries, we will quickly detect such issues. We have created one such test set specific to simulations crashing during hydrogen mass repartitioning calculations, and this test has been used since Parsley 1.2.1.

### 3.5 Benchmarking: Protein–Ligand Benchmarking

We assessed the performance of the newly fitted Sage force field in relative binding free energy calculations. The Parsley results were compared to previously published results using the GAFF2.1x/AM1-BCC,^3^ and OPLS3e^9,113^ force fields. We specify the force field as “GAFF2.1x” as results across the data set are pulled from two different studies, with some systems using GAFF2.1^23^ and a later study using GAFF2.11.^24^ Specific systems using each force field version are listed in the SI in Table 5. The GAFF2.1x/AM1-BCC calculation results were calculated with the same *pmx* workflow for both Sage and Parsley, and the OPLS3e results were calculated with Schrödinger FEP+ as described in previous papers.^23,24^ The test set consisted of 22 different series of congeneric ligands binding to 20 protein targets with a total of 599 ligands.

**Figure 14:**
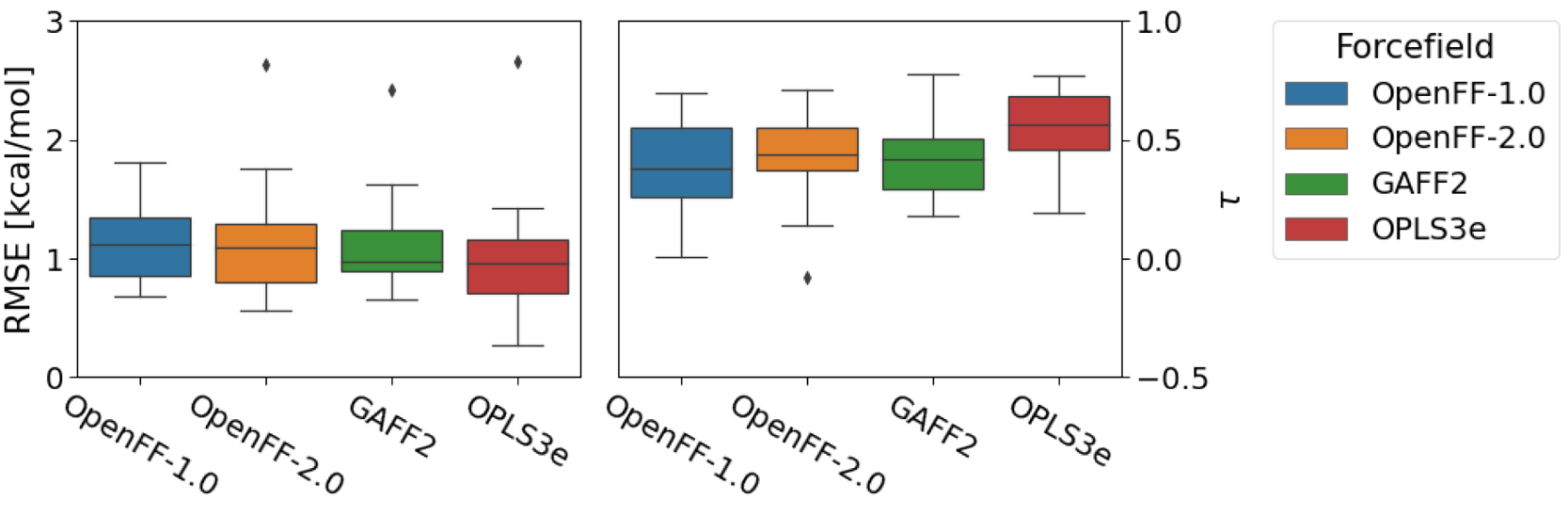
Results of protein–ligand binding free energy benchmarks. Each box represents the distribution across the individual RMSE (left) and Kendall’s τ (right) values of the different protein–ligand systems. In total, the results for each force field summarize binding free energies for 599 ligands in 22 congeneric series binding to 20 different protein targets. The metrics are based on the binding free energies Δ*G* calculated from relative ΔΔ*G* values (1133 values for each force field) with Arsenic (now called Cinnabar).^104^ The presented force fields are Parsley 1.0.0, Sage 2.0.0, GAFF2.1x/AM1-BCC, and OPLS3e (specific versions of GAFF2.1x given in SI for each system).

The Sage force field provides competitive accuracy in relative binding free energy calculations, as shown in Figure 14 where performance of the force fields is summarized according to absolute binding free energy differences back-calculated from the ΔΔ*G* estimates. Indeed, the accuracies of the four force fields examined here (Parsley 1.0.0, Sage 2.0.0, GAFF2.1x/AM1-BCC, and OPLS3e) are within 95% confidence intervals of each other. It is important to note that the accuracy of these calculations is strongly affected by additional factors, including input structure preparation and sampling time. In addition, protein force field, water model, and partial charge assignment in Sage (OpenFF 2.0.0) were identical to those used with Parsley 1.0.0. Given these considerations, we did not necessarily expect here that improvements in the small molecule force field would dramatically impact the accuracy of calculated binding free energies. In particular, our main goal was to ensure that the substantial refit of LJ parameters done in the present effort did not adversely affect accuracy of these calculations, while improving accuracy in more direct measures of force field quality. We also note that the accuracy for these calculations depends on the protein–ligand system, ranging from an RMSE = 0.57 kcal/mol for target galectin to RMSE = 2.62 kcal/mol for target PDE10 using the Sage 2.0.0 force field (SI Table 6).

With respect to the overall RMSE across all targets for binding free energy Δ*G*°, Sage (RMSE = 1.29 [1.17;1.33] kcal/mol) is statistically indistinguishable in performance from the other force fields tested (OPLS3e (RMSE = 1.18 [1.09;1.27] kcal/mol), GAFF2.1x (RMSE = 1.22 [1.13;1.32] kcal/mol), and Parsley (RMSE = 1.25 [1.20;1.33 kcal/mol)) except that with Sage, one target (PDE10), is a significant outlier. These results indicate that Sage is a reasonable choice of small molecule force field in combination with the TIP3P water model and the AMBER ff99sb*-ILDN force field for binding free energy calculations in drug discovery projects.

Details of the benchmark set and the RMSE and Kendall’s tau values against experimental protein–ligand affinity data for each force field tested are tabulated in SI Tables 5–7.

## 4 Conclusions

In this work, we report on the next generation the OpenFF family, OpenFF Sage 2.0.0. Sage builds on the valence parameter refits in the Parsley generation of force fields by including fitting to condensed-phase properties for updated LJ parameters while performing a further set of updates to the valence parameters. The Sage release also introduces a variety of improvements to the fitting procedures and data sets and incorporates our recent finding that fitting to condensed phase mixture properties can provide accuracy gains relative to fitting to pure solution properties alone.^50^

We also report extensive benchmarks of Sage on data from outside its training set. These show that Sage improves agreement with experiment, relative to Parsley 1.3.0, for calculations including solvation and transfer free energies, likely primarily as a result of the refitted LJ parameters. We also find improved agreement with QM molecular energies, likely due to improvements in the valence parametrization. Further, calculations of protein–ligand binding free energies provide confirmation that this first refit of LJ parameters does not adversely affect accuracy and that performance is comparable to other state-of-the-art small molecule force fields such as, GAFF 2.1/AM1-BCC and GAFF 2.11/AM1-BCC, OPLS3e, and CGenFF.

The force field parametrization presented here thus provides a strong platform for continued improvement. In the future, using carefully selected additional condensed phase mixtures in LJ parametrization should allow us to better capture biopolymer nonbonded interactions. We also anticipate that new methods for faster estimation of partial charges^114^ will enable co-optimization of LJ parameters and charges, further improving nonbonded interactions critical to accurate binding free energy calculations.

While our most substantial changes for Sage were the LJ parameter refits, updates to our valence parameter training data and fitting procedures also substantially changed the force field, even apart from the LJ refits. In particular, valence parameter optimization resulted in improvements in global metrics of quality for generated conformers—RMSD and TFD—on benchmarks relative to previous generations of force fields, and in the case of relative conformer energetics (ΔΔ*E*), a closer match to the previous best, which is Parsley 1.2.0. There has been significant refinement in the choice of training targets used in training our force fields since Parsley 1.0.0. These improvements have included capping the number of optimized conformers used as well as excluding vibrational frequency targets from training. For Sage, we also substantially expanded our quantum chemical benchmarking data set, giving us a better view into changes in performance. This larger benchmark set, “OpenFF Industry Benchmark Season 1 v1.1”, is an important first step in finding discrepancies in describing pharmaceutically relevant chemistries, although it does not currently cover all the valence parameters because some parameters are used only by rare chemistries that are uncommon in pharmaceutical data sets.

In addition to global benchmarking metrics, which can mask errors for niche chemistries, granular benchmarking in the form of parameter-wise or chemistry-wise analyses helped capture some such areas where there is room for further improvement. Analyzing such poor performers, we found that Sage 2.0.0 has a pathology in describing angles of sulfonamides with heteroatom neighbors, as well as slightly elongated bonds between sulfur and amide or sulfur and double-bonded nitrogens. Another issue is with puckering of small fused rings resulting in geometry deviations near bridgehead atoms; we also see an inability to capture the effects of pyridine lone pairs on condensed phase energetics. We plan to address these errors in subsequent refits in the near term.

Analyzing over-represented parameters in the outliers of TFD helps better understand errors in torsion fitting, since torsions are applied in diverse chemical contexts and sometimes a group of torsions can pass through the same central bond. Improper torsion parameters have also not yet been optimized, providing further opportunity to improve the force field in subsequent work, as some of the parameters describe improper angles with errors as high as 60° compared to QM. Whether improper torsion scans are needed or using current data in the form of proper torsion scans and optimized geometries will suffice is a question to be answered.

This work resulted in a number of key findings which will inform future work. In particular, lead-up work suggested that the choice of condensed phase property data used in training LJ parameters is particularly important, and we found that using mixture properties rather than pure solution properties and heat of vaporization seemed to improve performance.^50^ In Sage, this manifested by significantly improved performance on solvation free energies for both aqueous and nonaqueous solvents (Figure 5). We also observed that the choice of quantum chemical data set for valence parameters substantially impacted performance, and the data set expansions for Parsley 1.2.0 and 1.3.0 and Sage improved performance here substantially relative to Parsley 1.0.0. We also increased the size and diversity of our quantum chemical benchmarking data sets, allowing us both to better see changes in overall performance and to identify specific chemistries and parameters that need further refinement.

This study would have been virtually impossible without the open source OpenFF data and software infrastructure, which enabled rapid prototyping and rapid iteration through multiple fitting experiments. Additionally, the benchmarking infrastructure allowed us to identify systematic errors, and we plan to employ this going forward to help us fix critical problems even before releasing force fields. All of the data sets and infrastructure used here are openly and freely available under permissive licenses. We hope that this work and the open procedures described in this article facilitate further force field development cycles both within OpenFF and in the broader community. We anticipate considerable room for future fixed-charge force fields of the form employed here, but many other avenues for further work are open as well; for example, off-site charges are likely important in certain key chemical environments and should be explored,^113,115,116^ and polarizable force fields^117−119^ are also of considerable interest. We hope OpenFF infrastructure and data sets can provide a foundation for further exploration in these areas as well.

## Data Availability

All the data is made publicly available along with complete details on how to reproduce the training results at https://github.com/openforcefield/openff-sage. The released force fields can be accessed from https://github.com/openforcefield/openff-forcefields. General scripts for using the force field in molecular simulations can be found in openff-toolkit examples at https://github.com/openforcefield/openff-toolkit/tree/main/examples.

## Supporting Information Available

Details related to valence parameter training such as graphs depicting coverage of functional groups in valence training sets, general analysis on how the regularization scales were chosen in fitting, the drop in objective function during optimization, and the mapping of parameter ids to smarts patterns; metrics of physical property benchmarks, the root mean-squared errors; additional details on protein–ligand binding free energy calculations, the systems studied, and references to the structures used as inputs (PDF)
