# Supplementary material for: Development and Benchmarking of Open Force Field 2.0.0: The Sage Small Molecule Force Field
Source: J Chem Theory Comput. 2023 May 11;19(11):3251–75. doi: 10.1021/acs.jctc.3c00039 (PMC10269353; doi:10.1021/acs.jctc.3c00039)
Supplement: Supplementary file 1 — ct3c00039_si_001.pdf [file ct3c00039_si_001.pdf]

Supporting Information for "Development and Benchmarking of Open Force Field 2.0.0 — the Sage Small Molecule Force Field"

Simon Boothroyd[1], **Pavan Kumar Behara**[2], **Owen C. Madin**[3], **David F. Hahn**[4], **Hyesu Jang**[8,12], **Vytautas Gapsys**[4,5], **Jeffrey R. Wagner**[2,6], **Joshua T. Horton**[7], **David L. Dotson**[6,13], **Matthew W. Thompson**[3,6], **Jessica Maat**[9], **Trevor Gokey**[9], **Lee-Ping Wang**[8], **Daniel J. Cole**[7], **Michael K. Gilson**[10], **John D. Chodera**[11], **Christopher I. Bayly**[12], **Michael R. Shirts**[*3], **David L. Mobley**[*2,9]

[1]Boothroyd Scientific Consulting Ltd., London WC2H 9JQ, UK.; [2]Department of Pharmaceutical Sciences, University of California, Irvine, California 92697, USA; [3]Chemical & Biological Engineering Department, The University of Colorado at Boulder, Boulder, CO 80309, USA; [4]Computational Chemistry, Janssen Research & Development, Turnhoutseweg 30, Beerse B-2340, Belgium; [5]Computational Biomolecular Dynamics Group, Department of Theoretical and Computational Biophysics, Max Planck Institute for Multidisciplinary Sciences, Am Fassberg 11, D-37077, Göttingen, Germany; [6]The Open Force Field Initiative, Open Molecular Software Foundation, Davis, California 95616, USA; [7]School of Natural and Environmental Sciences, Newcastle University, Newcastle upon Tyne NE1 7RU, UK; [8]Chemistry Department, The University of California at Davis, Davis, California, 95616, USA; [9]Department of Chemistry, University of California, Irvine, California 92697, USA; [10]Skaggs School of Pharmacy and Pharmaceutical Sciences, The University of California at San Diego, La Jolla, California 92093, USA; [11]Computational & Systems Biology Program, Sloan Kettering Institute, Memorial Sloan Kettering Cancer Center, New York, New York 10065, USA; [12]OpenEye Scientific Software, Santa Fe, New Mexico 87508, USA; [13]Datryllic LLC, Phoenix, Arizona 85003, USA

***For correspondence:**
michael.shirts@colorado.edu (MRS); dmobley@uci.edu (DLM)

S1.1 Coverage of functional groups in datasets

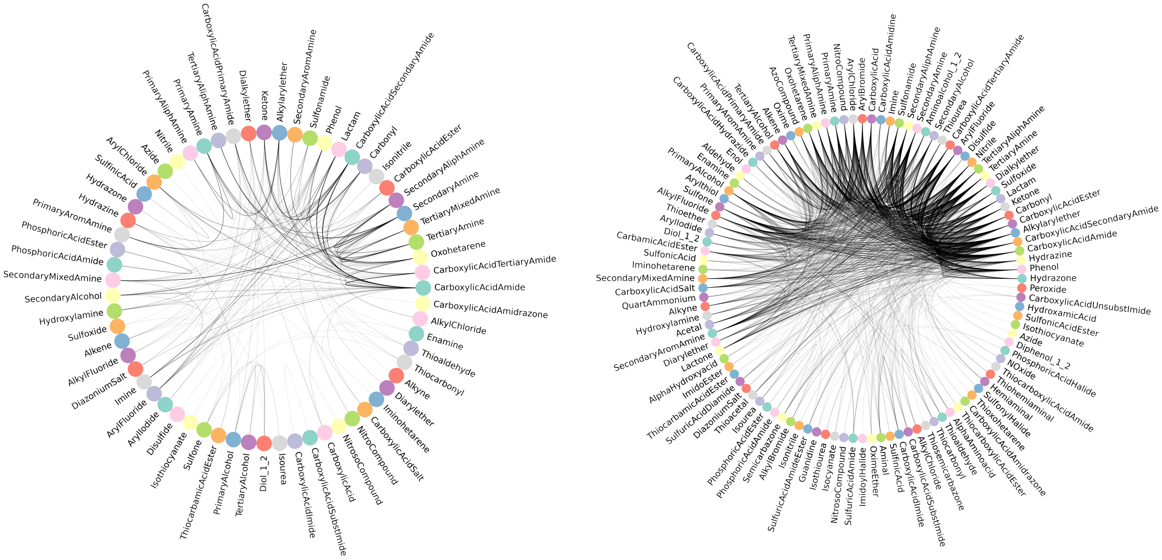

Figure S1. Combinations of different functional groups within molecules of the training datasets shown as network connectivity graphs. Functional groups are represented as nodes, and are connected by edges if both functional groups were present in the same molecule. There were 620 unique molecules in Generation 1 datasets covering 63 functional groups (nodes) with 739 edges. There were 1526 unique molecules in Generation 2 datasets and they covered around 108 functional groups (nodes) with 5533 edges. This increase in connectivity between different clusters of pharmaceutically relevant functional groups shows the substantial improvement in coverage as well as intermixing of chemistries in Generation 2 datasets over Generation 1 datasets. The functional groups representing each node and associated number of molecules in each of them are provided in SI Table S2.

S1.2 Performance on physical property fitting targets

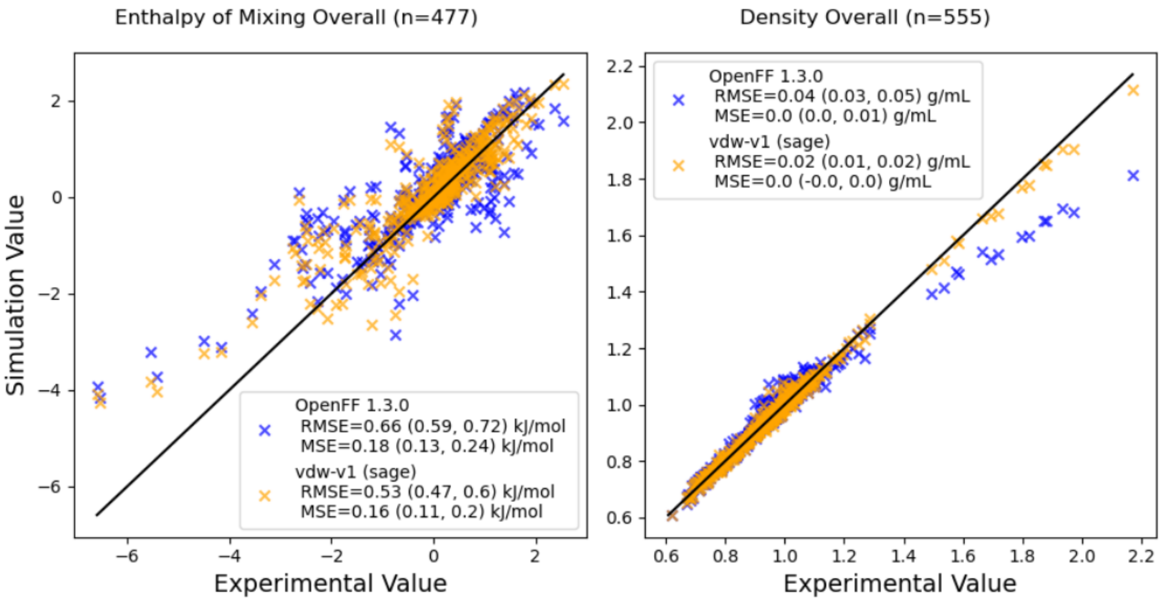

Figure S2. Parity plot between simulation and experiment for all data points in the vdw training set, shown before training (OpenFF 1.3.0) and after training (*vdw-v1*). Values in parentheses indicate bootstrapped 95% confidence intervals.

Force field	Performance against experiment, kcal/mol (95% CI)					
	ΔG_{solv}, aq		ΔG_{solv}, non-aq		$\Delta G_{trans}(aq \rightarrow nonaq)$	
	RMSE	MSE	RMSE	MSE	RMSE	MSE
OpenFF 1.3.0	**1.31** (1.10, 1.52)	**0.49** (0.21, 0.76)	**0.86** (0.75, 0.98)	**-0.005** (-0.09, 0.09)	**1.19** (1.11, 1.29)	**0.48** (0.36, 0.61)
vdw-v1	**1.02** (0.90, 1.13)	**0.42** (0.23, 0.61)	**0.77** (0.67, 0.89)	**-0.05** (-0.13, 0.04)	**1.00** (0.92, 1.09))	**0.46** (0.35, 0.55)
OpenFF 2.0.0	**1.09** (0.95, 1.23)	**0.44** (0.22, 0.68)	**0.78** (0.67, 0.91)	**-0.05** (-0.12, 0.04)	**1.08** (0.99, 1.17))	**0.49** (0.39, 0.60)
GAFF 2.11/AM1-BCC	**1.19** (1.00, 1.35)	**0.62** (0.39, 0.85)	**0.98** (0.87, 1.10)	**0.25** (0.15, 0.35)	**0.96** (0.88, 1.05)	**0.32** (0.22, 0.43)

Table S1. Root mean squared error (RMSE) and mean signed error (MSE) for aqueous and non-aqueous ΔG_{solv} and $\Delta G_{trans}(aq \rightarrow nonaq)$, for OpenFF 1.3.0, the intermediate force field *vdw-v1* (after vdw refitting and before valence refitting), the refit OpenFF 2.0.0 Sage, and GAFF 2.11/AM1-BCC. 95% confidence intervals were computed by parametric bootstrapping with replacement over molecules for 1000 iterations.

S1.3 Physical property benchmarking

S1.4 Distribution of heavy atoms in datasets

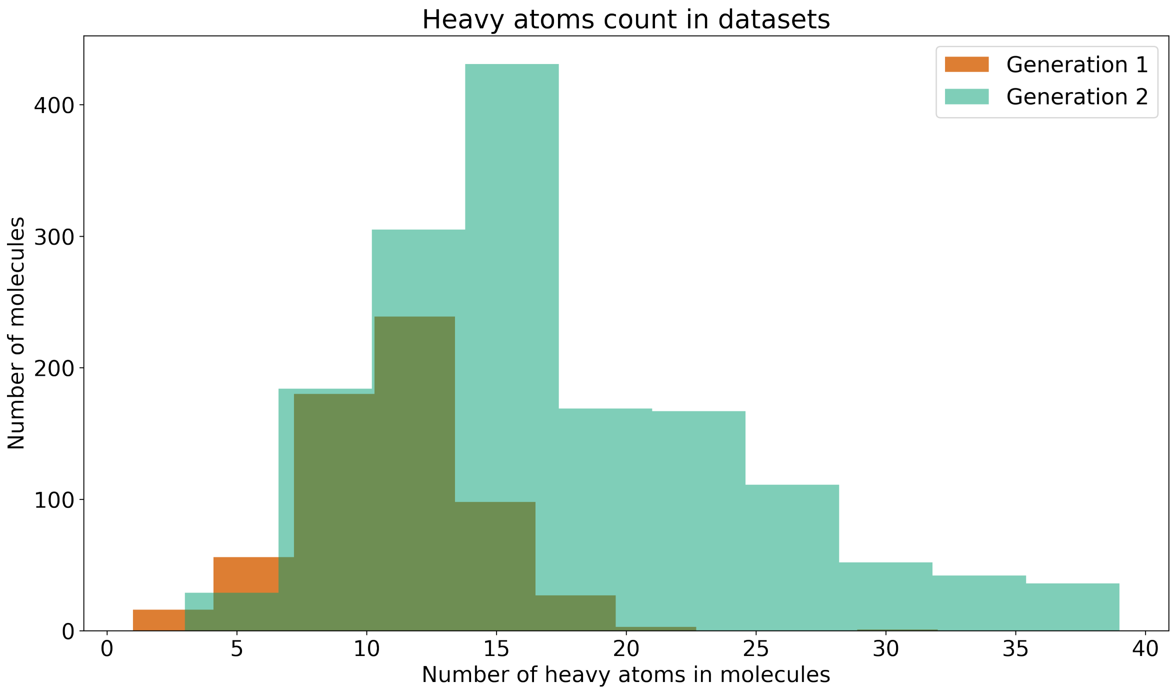

Figure S3. Distribution of number of heavy atoms among unique molecules in Generation 1 and 2 datasets. Generation 2 training datasets show inclusion of additional large molecules (with >20 heavy atoms) that have more rotatable bonds, leading to larger numbers of structurally diverse stable conformations, as well as complex intramolecular nonbonded interactions from diverse chemistries.

S1.5 Regularization scales

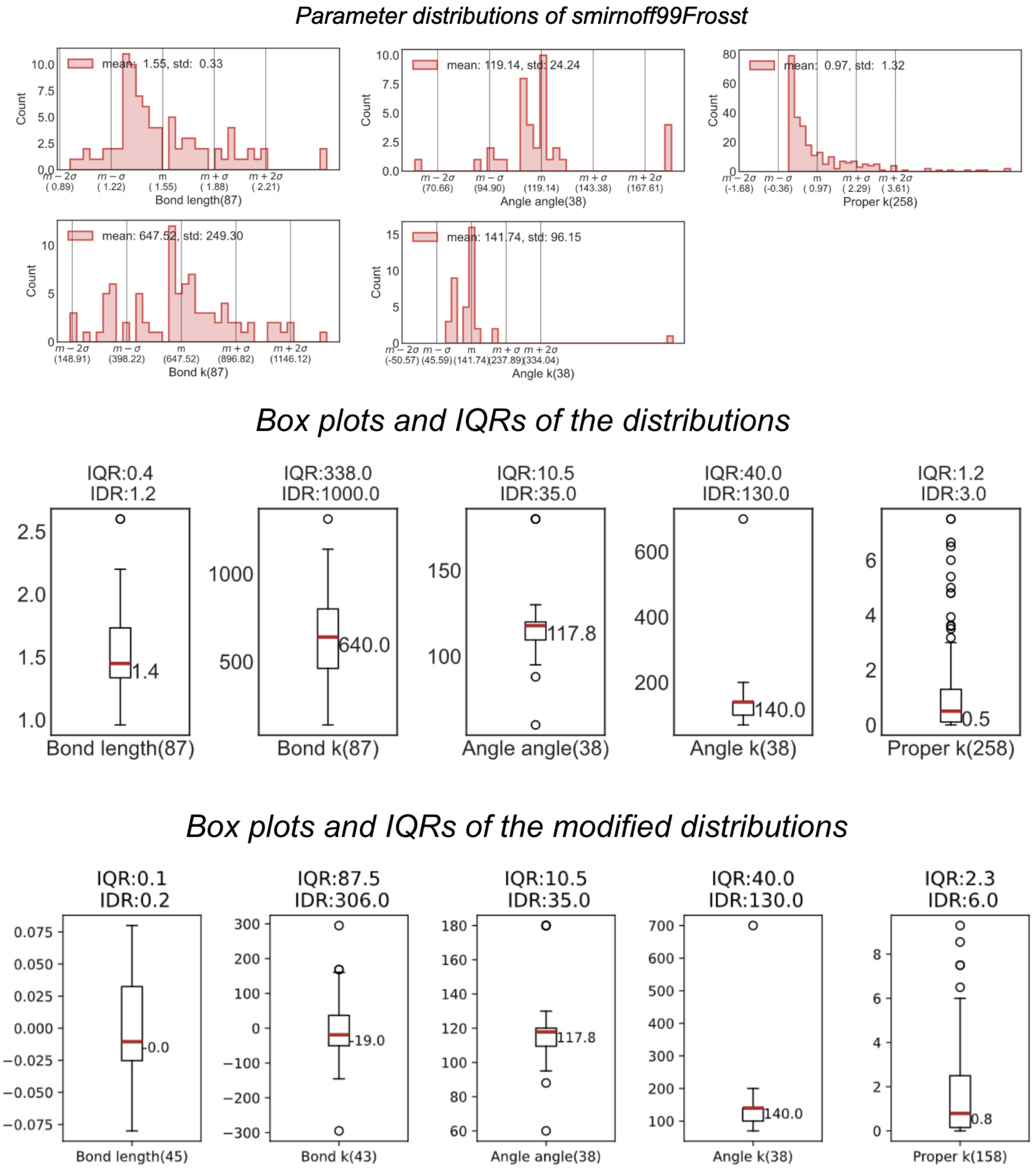

Figure S4. (a). Distributions of values of regularization scales σ_p in SMIRNOFF99Frosst for each parameter type bond length (in units of Å), bond force constant (in kcal/mol/Å^2), angle value (in °), angle force constant (in kcal/mol/radian^2), torsion force constants (in kcal/mol). (b). IQR (interquartile range) values of the distribution of parameter values in SMIRNOFF99Frosst, shown in **a**. The IQR values were higher for bond and torsion parameters due to mixing of parameter values that were on different scales in different chemical contexts. (c). IQR values of the modified distributions of parameter values, which exclude dependencies on bond orders and element compositions. The reduced number of bond lengths and bond force constants could be observed as some of the subsets were grouped together. The IQR values were on the same order as the regularization scales used in fitting Parsley.

S1.6 Functional groups in generation 1 and 2 datasets

Table S2. In the generation 1 datasets, the number of unique molecules is 620, with 63 nodes and 739 edges. In Generation 2 datasets number of unique molecules is 1526, with 108 nodes and 5533 edges. Listed below are the functional groups as categorized by Checkmol,[1] used via the `nonbonded` package,[2] and the number of molecules per functional group.

Functional group	No. of occurrences in Gen 1	No. of occurrences in Gen 2
ThiocarboxylicAcidEster	0	3
CarboxylicAcid	5	153
SulfonylHalide	0	2
CarbamicAcidEster	0	78
TertiaryAmine	60	543
CarboxylicAcidPrimaryAmide	31	30
Aminal	0	17
ImidoEster	0	4
Hydrazone	3	97
QuartAmmonium	0	11
ImidoylHalide	0	1
Lactam	62	342
Sulfone	7	193
Diphenol_1_2	0	3
SecondaryMixedAmine	34	106
SecondaryAmine	103	475
Hydrazine	12	146
TertiaryMixedAmine	37	196
Hemiaminal	0	8
Thiourea	0	44
Oxime	0	10
Ketone	45	284
PrimaryAmine	40	243
Iminohetarene	1	12
CarboxylicAcidSubstImide	1	12
Lactone	0	75
CarboxylicAcidAmide	244	798
Oxohetarene	56	241
CarboxylicAcidSecondaryAmide	145	384
Imine	24	45
Thioaldehyde	4	11
Hydroxylamine	14	23
Guanidine	0	3
SulfonicAcid	0	10
ArylIodide	4	30
SecondaryAlcohol	27	149
SecondaryAliphAmine	59	278
Acetal	0	47
PrimaryAliphAmine	16	185
TertiaryAlcohol	5	114
Thioacetal	0	20
PrimaryAromAmine	24	58
Aldehyde	0	14

continues on next page

… continued

Functional group	No. of occurrences in Gen 1	No. of occurrences in Gen 2
CarboxylicAcidEster	11	225
SulfuricAcidDiamide	0	12
AlphaAminoacid	0	10
SulfonicAcidEster	0	1
Diarylether	2	37
Arylthiol	0	17
PhosphoricAcidAmide	8	5
SulfuricAcidAmide	0	12
DiazoniumSalt	6	4
Isothiourea	0	4
Isourea	2	9
Aminoalcohol_1_2	0	73
ArylBromide	0	223
PhosphoricAcidEster	8	3
SulfinicAcid	3	3
HydroxamicAcid	0	1
Nitrile	1	225
ThiocarboxylicAcidAmide	0	2
NOxide	0	3
Azide	1	1
OximeEther	0	8
Diol_1_2	8	22
CarboxylicAcidImide	1	17
Enol	0	2
Sulfoxide	2	69
Isocyanate	0	4
SecondaryAromAmine	10	120
CarboxylicAcidAmidine	0	26
PhosphoricAcidHalide	0	2
CarboxylicAcidTertiaryAmide	68	421
PrimaryAlcohol	9	152
AlkylChloride	4	8
Thiocarbonyl	4	11
CarboxylicAcidAmidrazone	1	2
Enamine	2	17
Disulfide	1	9
NitrosoCompound	1	2
Dialkylether	18	276
Thiosemicarbazone	0	1
CarboxylicAcidHydrazide	0	14
Carbonyl	50	312
Alkyne	1	33
Isonitrile	2	2
ArylFluoride	25	380
Thioether	0	101
Semicarbazone	0	16
NitroCompound	3	87

continues on next page

… continued

Functional group	No. of occurrences in Gen 1	No. of occurrences in Gen 2
CarboxylicAcidUnsubstImide	0	5
Isothiocyanate	1	1
ArylChloride	21	431
Alkene	7	178
AlkylFluoride	5	158
Alkylarylether	51	736
Thiohemiaminal	0	4
ThiocarbamicAcidEster	3	2
SulfuricAcidAmideEster	0	11
AlphaHydroxyacid	0	6
AzoCompound	0	56
Thioxohetarene	0	1
TertiaryAliphAmine	23	422
Sulfonamide	13	241
CarboxylicAcidSalt	4	66
Peroxide	0	2
AlkylBromide	0	8
Phenol	35	256

S1.7 Change in objective function during valence parameter optimization

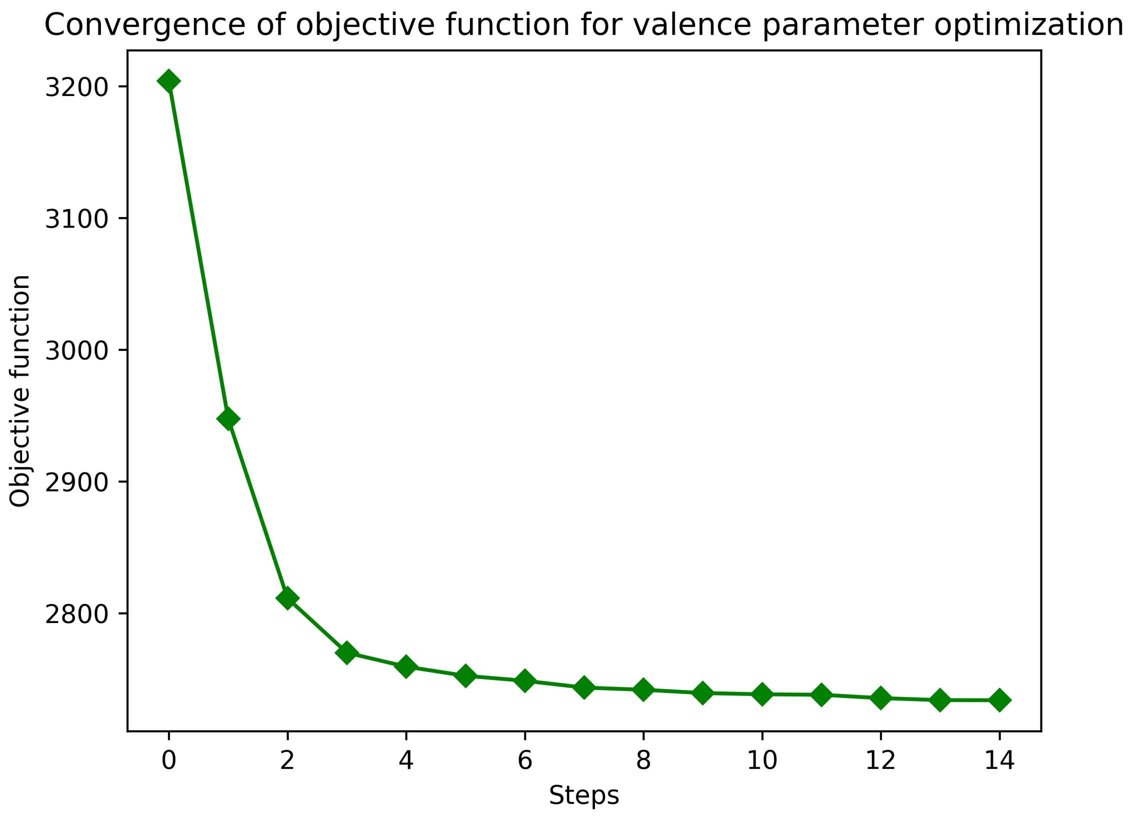

Figure S5. Change in objective function value during valence parameter optimization using ForceBalance.

S1.8 Parameters to SMARTS mapping

Table S3. Parameter ids and corresponding SMARTS patterns in the force field Sage 2.0.0. Bond parameters are labeled as ‘b#’, angle parameters as ‘a#’, proper torsion parameters as ‘t#’, and improper torsion parameters as ‘i#’.

Parameter ID	SMARTS Pattern
b1	[#6X4:1]-[#6X4:2]
b2	[#6X4:1]-[#6X3:2]
b3	[#6X4:1]-[#6X3:2]=[#8X1+0]
b4	[#6X3:1]-[#6X3:2]
b5	[#6X3:1]:[#6X3:2]
b6	[#6X3:1]=[#6X3:2]
b7	[#6:1]-[#7:2]
b8	[#6X3:1]-[#7X3:2]
b9	[#6X4:1]-[#7X3:2]-[#6X3]=[#8X1+0]
b10	[#6X3:1](=[#8X1+0])-[#7X3:2]
b11	[#6X3:1]-[#7X2:2]
b12	[#6X3:1]:[#7X2,#7X3+1:2]
b13	[#6X3:1]=[#7X2,#7X3+1:2]
b14	[#6:1]-[#8:2]
b15	[#6X3:1]-[#8X1-1:2]
b16	[#6X4:1]-[#8X2H0:2]
b17	[#6X3:1]-[#8X2:2]
b18	[#6X3:1]-[#8X2H1:2]
b19	[#6X3a:1]-[#8X2H0:2]
b20	[#6X3:1](=[#8X1])-[#8X2H0:2]
b21	[#6:1]=[#8X1+0,#8X2+1:2]
b22	[#6X3:1](~[#8X1])~[#8X1:2]
b23	[#6X3:1]~[#8X2+1:2]~[#6X3]
b24	[#6X2:1]-[#6:2]
b25	[#6X2:1]-[#6X4:2]
b26	[#6X2:1]=[#6X3:2]
b27	[#6:1]#[#7:2]
b28	[#6X2:1]#[#6X2:2]
b29	[#6X2:1]-[#8X2:2]
b30	[#6X2:1]-[#7:2]
b31	[#6X2:1]=[#7:2]
b32	[#16:1]=[#6:2]
b33	[#6X2:1]=[#16:2]
b34	[#7:1]-[#7:2]
b35	[#7X3:1]-[#7X2:2]
b36	[#7X2:1]-[#7X2:2]
b37	[#7:1]:[#7:2]
b38	[#7:1]=[#7:2]
b39	[#7+1:1]=[#7-1:2]
b40	[#7:1]#[#7:2]
b41	[#7:1]-[#8X2:2]
b42	[#7:1]~[#8X1:2]
b43	[#8X2:1]-[#8X2:2]

to be continued…

Table S3. Parameter ids and corresponding SMARTS patterns in the force field Sage 2.0.0. Bond parameters are labeled as 'b#', angle parameters as 'a#', proper torsion parameters as 't#', and improper torsion parameters as 'i#'. (continued)

… continued

Parameter ID	SMARTS Pattern
b44	[#16:1]-[#6:2]
b45	[#16:1]-[#1:2]
b46	[#16:1]-[#16:2]
b47	[#16:1]-[#9:2]
b48	[#16:1]-[#17:2]
b49	[#16:1]-[#35:2]
b50	[#16:1]-[#53:2]
b51	[#16X2,#16X1-1,#16X3+1:1]-[#6X4:2]
b52	[#16X2,#16X1-1,#16X3+1:1]-[#6X3:2]
b53	[#16X2:1]-[#7:2]
b54	[#16X2:1]-[#8X2:2]
b55	[#16X2:1]=[#8X1,#7X2:2]
b56	[#16X4,#16X3!+1:1]-[#6:2]
b57	[#16X4,#16X3:1]~[#7:2]
b58	[#16X4,#16X3:1]-[#8X2:2]
b59	[#16X4,#16X3:1]~[#8X1:2]
b60	[#15:1]-[#1:2]
b61	[#15:1]~[#6:2]
b62	[#15:1]-[#7:2]
b63	[#15:1]=[#7:2]
b64	[#15:1]~[#8X2:2]
b65	[#15:1]~[#8X1:2]
b66	[#16:1]-[#15:2]
b67	[#15:1]=[#16X1:2]
b68	[#6:1]-[#9:2]
b69	[#6X4:1]-[#9:2]
b70	[#6:1]-[#17:2]
b71	[#6X4:1]-[#17:2]
b72	[#6:1]-[#35:2]
b73	[#6X4:1]-[#35:2]
b74	[#6:1]-[#53:2]
b75	[#6X4:1]-[#53:2]
b76	[#7:1]-[#9:2]
b77	[#7:1]-[#17:2]
b78	[#7:1]-[#35:2]
b79	[#7:1]-[#53:2]
b80	[#15:1]-[#9:2]
b81	[#15:1]-[#17:2]
b82	[#15:1]-[#35:2]
b83	[#15:1]-[#53:2]
b84	[#6X4:1]-[#1:2]
b85	[#6X3:1]-[#1:2]
b86	[#6X2:1]-[#1:2]
b87	[#7:1]-[#1:2]

to be continued…

Table S3. Parameter ids and corresponding SMARTS patterns in the force field Sage 2.0.0. Bond parameters are labeled as 'b#', angle parameters as 'a#', proper torsion parameters as 't#', and improper torsion parameters as 'i#'. (continued)

… continued

Parameter ID	SMARTS Pattern
b88	[#8:1]-[#1:2]
a1	[*:1]~[#6X4:2]-[*:3]
a2	[#1:1]-[#6X4:2]-[#1:3]
a3	[*;r3:1]1~;@[*;r3:2]~;@[*;r3:3]1
a4	[*;r3:1]~;@[*;r3:2]~;!@[*:3]
a5	[*:1]~;!@[*;r3:2]~;!@[*:3]
a6	[#1:1]-[*;r3:2]~;!@[*:3]
a7	[#6r4:1]-;@[#6r4:2]-;@[#6r4:3]
a8	[!#1:1]-[#6r4:2]-;!@[!#1:3]
a9	[!#1:1]-[#6r4:2]-;!@[#1:3]
a10	[*:1]~[#6X3:2]~[*:3]
a11	[#1:1]-[#6X3:2]~[*:3]
a12	[#1:1]-[#6X3:2]-[#1:3]
a13	[*;r6:1]~;@[*;r5:2]~;@[*;r5;x2:3]
a14	[*:1]~;!@[*;X3;r5:2]~;@[*;r5:3]
a15	[#8X1:1]~[#6X3:2]~[#8:3]
a16	[*:1]~[#6X2:2]~[*:3]
a17	[*:1]~[#7X2:2]~[*:3]
a18	[*:1]-[#7X4,#7X3,#7X2-1:2]-[*:3]
a19	[#1:1]-[#7X4,#7X3,#7X2-1:2]-[*:3]
a20	[*:1]~[#7X3$(*~[#6X3,#6X2,#7X2+0]):2]~[*:3]
a21	[#1:1]-[#7X3$(*~[#6X3,#6X2,#7X2+0]):2]-[*:3]
a22	[*:1]~[#7X2+0:2]~[*:3]
a23	[*:1]~[#7X2+0:2]~[#6X2:3](~[#16X1])
a24	[#1:1]-[#7X2+0:2]~[*:3]
a25	[#6,#7,#8:1]-[#7X3:2](~[#8X1])~[#8X1:3]
a26	[#8X1:1]~[#7X3:2]~[#8X1:3]
a27	[*:1]~[#7X2:2]~[#7X1:3]
a28	[*:1]-[#8:2]-[*:3]
a29	[#6X3,#7:1]~;@[#8;r:2]~;@[#6X3,#7:3]
a30	[*:1]-[#8X2+1:2]=[*:3]
a31	[*:1]~[#16X4:2]~[*:3]
a32	[*:1]-[#16X4,#16X3+0:2]-[*:3]
a33	[*:1]~[#16X3$(*~[#8X1,#7X2]):2]~[*:3]
a34	[*:1]~[#16X2,#16X3+1:2]~[*:3]
a35	[*:1]=[#16X2:2]=[*:3]
a36	[*:1]=[#16X2:2]=[#8:3]
a37	[#6X3:1]-[#16X2:2]-[#6X3:3]
a38	[#6X3:1]-[#16X2:2]-[#6X4:3]
a39	[#6X3:1]-[#16X2:2]-[#1:3]
a40	[*:1]~[#15:2]~[*:3]
t1	[*:1]-[#6X4:2]-[#6X4:3]-[*:4]
t2	[#6X4:1]-[#6X4:2]-[#6X4:3]-[#6X4:4]
t3	[#1:1]-[#6X4:2]-[#6X4:3]-[#1:4]

to be continued…

Table S3. Parameter ids and corresponding SMARTS patterns in the force field Sage 2.0.0. Bond parameters are labeled as 'b#', angle parameters as 'a#', proper torsion parameters as 't#', and improper torsion parameters as 'i#'. (continued)

... continued

Parameter ID	SMARTS Pattern
t4	[#1:1]-[#6X4:2]-[#6X4:3]-[#6X4:4]
t5	[#8X2:1]-[#6X4:2]-[#6X4:3]-[#8X2:4]
t6	[#9:1]-[#6X4:2]-[#6X4:3]-[#9:4]
t7	[#17:1]-[#6X4:2]-[#6X4:3]-[#17:4]
t8	[#35:1]-[#6X4:2]-[#6X4:3]-[#35:4]
t9	[#1:1]-[#6X4:2]-[#6X4:3]-[#8X2:4]
t10	[#1:1]-[#6X4:2]-[#6X4:3]-[#9:4]
t11	[#1:1]-[#6X4:2]-[#6X4:3]-[#17:4]
t12	[#1:1]-[#6X4:2]-[#6X4:3]-[#35:4]
t13	[*:1]-[#6X4:2]-[#6X4;r3:3]-[*:4]
t14	[*:1]-[#6X4:2]-[#6X4;r3:3]-[#6X4;r3:4]
t15	[*:1]-[#6X4;r3:2]-@[#6X4;r3:3]-[*:4]
t16	[#6X4;r3:1]-[#6X4;r3:2]-[#6X4;r3:3]-[*:4]
t17	[*:1]~[#6X3:2]-[#6X4:3]-[*:4]
t18	[*:1]-[#6X4:2]-[#6X3:3]=[*:4]
t19	[#1:1]-[#6X4:2]-[#6X3:3]=[#8X1:4]
t20	[#1:1]-[#6X4:2]-[#6X3:3]=[#6X3:4]
t21	[#6X3:1]-[#6X4:2]-[#6X3:3]=[#6X3:4]
t22	[#7X3:1]-[#6X4:2]-[#6X3:3]-[#7X3:4]
t23	[#6X4:1]-[#6X4:2]-[#6X3:3]-[#7X3:4]
t24	[#16X2,#16X1-1,#16X3+1:1]-[#6X3:2]-[#6X4:3]-[#1:4]
t25	[#16X2,#16X1-1,#16X3+1:1]-[#6X3:2]-[#6X4:3]-[#7X4,#7X3:4]
t26	[#16X2,#16X1-1,#16X3+1:1]-[#6X3:2]-[#6X4:3]-[#7X3$(*-[#6X3,#6X2]):4]
t27	[*:1]-[#6X4;r3:2]-[#6X3:3]~[*:4]
t28	[#6X4:1]-[#6X4;r3:2]-[#6X3:3]~[#6X3:4]
t29	[#1:1]-[#6X4;r3:2]-[#6X3:3]~[#6X3:4]
t30	[#6X3:1]-[#6X4;r3:2]-[#6X3:3]-[#7X3:4]
t31	[#6X3:1]-[#6X4;r3:2]-[#6X3:3]=[#8X1:4]
t32	[#6X3:1]-[#6X4;r3:2]-[#6X3:3]~[#6X3:4]
t33	[#7X3:1]-[#6X4;r3:2]-[#6X3:3]~[#6X3:4]
t34	[#6X4;r3:1]-;@[#6X4;r3:2]-[#6X3:3]~[#6X3:4]
t35	[#6X4;r3:1]-;@[#6X4;r3:2]-[#6X3;r6:3]:[#6X3;r6:4]
t36	[#6X4;r3:1]-;@[#6X4;r3:2]-[#6X3;r5:3]-;@[#6X3;r5:4]
t37	[#6X4;r3:1]-;@[#6X4;r3:2]-[#6X3;r5:3]=;@[#6X3;r5:4]
t38	[#6X4;r3:1]-;@[#6X4;r3:2]-[#6X3:3]-[#6X4:4]
t39	[#6X4;r3:1]-;@[#6X4;r3:2]-[#6X3;r6:3]:[#7X2;r6:4]
t40	[#6X4;r3:1]-;@[#6X4;r3:2]-[#6X3:3]=[#7X2:4]
t41	[#6X4;r3:1]-;@[#6X4;r3:2]-[#6X3:3]-[#8X2:4]
t42	[#6X4;r3:1]-;@[#6X4;r3:2]-[#6X3:3]=[#8X1:4]
t43	[*:1]~[#6X3:2]-[#6X3:3]~[*:4]
t44	[*:1]~[#6X3:2]:[#6X3:3]~[*:4]
t45	[*:1]-,:[#6X3:2]=[#6X3:3]-,:[*:4]
t46	[#6X4:1]-[#6X3:2]=[#6X3:3]-[#6X4:4]
t47	[*:1]~[#6X3:2]-[#6X3$(*=[#8,#16,#7]):3]~[*:4]

to be continued...

Table S3. Parameter ids and corresponding SMARTS patterns in the force field Sage 2.0.0. Bond parameters are labeled as 'b#', angle parameters as 'a#', proper torsion parameters as 't#', and improper torsion parameters as 'i#'. (continued)

... continued

Parameter ID	SMARTS Pattern
t48	[#6X3:1]=[#6X3:2]-[#6X3:3]=[#8X1:4]
t49	[*:1]~[#7a:2]:[#6a:3]~[*:4]
t50	[*:1]-[#6X4:2]-[#7X4:3]-[*:4]
t51	[*:1]-[#6X4:2]-[#7X3:3]-[*:4]
t52	[*:1]-[#6X4:2]-[#7X3:3]-[#7X2:4]=[#6]
t53	[#1:1]-[#6X4:2]-[#7X3:3]-[#7X2:4]=[#6]
t54	[*:1]-[#6X4:2]-[#7X3:3]-[#7X2:4]=[#7X2,#8X1]
t55	[#1:1]-[#6X4:2]-[#7X3:3]-[#7X2:4]=[#7X2,#8X1]
t56	[*:1]-[#6X4:2]-[#7X3$(*@1-[*]=,:[*][*]=,:[*]@1):3]-[*:4]
t57	[#1:1]-[#6X4:2]-[#7X3$(*@1-[*]=,:[*][*]=,:[*]@1):3]-[*:4]
t58	[#6X4:1]-[#6X4:2]-[#7X4,#7X3:3]-[#6X4:4]
t59	[#1:1]-[#7X4,#7X3:2]-[#6X4;r3:3]-[*:4]
t60	[#1:1]-[#7X4,#7X3:2]-[#6X4;r3:3]-[#6X4;r3:4]
t61	[!#1:1]-[#7X4,#7X3:2]-[#6X4;r3:3]-[*:4]
t62	[!#1:1]-[#7X4,#7X3:2]-[#6X4;r3:3]-[#6X4;r3:4]
t63	[*:1]-[#7X4:2]-[#6X3:3]~[*:4]
t64	[*:1]-[#6X4:2]-[#7X3$(*~[#6X3,#6X2]):3]~[*:4]
t65	[*:1]-[#6X4:2]-[#7X3$(*~[#8X1]):3]~[#8X1:4]
t66	[#6X3:1]-[#7X3:2]-[#6X4:3]-[#6X3:4]
t67	[#6X4:1]-[#6X4:2]-[#7X3:3]-[#6X3:4]=[#8,#16,#7]
t68	[#8X2H0:1]-[#6X4:2]-[#7X3:3]-[#6X3:4]
t69	[#6X3:1]-[#7X3:2]-[#6X4;r3:3]-[#6X4;r3:4]
t70	[*:1]~[#7X2:2]-[#6X4:3]-[*:4]
t71	[#6X3:1]=[#7X2,#7X3+1:2]-[#6X4:3]-[#1:4]
t72	[#6X3:1]=[#7X2,#7X3+1:2]-[#6X4:3]-[#6X3,#6X4:4]
t73	[*:1]~[#7X3,#7X2-1:2]-[#6X3:3]~[*:4]
t74	[*:1]~[#7X3,#7X2-1:2]-!@[#6X3:3]~[*:4]
t75	[*:1]-[#7X3:2]-[#6X3$(*=[#8,#16,#7]):3]~[*:4]
t76	[#1:1]-[#7X3:2]-[#6X3:3]=[#8,#16,#7:4]
t77	[*:1]-[#7X3:2]-!@[#6X3:3](=[#8,#16,#7:4])-[#6,#1]
t78	[#1:1]-[#7X3:2]-!@[#6X3:3](=[#8,#16,#7:4])-[#6,#1]
t79	[*:1]-[#7X3:2]-!@[#6X3:3](=[#8,#16,#7:4])-[#7X3]
t80	[*:1]-[#7X3;r5:2]-@[#6X3;r5:3]~[*:4]
t81	[#8X1:1]~[#7X3:2]~[#6X3:3]~[*:4]
t82	[*:1]=[#7X2,#7X3+1:2]-[#6X3:3]-[*:4]
t83	[*:1]=[#7X2,#7X3+1:2]-[#6X3:3]=,:[*:4]
t84	[*:1]~[#7X2,#7X3$(*~[#8X1]):2]:[#6X3:3]~[*:4]
t85	[#6X3:1]:[#7X2:2]:[#6X3:3]:[#6X3:4]
t86	[*:1]-,:[#6X3:2]=[#7X2:3]-[*:4]
t87	[*:1]-[#7X3+1:2]=,:[#6X3:3]-,:[*:4]
t88	[#16X4,#16X3+0:1]-[#7X2:2]=[#6X3:3]-[#7X3:4]
t89	[#16X4,#16X3+0:1]-[#7X2:2]=[#6X3:3]-[#16X2,#16X3+1:4]
t90	[#7X2:1]~[#7X2:2]-[#6X3:3]~[#6X3:4]
t91	[#7X2:1]~[#7X2:2]-[#6X4:3]-[#6X3:4]

to be continued...

Table S3. Parameter ids and corresponding SMARTS patterns in the force field Sage 2.0.0. Bond parameters are labeled as ‘b#’, angle parameters as ‘a#’, proper torsion parameters as ‘t#’, and improper torsion parameters as ‘i#’. (continued)

… continued

Parameter ID	SMARTS Pattern
t92	[#7X2:1]~[#7X2:2]-[#6X4:3]~[#1:4]
t93	[*:1]-[#6X4:2]-[#8X2:3]-[#1:4]
t94	[#6X4:1]-[#6X4:2]-[#8X2H1:3]-[#1:4]
t95	[*:1]-[#6X4:2]-[#8X2H0:3]-[*:4]
t96	[#6X4:1]-[#6X4:2]-[#8X2H0:3]-[#6X4:4]
t97	[#6X4:1]-[#6X4:2]-[#8X2:3]-[#6X3:4]
t98	[#6X4:1]-[#8X2:2]-[#6X4:3]-[#8X2:4]
t99	[#6X4:1]-[#8X2:2]-[#6X4:3]-[#7X3:4]
t100	[#6X3:1]-[#8X2:2]-[#6X4;r3:3]-@[#6X4;r3:4]
t101	[#6X3:1]-[#8X2:2]-[#6X4;r3:3]-[#1:4]
t102	[#1:1]-[#8X2:2]-[#6X4;r3:3]-[#1:4]
t103	[#1:1]-[#8X2:2]-[#6X4;r3:3]-[#6X4:4]
t104	[#1:1]-[#8X2:2]-[#6X4;r3:3]-[#6X4;r3:4]
t105	[*:1]~[#6X3:2]-[#8X2:3]-[*:4]
t106	[*:1]~[#6X3:2]-[#8X2:3]-[#1:4]
t107	[*:1]~[#6X3:2](=[#8,#16,#7])-[#8X2H0:3]-[*:4]
t108	[*:1]~[#6X3:2](=[#8,#16,#7])-[#8:3]-[#1:4]
t109	[#1:1]-[#8X2:2]-[#6X3:3]=[#8X1:4]
t110	[#8,#16,#7:1]=[#6X3:2]-[#8X2H0:3]-[#6X4:4]
t111	[*:1]-[#8X2:2]@[#6X3:3]~[*:4]
t112	[*:1]-[#8X2+1:2]=[#6X3:3]-[*:4]
t113	[*:1]=[#8X2+1:2]-[#6:3]~[*:4]
t114	[*:1]~[#16:2]=,:[#6:3]~[*:4]
t115	[*:1]-[#16X2,#16X3+1:2]-[#6:3]~[*:4]
t116	[*:1]-[#16X2,#16X3+1:2]-[#6:3]-[#1:4]
t117	[#6X3:1]-@[#16X2,#16X1-1,#16X3+1:2]-@[#6X3,#7X2;r5:3]=@[#6,#7;r5:4]
t118	[*:1]~[#16X4,#16X3!+1:2]-[#6X4:3]-[*:4]
t119	[#6X4:1]-[#16X4,#16X3+0:2]-[#6X4:3]-[#1:4]
t120	[#6X4:1]-[#16X4,#16X3+0:2]-[#6X4:3]~[#6X4:4]
t121	[*:1]~[#16X4,#16X3+0:2]-[#6X3:3]~[*:4]
t122	[#6:1]-[#16X4,#16X3+0:2]-[#6X3:3]~[*:4]
t123	[*:1]~[#15:2]-[#6:3]-[*:4]
t124	[*:1]~[#15:2]-[#6X3:3]~[*:4]
t125	[*:1]-[#8:2]-[#8:3]-[*:4]
t126	[*:1]-[#8:2]-[#8H1:3]-[*:4]
t127	[*:1]~[#8X2:2]-[#7:3]~[*:4]
t128	[*:1]-[#8X2r5:2]-;@[#7X3r5:3]~[*:4]
t129	[*:1]-[#8X2r5:2]-;@[#7X2r5:3]~[*:4]
t130	[*:1]-[#7X4,#7X3:2]-[#7X4,#7X3:3]-[*:4]
t131	[#1:1]-[#7X4,#7X3:2]-[#7X4,#7X3:3]-[#1:4]
t132	[#6X4:1]-[#7X4,#7X3:2]-[#7X4,#7X3:3]-[#1:4]
t133	[#6X4:1]-[#7X4,#7X3:2]-[#7X4,#7X3:3]-[#6X4:4]
t134	[*:1]-[#7X4,#7X3:2]-[#7X3$(*~[#6X3,#6X2]):3]~[*:4]
t135	[*:1]-[#7X3$(*-[#6X3,#6X2]):2]-[#7X3$(*-[#6X3,#6X2]):3]-[*:4]

to be continued…

Table S3. Parameter ids and corresponding SMARTS patterns in the force field Sage 2.0.0. Bond parameters are labeled as 'b#', angle parameters as 'a#', proper torsion parameters as 't#', and improper torsion parameters as 'i#'. (continued)

… continued

Parameter ID	SMARTS Pattern
t136	[*:1]-[#7X3$(*-[#6X3,#6X2])r5:2]-@[#7X3$(*-[#6X3,#6X2])r5:3]~[*:4]
t137	[*:1]@[#7X2:2]@[#7X2:3]@[#7X2,#6X3:4]
t138	[*:1]~[#7X2:2]-[#7X3:3]~[*:4]
t139	[*:1]=[#7X2:2]-[#7X2:3]=[*:4]
t140	[*:1]~[#7X2:2]=,:[#7X2:3]~[*:4]
t141	[*:1]~[#7X3+1:2]=,:[#7X2:3]~[*:4]
t142	[*:1]-[#16X2,#16X3+1:2]-[!#6:3]~[*:4]
t143	[*:1]~[#16X4,#16X3+0:2]-[#7:3]~[*:4]
t144	[#6X4:1]-[#16X4,#16X3+0:2]-[#7X4,#7X3:3]-[#1:4]
t145	[#6X3:1]-[#16X4,#16X3+0:2]-[#7X4,#7X3:3]-[#1:4]
t146	[#6X4:1]-[#16X4,#16X3+0:2]-[#7X4,#7X3:3]-[#6X4:4]
t147	[#6X3:1]-[#16X4,#16X3+0:2]-[#7X4,#7X3:3]-[#6X4:4]
t148	[#8X1:1]~[#16X4,#16X3+0:2]-[#7X4,#7X3:3]-[#1:4]
t149	[#8X1:1]~[#16X4,#16X3+0:2]-[#7X4,#7X3:3]-[#6X4:4]
t150	[#6X3:1]-[#16X4,#16X3+0:2]-[#7X3:3]-[#6X3:4]
t151	[#6X4:1]-[#16X4,#16X3+0:2]-[#7X3:3]-[#6X3:4]
t152	[#8X1:1]~[#16X4,#16X3+0:2]-[#7X3:3]-[#6X3:4]
t153	[#8X1:1]~[#16X4,#16X3+0:2]-[#7X3:3]-[#7X2:4]
t154	[*:1]~[#16X4,#16X3+0:2]=,:[#7X2:3]-,:[*:4]
t155	[#6X4:1]-[#16X4,#16X3+0:2]-[#7X2:3]~[#6X3:4]
t156	[#8X1:1]~[#16X4,#16X3+0:2]-[#7X2:3]~[#6X3:4]
t157	[*:1]~[#16X4,#16X3+0:2]-[#8X2:3]-[*:4]
t158	[*:1]-[#16X2,#16X3+1:2]-[#16X2,#16X3+1:3]-[*:4]
t159	[*:1]-[#8X2:2]-[#15:3]~[*:4]
t160	[#8X2:1]-[#15:2]-[#8X2:3]-[#6X4:4]
t161	[*:1]~[#7X3:2]-[#15:3]~[*:4]
t162	[*:1]-[#7:2]-[#15:3]=[*:4]
t163	[#6X3:1]-[#7:2]-[#15:3]=[*:4]
t164	[*:1]~[#7:2]=[#15:3]~[*:4]
t165	[*:1]-[*:2]#[*:3]-[*:4]
t166	[*:1]~[*:2]-[*:3]#[*:4]
t167	[*:1]~[*:2]=[#6,#7,#16,#15;X2:3]=[*:4]
i1	[*:1]~[#6X3:2](~[*:3])~[*:4]
i2	[*:1]~[#6X3:2](~[#8X1:3])~[#8:4]
i3	[*:1]~[#7X3$(*~[#15,#16](!-[*])):2](~[*:3])~[*:4]
i4	[*:1]~[#7X3$(*~[#6X3]):2](~[*:3])~[*:4]
i5	[*:1]~[#7X3$(*~[#7X2]):2](~[*:3])~[*:4]
i6	[*:1]~[#7X3$(*@1-[*]=,:[*][*]=,:[*]@1):2](~[*:3])~[*:4]
i7	[*:1]~[#6X3:2](=[#7X2,#7X3+1:3])~[#7:4]

S1.9 Over-represented torsion parameters in TFD distribution tail

Table S4. Top ten torsion parameters most over-represented in outliers of TFD. Torsion parameters with wildcards on both ends that apply to a broader chemistry, and some hypervalent sulfur involved torsion parameters are common features in this list, which may need further investigation.

Parameter ID	SMIRKS Pattern
t25	`[#16X2,#16X1-1,#16X3+1:1]-[#6X3:2]-[#6X4:3]-[#7X4,#7X3:4]`
t49	`[*:1]~[#7a:2]:[#6a:3]~[*:4]`
t55	`[#1:1]-[#6X4:2]-[#7X3:3]-[#7X2:4]=[#7X2,#8X1]`
t88	`[#16X4,#16X3+0:1]-[#7X2:2]=[#6X3:3]-[#7X3:4]`
t91	`[#7X2:1]~[#7X2:2]-[#6X4:3]-[#6X3:4]`
t103	`[#1:1]-[#8X2:2]-[#6X4;r3:3]-[#6X4:4]`
t134	`[*:1]-[#7X4,#7X3:2]-[#7X3$(*~[#6X3,#6X2]):3]~[*:4]`
t135	`[*:1]-[#7X3$(*-[#6X3,#6X2]):2]-[#7X3$(*-[#6X3,#6X2]):3]-[*:4]`
t154	`[*:1]~[#16X4,#16X3+0:2]=,:[#7X2:3]-,:[*:4]`
t158	`[*:1]-[#16X2,#16X3+1:2]-[#16X2,#16X3+1:3]-[*:4]`

S1.10 Protein Ligand Binding Free Energy Benchmarking Data

Table S5. Summary and detailed data provenance for the protein-ligand benchmark set employed in in this paper. Each entry contains the target name/identifier, information about the crystal structures used, the source of the initial coordinates, ligand count, dynamic range and standard deviation of the activities, the number of perturbations, and the calculations performed. For each structure, the PDB ID is followed by the Iridium classification and Iridium score in the brackets. The Iridium classification categorizes each structure into not trustworthy (NT), medium trustworthy (MT) and highly trustworthy (HT) categories. The lower the Iridium score, the better the structure.[3] The diffraction-component precision indices (DPI) are also listed. For the activity data ("Ligand Information"), the number of ligands (count), the dynamic range ($\max(\Delta G) - \min(\Delta G)$) and the standard deviation of the ΔG ($\mathrm{std}(\Delta G)$) are given. The letter code in the columns “Input coordinates” and “Calculations” correspond to the following references: G1,[4] G2,[5] S,[6] P[7] and H marks this work. The force field label GAFF2.1x refers to the version GAFF2.1 for the protein-ligand systems marked with G1, while for the calculations marked with G2, GAFF2.11 version was used.

Target	Used structure		Input coordinates	Ligand Information			Edges	Calculations		
	PDB	DPI		Count	Dyn. Range [kcal mol^{-1}]	std(DG) [kcal mol^{-1}]	Count	OpenFF	GAFF2.1x	OPLS3e
BACE [8,9]	4DJW (HT, 0.32)	0.11	G1	36	3.9	0.8	58	H	G1	G1
BACE_HUNT [10–12]	4JPC (HT, 0.32)	0.12	G1	32	4.9	1.2	60	H	G1	G1
BACE_P2 [12,13]	3IN4 (HT, 0.59)	0.28	G1	12	0.9	0.3	26	H	G1	G1
CDK2 [9,14]	1H1Q (MT, 0.87)	0.28	G1	16	4.3	1.2	25	H	G1	G1
CDK8 [6,15]	5HNB (MT, 0.74)	0.22	S	33	5.7	1.3	54	H	G2	S
c-MET [6]	4R1Y (MT, 0.75)	0.17	S	24	6.2	1.7	57	H	G2	S
EG5 [6,16]	3L9H (MT, 0.88)	0.18	S	28	3.5	0.9	65	H	G2	S
Galectin [17,18]	5E89 (MT, 1.04)	0.07	G1	8	2.7	0.8	7	H	G1	G1
HIF2a [6,19]	5TBM (HT, 0.35)	0.17	S	42	4.6	1.1	92	H	G2	S
Jnk1 [9,20]	2GMX (NT,)	0.77	G1	21	3.4	0.8	31	H	G1	G1
MCL1 [9,21]	4HW3 (HT, 0.41)	0.26	G1	42	4.2	1.1	71	H	G1	G1
P38 [9,22]	3FLY (HT, 0.6)	0.12	G1	34	3.8	1.0	56	H	G1	G1
PDE2 [23,24]	6EZF (MT, 0.3)	0.07	G1	21	3.2	0.9	34	H	G1	G1
PDE10	- (- , -)	-	P	35			62	H	H	P
PFKFB3 [6,25]	6HVI (HT, 0.31)	0.11	S	40	3.7	1.1	66	H	G2	S
PTP1B [9,26]	2QBS (MT, 0.33)	0.15	G1	16	4.3	1.2	49	H	G1	G1
SHP2 [6,27]	5EHR (MT, 0.32)	0.1	S	26	4.3	1.2	56	H	G2	S
ROS1	-[a] (- , -)		P	28			63	H	H	P
SYK [6,28]	4PV0 (MT, 0.69)	0.19	S	44	6.0	1.0	101	H	G2	S
Thrombin [9,29]	2ZFF (HT, 0.3)	0.06	G1	11	1.7	0.5	16	H	G1	G1
TNKS2 [30]	4UI5 (HT, 0.29)	0.08	S	27	4.3	1.0	60	H	G2	S
TYK2 [9,31,32]	4GIH (HT, 0.5)	0.15	G1	16	4.3	1.3	24	H	G1	G1
all	(,)		-	599			1133			

[a] Structure not deposited in the PDB, but available in the protein ligand benchmark set[33]

Table S6. RMSE values against experimental protein ligand affinity benchmark set for OpenFF 1.0.0, OpenFF 2.0.0, GAFF 2.1x (specified for every system separately), and OPLS3e. The first four rows consider all targets aggregated, whereas the remaining rows consider the 22 targets individually. Next to the observed value, the median of the bootstrap distribution including the 2.5% and 97.5% percentiles are listed. They were computed by bootstrapping with replacement over molecules for 1000 iterations.

Target	Force Field	observed	estimated
all	OpenFF-1.0	1.25	1.25 [1.17,1.33]
all	OpenFF-2.0	1.29	1.29 [1.20,1.38]
all	OPLS3e	1.17	1.18 [1.09,1.27]
all	GAFF2.1x	1.22	1.22 [1.13,1.32]
bace	GAFF2.1	0.89	0.88 [0.71,1.06]
bace	OpenFF-2.0	0.77	0.77 [0.65,0.90]
bace	OPLS3e	1.08	1.07 [0.79,1.38]
bace	OpenFF-1.0	0.85	0.85 [0.66,1.03]
bace_hunt	OPLS3e	0.68	0.68 [0.52,0.82]
bace_hunt	OpenFF-1.0	0.78	0.76 [0.58,0.99]
bace_hunt	GAFF2.1	0.90	0.90 [0.71,1.10]
bace_hunt	OpenFF-2.0	0.84	0.83 [0.66,1.01]
bace_p2	OpenFF-2.0	0.72	0.72 [0.45,0.94]
bace_p2	OpenFF-1.0	0.80	0.79 [0.48,1.06]
bace_p2	OPLS3e	0.50	0.49 [0.35,0.62]
bace_p2	GAFF2.1	0.65	0.64 [0.27,0.93]
cdk2	OPLS3e	0.59	0.59 [0.41,0.75]
cdk2	GAFF2.1	0.67	0.66 [0.37,0.96]
cdk2	OpenFF-2.0	0.77	0.75 [0.50,1.03]
cdk2	OpenFF-1.0	0.68	0.67 [0.39,0.94]
cdk8	OpenFF-2.0	1.26	1.26 [1.06,1.47]
cdk8	OPLS3e	1.38	1.37 [1.00,1.74]
cdk8	GAFF2.11	0.83	0.83 [0.67,0.98]
cdk8	OpenFF-1.0	1.11	1.11 [0.86,1.34]
cmet	GAFF2.11	1.27	1.26 [1.01,1.54]
cmet	OpenFF-2.0	1.40	1.40 [1.05,1.75]
cmet	OPLS3e	0.91	0.91 [0.69,1.11]
cmet	OpenFF-1.0	1.02	1.01 [0.72,1.28]
eg5	OPLS3e	0.86	0.85 [0.57,1.15]
eg5	OpenFF-2.0	0.83	0.82 [0.64,0.98]
eg5	OpenFF-1.0	1.34	1.31 [0.84,1.84]
eg5	GAFF2.11	1.43	1.39 [0.96,2.02]
galectin	GAFF2.1	0.93	0.92 [0.69,1.20]
galectin	OPLS3e	0.27	0.27 [0.15,0.36]
galectin	OpenFF-1.0	0.85	0.84 [0.59,1.09]
galectin	OpenFF-2.0	0.57	0.56 [0.41,0.71]
hif2a	OPLS3e	1.10	1.07 [0.78,1.46]
hif2a	OpenFF-1.0	1.78	1.77 [1.44,2.12]
hif2a	GAFF2.11	1.53	1.53 [1.17,1.90]
hif2a	OpenFF-2.0	1.61	1.61 [1.27,1.95]
jnk1	GAFF2.1	0.97	0.95 [0.70,1.23]
jnk1	OpenFF-2.0	1.15	1.15 [0.98,1.31]

Continued on next page

Target	Force Field	observed	estimated
jnk1	OpenFF-1.0	0.86	0.85 [0.63,1.06]
jnk1	OPLS3e	0.63	0.62 [0.46,0.76]
mcl1	GAFF2.1	1.13	1.12 [0.86,1.36]
mcl1	OPLS3e	1.11	1.10 [0.93,1.29]
mcl1	OpenFF-2.0	1.03	1.02 [0.80,1.26]
mcl1	OpenFF-1.0	1.25	1.25 [1.03,1.46]
p38	GAFF2.1	0.77	0.76 [0.60,0.93]
p38	OpenFF-1.0	1.13	1.11 [0.79,1.48]
p38	OPLS3e	0.82	0.82 [0.62,1.02]
p38	OpenFF-2.0	0.82	0.81 [0.60,1.02]
pde10	OpenFF-1.0	1.70	1.71 [1.44,1.95]
pde10	GAFF2.11	1.62	1.63 [1.32,1.92]
pde10	OpenFF-2.0	2.63	2.62 [2.20,3.05]
pde10	OPLS3e	2.66	2.65 [2.29,2.97]
pde2	OpenFF-1.0	1.41	1.40 [1.07,1.70]
pde2	OpenFF-2.0	1.17	1.17 [0.96,1.37]
pde2	OPLS3e	1.16	1.15 [0.82,1.49]
pde2	GAFF2.1	0.96	0.94 [0.58,1.38]
pfkfb3	GAFF2.11	1.05	1.05 [0.84,1.25]
pfkfb3	OPLS3e	1.25	1.25 [1.05,1.45]
pfkfb3	OpenFF-2.0	1.09	1.09 [0.85,1.33]
pfkfb3	OpenFF-1.0	1.36	1.36 [1.15,1.56]
ptp1b	GAFF2.1	0.90	0.90 [0.73,1.07]
ptp1b	OpenFF-1.0	1.34	1.32 [0.79,1.84]
ptp1b	OPLS3e	0.73	0.73 [0.50,0.92]
ptp1b	OpenFF-2.0	1.30	1.26 [0.68,1.86]
ros1	GAFF2.11	1.17	1.15 [0.80,1.49]
ros1	OpenFF-1.0	1.42	1.42 [1.09,1.74]
ros1	OpenFF-2.0	1.26	1.24 [0.86,1.62]
ros1	OPLS3e	1.05	1.05 [0.80,1.29]
shp2	GAFF2.11	2.42	2.41 [1.69,3.07]
shp2	OpenFF-1.0	1.81	1.81 [1.32,2.28]
shp2	OPLS3e	0.96	0.95 [0.70,1.22]
shp2	OpenFF-2.0	1.75	1.74 [1.40,2.07]
syk	OPLS3e	1.16	1.15 [0.89,1.40]
syk	GAFF2.11	1.57	1.56 [1.27,1.85]
syk	OpenFF-2.0	1.41	1.39 [1.07,1.69]
syk	OpenFF-1.0	1.07	1.06 [0.86,1.26]
thrombin	OpenFF-2.0	0.75	0.73 [0.48,0.96]
thrombin	GAFF2.1	0.75	0.75 [0.52,0.96]
thrombin	OpenFF-1.0	0.81	0.80 [0.54,1.01]
thrombin	OPLS3e	0.77	0.77 [0.49,1.03]
tnks2	GAFF2.11	0.89	0.88 [0.70,1.08]
tnks2	OPLS3e	1.43	1.43 [1.11,1.72]
tnks2	OpenFF-2.0	1.05	1.04 [0.81,1.26]
tnks2	OpenFF-1.0	1.04	1.04 [0.79,1.26]
tyk2	OPLS3e	0.50	0.48 [0.27,0.71]

Continued on next page

Target	Force Field	observed	estimated
tyk2	GAFF2.1	0.97	0.97 [0.76,1.17]
tyk2	OpenFF-2.0	0.71	0.70 [0.48,0.91]
tyk2	OpenFF-1.0	0.76	0.74 [0.49,0.99]

Table S7. Kendall's τ values against experimental protein ligand affinity benchmark set for OpenFF 1.0.0, OpenFF 2.0.0, GAFF 2.1x (specified for every system separately), and OPLS3e. The first four rows aggregate all targets, while the remaining rows consider the 22 targets individually. Next to the observed value, the median of the bootstrap distribution including the 2.5% and 97.5% percentiles are listed. They were computed by bootstrapping with replacement over molecules for 1000 iterations.

Target	Force Field	observed	estimated
all	OpenFF-1.0	0.39	0.39 [0.14,0.60]
all	OpenFF-2.0	0.44	0.45 [0.21,0.64]
all	OPLS3e	0.53	0.53 [0.31,0.71]
all	GAFF2.1x	0.43	0.43 [0.18,0.64]
bace	OpenFF-1.0	0.44	0.43 [0.22,0.65]
bace	OPLS3e	0.45	0.44 [0.21,0.64]
bace	GAFF2.1	0.28	0.29 [0.06,0.47]
bace	OpenFF-2.0	0.52	0.52 [0.36,0.67]
bace_hunt	OPLS3e	0.71	0.71 [0.57,0.83]
bace_hunt	OpenFF-1.0	0.59	0.60 [0.35,0.77]
bace_hunt	OpenFF-2.0	0.58	0.58 [0.39,0.74]
bace_hunt	GAFF2.1	0.51	0.52 [0.29,0.70]
bace_p2	OpenFF-2.0	0.27	0.28 [-0.17,0.63]
bace_p2	OpenFF-1.0	0.27	0.29 [-0.15,0.62]
bace_p2	OPLS3e	0.46	0.49 [-0.02,0.87]
bace_p2	GAFF2.1	0.43	0.44 [-0.14,0.89]
cdk2	GAFF2.1	0.59	0.60 [0.27,0.84]
cdk2	OpenFF-2.0	0.56	0.58 [0.27,0.80]
cdk2	OPLS3e	0.61	0.62 [0.33,0.85]
cdk2	OpenFF-1.0	0.66	0.67 [0.33,0.89]
cdk8	OpenFF-2.0	0.60	0.60 [0.45,0.73]
cdk8	OPLS3e	0.56	0.56 [0.40,0.73]
cdk8	OpenFF-1.0	0.66	0.66 [0.52,0.78]
cdk8	GAFF2.11	0.70	0.70 [0.53,0.82]
cmet	GAFF2.11	0.78	0.78 [0.60,0.90]
cmet	OPLS3e	0.77	0.78 [0.61,0.89]
cmet	OpenFF-1.0	0.70	0.69 [0.53,0.84]
cmet	OpenFF-2.0	0.70	0.71 [0.47,0.86]
eg5	OPLS3e	0.57	0.58 [0.38,0.75]
eg5	OpenFF-2.0	0.41	0.42 [0.21,0.60]
eg5	OpenFF-1.0	0.31	0.31 [0.07,0.54]
eg5	GAFF2.11	0.38	0.38 [0.15,0.58]
galectin	OpenFF-2.0	0.47	0.51 [-0.13,0.92]
galectin	OPLS3e	0.76	0.81 [0.25,1.00]
galectin	GAFF2.1	0.18	0.19 [-0.43,0.68]
galectin	OpenFF-1.0	0.18	0.22 [-0.42,0.67]

Continued on next page

Target	Force Field	observed	estimated
hif2a	OPLS3e	0.47	0.48 [0.26,0.64]
hif2a	GAFF2.11	0.33	0.33 [0.13,0.51]
hif2a	OpenFF-1.0	0.25	0.26 [0.06,0.44]
hif2a	OpenFF-2.0	0.35	0.35 [0.15,0.54]
jnk1	GAFF2.1	0.65	0.66 [0.42,0.85]
jnk1	OpenFF-2.0	0.71	0.71 [0.53,0.87]
jnk1	OpenFF-1.0	0.54	0.55 [0.23,0.78]
jnk1	OPLS3e	0.74	0.75 [0.57,0.89]
mcl1	OPLS3e	0.43	0.43 [0.26,0.58]
mcl1	OpenFF-2.0	0.54	0.54 [0.37,0.68]
mcl1	GAFF2.1	0.61	0.61 [0.45,0.73]
mcl1	OpenFF-1.0	0.42	0.43 [0.25,0.58]
p38	OPLS3e	0.66	0.66 [0.50,0.81]
p38	GAFF2.1	0.50	0.50 [0.33,0.66]
p38	OpenFF-2.0	0.50	0.50 [0.30,0.67]
p38	OpenFF-1.0	0.60	0.60 [0.43,0.75]
pde10	OpenFF-1.0	0.27	0.27 [0.02,0.51]
pde10	GAFF2.11	0.28	0.28 [0.00,0.51]
pde10	OpenFF-2.0	0.21	0.23 [-0.03,0.46]
pde10	OPLS3e	0.41	0.42 [0.19,0.63]
pde2	GAFF2.1	0.26	0.26 [-0.15,0.59]
pde2	OPLS3e	0.19	0.19 [-0.13,0.50]
pde2	OpenFF-1.0	0.01	0.00 [-0.32,0.36]
pde2	OpenFF-2.0	-0.08	-0.09 [-0.36,0.23]
pfkfb3	GAFF2.11	0.43	0.43 [0.25,0.59]
pfkfb3	OpenFF-2.0	0.52	0.52 [0.36,0.65]
pfkfb3	OPLS3e	0.60	0.60 [0.48,0.72]
pfkfb3	OpenFF-1.0	0.32	0.32 [0.13,0.47]
ptp1b	GAFF2.1	0.37	0.37 [0.01,0.67]
ptp1b	OpenFF-1.0	0.38	0.39 [0.03,0.65]
ptp1b	OpenFF-2.0	0.42	0.44 [0.10,0.69]
ptp1b	OPLS3e	0.73	0.74 [0.57,0.88]
ros1	OpenFF-1.0	0.23	0.22 [-0.06,0.51]
ros1	GAFF2.11	0.42	0.42 [0.18,0.63]
ros1	OPLS3e	0.47	0.48 [0.26,0.65]
ros1	OpenFF-2.0	0.42	0.43 [0.17,0.65]
shp2	GAFF2.11	0.21	0.21 [-0.11,0.49]
shp2	OPLS3e	0.56	0.57 [0.40,0.71]
shp2	OpenFF-1.0	0.14	0.14 [-0.20,0.44]
shp2	OpenFF-2.0	0.14	0.16 [-0.21,0.48]
syk	GAFF2.11	0.30	0.30 [0.09,0.47]
syk	OPLS3e	0.28	0.28 [0.06,0.49]
syk	OpenFF-2.0	0.40	0.39 [0.21,0.57]
syk	OpenFF-1.0	0.27	0.28 [0.09,0.45]
thrombin	OpenFF-2.0	0.42	0.42 [-0.06,0.80]
thrombin	GAFF2.1	0.24	0.23 [-0.14,0.70]
thrombin	OpenFF-1.0	0.20	0.22 [-0.20,0.57]

Continued on next page

Target	Force Field	observed	estimated
thrombin	OPLS3e	0.60	0.62 [0.22,0.95]
tnks2	OpenFF-2.0	0.31	0.31 [0.02,0.54]
tnks2	GAFF2.11	0.46	0.47 [0.21,0.66]
tnks2	OpenFF-1.0	0.38	0.39 [0.12,0.59]
tnks2	OPLS3e	0.30	0.30 [-0.01,0.54]
tyk2	GAFF2.1	0.40	0.41 [-0.05,0.71]
tyk2	OpenFF-2.0	0.67	0.67 [0.39,0.88]
tyk2	OpenFF-1.0	0.57	0.59 [0.20,0.82]
tyk2	OPLS3e	0.73	0.75 [0.45,0.96]

S1.11 Distribution of force constants in the force fields

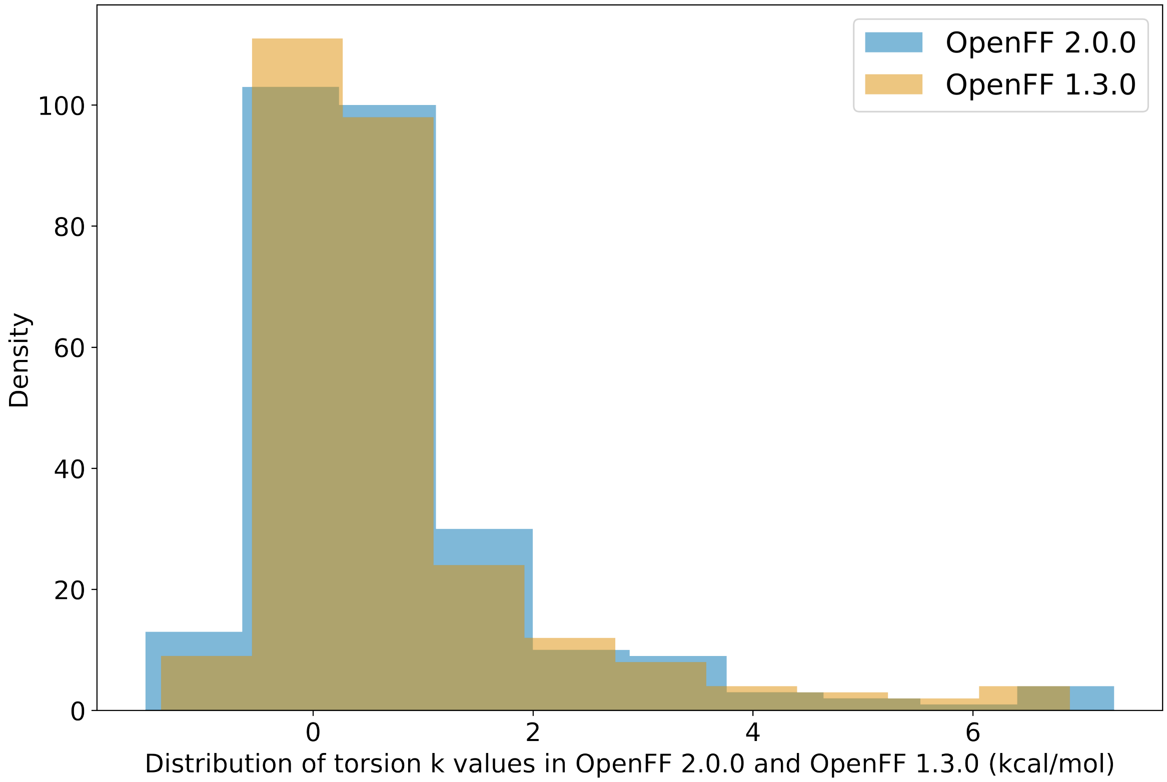

Figure S6. The plot shows the distributions of torsion force constants in OpenFF 1.3.0, the starting point for fitting, and in OpenFF-2.0.0. There was a slight adjustment in the force constant values but both the distributions overlap well. As expected most of the k values were near zero.

S1.12 Benchmark with RMSD matched conformers

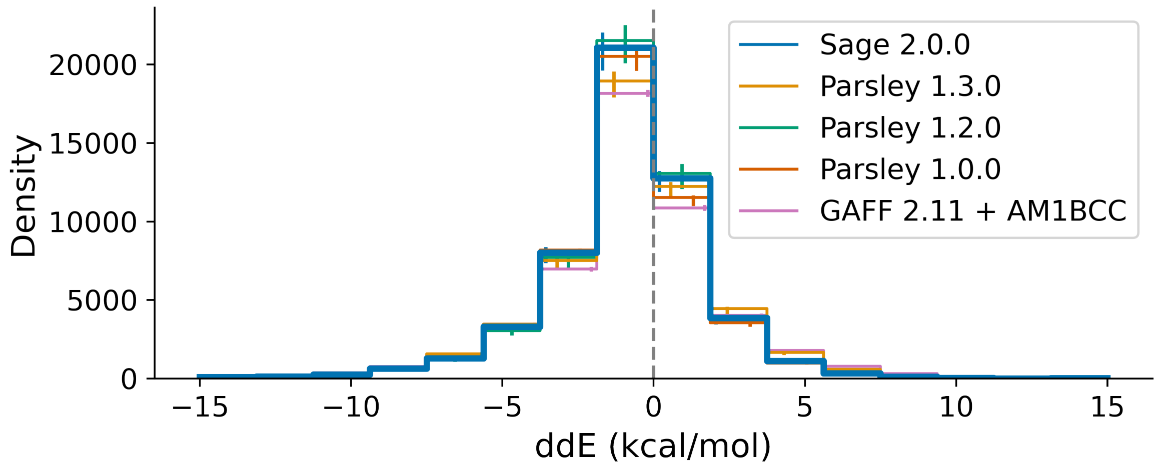

Figure S7. Step plot showing $\Delta\Delta E$ with respect to all generations of force fields. The energy comparisons here were done on MM conformers that match to any QM conformer within 1 Angstrom, and if the MM conformations did not match any QM conformer, they were excluded. The error bars were bootstrapped errors for each bin. The force field Sage 2.0.0 was highlighted with a bold line, while other force fields were shown with narrower lines. The same trend was observed as in Figure 7 (of main text) , which performs the direct comparison of conformers as described in the text

References

[1] **Haider N**. Functionality Pattern Matching as an Efficient Complementary Structure/Reaction Search Tool: an Open-Source Approach. Molecules. 2010; 15(8):5079–5092. doi: 10.3390/molecules15085079. (Cited on S5)

[2] **Boothroyd S**, Non-Bonded Optimization and Assessment; 2022. https://github.com/SimonBoothroyd/nonbonded. (Cited on S5)

[3] **Warren GL**, Do TD, Kelley BP, Nicholls A, Warren SD. Essential Considerations for Using Protein–Ligand Structures in Drug Discovery. Drug Discovery Today. 2012; 17(23-24):1270–1281. doi: 10.1016/j.drudis.2012.06.011. (Cited on S15)

[4] **Gapsys V**, Pérez-Benito L, Aldeghi M, Seeliger D, van Vlijmen H, Tresadern G, de Groot BL. Large scale relative protein ligand binding affinities using non-equilibrium alchemy. Chemical Science. 2020; 11(4):1140–1152. doi: 10.1039/C9SC03754C. (Cited on S15)

[5] **Gapsys V**, Hahn DF, Tresadern G, Mobley DL, Rampp M, de Groot BL. Pre-Exascale Computing of Protein–Ligand Binding Free Energies with Open Source Software for Drug Design. Journal of chemical information and modeling. 2022; 62(5):1172–1177. (Cited on S15)

[6] **Schindler CEM**, Baumann H, Blum A, Böse D, Buchstaller HP, Burgdorf L, Cappel D, Chekler E, Czodrowski P, Dorsch D, Eguida MKI, Follows B, Fuchß T, Grädler U, Gunera J, Johnson T, Jorand Lebrun C, Karra S, Klein M, Knehans T, et al. Large-Scale Assessment of Binding Free Energy Calculations in Active Drug Discovery Projects. Journal of Chemical Information and Modeling. 2020; 60(11):5457–5474. doi: 10.1021/acs.jcim.0c00900. (Cited on S15)

[7] **Pérez-Benito L**, Casajuana-Martin N, Jiménez-Rosés M, Van Vlijmen H, Tresadern G. Predicting activity cliffs with free-energy perturbation. Journal of Chemical Theory and Computation. 2019; 15(3):1884–1895. (Cited on S15)

[8] **Cumming JN**, Smith EM, Wang L, Misiaszek J, Durkin J, Pan J, Iserloh U, Wu Y, Zhu Z, Strickland C, Voigt J, Chen X, Kennedy ME, Kuvelkar R, Hyde LA, Cox K, Favreau L, Czarniecki MF, Greenlee WJ, McKittrick BA, et al. Structure Based Design of Iminohydantoin BACE1 Inhibitors: Identification of an Orally Available, Centrally Active BACE1 Inhibitor. Bioorganic & Medicinal Chemistry Letters. 2012; 22(7):2444–2449. doi: 10.1016/j.bmcl.2012.02.013. (Cited on S15)

[9] **Wang L**, Wu Y, Deng Y, Kim B, Pierce L, Krilov G, Lupyan D, Robinson S, Dahlgren MK, Greenwood J, Romero DL, Masse C, Knight JL, Steinbrecher T, Beuming T, Damm W, Harder E, Sherman W, Brewer M, Wester R, et al. Accurate and Reliable Prediction of Relative Ligand Binding Potency in Prospective Drug Discovery by Way of a Modern Free-Energy Calculation Protocol and Force Field. Journal of the American Chemical Society. 2015; 137(7):2695–2703. doi: 10.1021/ja512751q. (Cited on S15)

[10] **Hunt KW**, Cook AW, Watts RJ, Clark CT, Vigers G, Smith D, Metcalf AT, Gunawardana IW, Burkard M, Cox AA, Geck Do MK, Dutcher D, Thomas AA, Rana S, Kallan NC, DeLisle RK, Rizzi JP, Regal K, Sammond D, Groneberg R, et al. Spirocyclic -Site Amyloid Precursor Protein Cleaving Enzyme 1 (BACE1) Inhibitors: From Hit to Lowering of Cerebrospinal Fluid (CSF) Amyloid in a Higher Species. Journal of Medicinal Chemistry. 2013; 56(8):3379–3403. doi: 10.1021/jm4002154. (Cited on S15)

[11] **Ciordia M**, Pérez-Benito L, Delgado F, Trabanco AA, Tresadern G. Application of Free Energy Perturbation for the Design of BACE1 Inhibitors. Journal of Chemical Information and Modeling. 2016; 56(9):1856–1871. doi: 10.1021/acs.jcim.6b00220.

[12] **Keränen H**, Pérez-Benito L, Ciordia M, Delgado F, Steinbrecher TB, Oehlrich D, van Vlijmen HWT, Trabanco AA, Tresadern G. Acylguanidine Beta Secretase 1 Inhibitors: A Combined Experimental and Free Energy Perturbation Study. Journal of Chemical Theory and Computation. 2017; 13(3):1439–1453. doi: 10.1021/acs.jctc.6b01141. (Cited on S15)

[13] **Malamas MS**, Erdei J, Gunawan I, Turner J, Hu Y, Wagner E, Fan K, Chopra R, Olland A, Bard J, Jacobsen S, Magolda RL, Pangalos M, Robichaud AJ. Design and Synthesis of 5,5′-Disubstituted Aminohydantoins as Potent and Selective Human β-Secretase (BACE1) Inhibitors. Journal of Medicinal Chemistry. 2010; 53(3):1146–1158. doi: 10.1021/jm901414e. (Cited on S15)

[14] **Hardcastle IR**, Arris CE, Bentley J, Boyle FT, Chen Y, Curtin NJ, Endicott JA, Gibson AE, Golding BT, Griffin RJ, Jewsbury P, Menyerol J, Mesguiche V, Newell DR, Noble MEM, Pratt DJ, Wang LZ, Whitfield HJ. N ^{2} -Substituted O ^{6} -Cyclohexylmethylguanine Derivatives: Potent Inhibitors of Cyclin-Dependent Kinases 1 and 2. Journal of Medicinal Chemistry. 2004; 47(15):3710–3722. doi: 10.1021/jm0311442. (Cited on S15)

[15] **Schiemann K**, Mallinger A, Wienke D, Esdar C, Poeschke O, Busch M, Rohdich F, Eccles SA, Schneider R, Raynaud FI, Czodrowski P, Musil D, Schwarz D, Urbahns K, Blagg J. Discovery of Potent and Selective CDK8 Inhibitors from an HSP90 Pharmacophore. Bioorganic & Medicinal Chemistry Letters. 2016; 26(5):1443–1451. doi: 10.1016/j.bmcl.2016.01.062. (Cited on S15)

[16] **Schiemann K**, Finsinger D, Zenke F, Amendt C, Knöchel T, Bruge D, Buchstaller HP, Emde U, Stähle W, Anzali S. The Discovery and Optimization of Hexahydro-2H-Pyrano[3,2-c]Quinolines (HHPQs) as Potent and Selective Inhibitors of the Mitotic Kinesin-5. Bioorganic & Medicinal Chemistry Letters. 2010; 20(5):1491–1495. doi: 10.1016/j.bmcl.2010.01.110. (Cited on S15)

[17] **Delaine T**, Collins P, MacKinnon A, Sharma G, Stegmayr J, Rajput VK, Mandal S, Cumpstey I, Larumbe A, Salameh BA, Kahl-Knutsson B, van Hattum H, van Scherpenzeel M, Pieters RJ, Sethi T, Schambye H, Oredsson S, Leffler H, Blanchard H, Nilsson UJ. Galectin-3-Binding Glycomimetics That Strongly Reduce Bleomycin-Induced Lung Fibrosis and Modulate Intracellular Glycan Recognition. ChemBioChem. 2016; 17(18):1759–1770. doi: 10.1002/cbic.201600285. (Cited on S15)

[18] **Manzoni F**, Ryde U. Assessing the Stability of Free-Energy Perturbation Calculations by Performing Variations in the Method. Journal of Computer-Aided Molecular Design. 2018; 32(4):529–536. doi: 10.1007/s10822-018-0110-5. (Cited on S15)

[19] **Wallace EM**, Rizzi JP, Han G, Wehn PM, Cao Z, Du X, Cheng T, Czerwinski RM, Dixon DD, Goggin BS, Grina JA, Halfmann MM, Maddie MA, Olive SR, Schlachter ST, Tan H, Wang B, Wang K, Xie S, Xu R, et al. A Small-Molecule Antagonist of HIF2α Is Efficacious in Preclinical Models of Renal Cell Carcinoma. Cancer Research. 2016; 76(18):5491–5500. doi: 10.1158/0008-5472.CAN-16-0473. (Cited on S15)

[20] **Szczepankiewicz BG**, Kosogof C, Nelson LTJ, Liu G, Liu B, Zhao H, Serby MD, Xin Z, Liu M, Gum RJ, Haasch DL, Wang S, Clampit JE, Johnson EF, Lubben TH, Stashko MA, Olejniczak ET, Sun C, Dorwin SA, Haskins K, et al. Aminopyridine-Based c-Jun N-Terminal Kinase Inhibitors with Cellular Activity and Minimal Cross-Kinase Activity †. Journal of Medicinal Chemistry. 2006; 49(12):3563–3580. doi: 10.1021/jm060199b. (Cited on S15)

[21] **Friberg A**, Vigil D, Zhao B, Daniels RN, Burke JP, Garcia-Barrantes PM, Camper D, Chauder BA, Lee T, Olejniczak ET, Fesik SW. Discovery of Potent Myeloid Cell Leukemia 1 (Mcl-1) Inhibitors Using Fragment-Based Methods and Structure-Based Design. Journal of Medicinal Chemistry. 2013; 56(1):15–30. doi: 10.1021/jm301448p. (Cited on S15)

[22] **Goldstein DM**, Soth M, Gabriel T, Dewdney N, Kuglstatter A, Arzeno H, Chen J, Bingenheimer W, Dalrymple SA, Dunn J, Farrell R, Frauchiger S, La Fargue J, Ghate M, Graves B, Hill RJ, Li F, Litman R, Loe B, McIntosh J, et al. Discovery of 6-(2,4-Difluorophenoxy)-2-[3-Hydroxy-1-(2-Hydroxyethyl)Propylamino]-8-Methyl-8 H -Pyrido[2,3- d]Pyrimidin-7-One (Pamapimod) and 6-(2,4-Difluorophenoxy)-8-Methyl-2-(Tetrahydro-2 H -Pyran-4-Ylamino)Pyrido[2,3- d]Pyrimidin-7(8 H)-One (R1487) as Orally Bioavailable and Highly Selective Inhibitors of P38α Mitogen-Activated Protein Kinase. Journal of Medicinal Chemistry. 2011; 54(7):2255–2265. doi: 10.1021/jm101423y. (Cited on S15)

[23] **Buijnsters P**, De Angelis M, Langlois X, Rombouts FJR, Sanderson W, Tresadern G, Ritchie A, Trabanco AA, Van-Hoof G, Roosbroeck YV, Andrés JI. Structure-Based Design of a Potent, Selective, and Brain Penetrating PDE2 Inhibitor with Demonstrated Target Engagement. ACS Medicinal Chemistry Letters. 2014; 5(9):1049–1053. doi: 10.1021/ml500262u. (Cited on S15)

[24] **Pérez-Benito L**, Keränen H, van Vlijmen H, Tresadern G. Predicting Binding Free Energies of PDE2 Inhibitors. The Difficulties of Protein Conformation. Scientific Reports. 2018; 8(1):4883. doi: 10.1038/s41598-018-23039-5. (Cited on S15)

[25] **Boutard N**, Białas A, Sabiniarz A, Guzik P, Banaszak K, Biela A, Bień M, Buda A, Bugaj B, Cieluch E, Cierpich A, Dudek Ł, Eggenweiler HM, Fogt J, Gaik M, Gondela A, Jakubiec K, Jurzak M, Kitlińska A, Kowalczyk P, et al. Discovery and Structure-Activity Relationships of N -Aryl 6-Aminoquinoxalines as Potent PFKFB3 Kinase Inhibitors. ChemMedChem. 2019; 14(1):169–181. doi: 10.1002/cmdc.201800569. (Cited on S15)

[26] **Wilson DP**, Wan ZK, Xu WX, Kirincich SJ, Follows BC, Joseph-McCarthy D, Foreman K, Moretto A, Wu J, Zhu M, Binnun E, Zhang YL, Tam M, Erbe DV, Tobin J, Xu X, Leung L, Shilling A, Tam SY, Mansour TS, et al. Structure-Based Optimization of Protein Tyrosine Phosphatase 1B Inhibitors: From the Active Site to the Second Phosphotyrosine Binding Site. Journal of Medicinal Chemistry. 2007; 50(19):4681–4698. doi: 10.1021/jm0702478. (Cited on S15)

[27] **Chen YNP**, LaMarche MJ, Chan HM, Fekkes P, Garcia-Fortanet J, Acker MG, Antonakos B, Chen CHT, Chen Z, Cooke VG, Dobson JR, Deng Z, Fei F, Firestone B, Fodor M, Fridrich C, Gao H, Grunenfelder D, Hao HX, Jacob J, et al. Allosteric Inhibition of SHP2 Phosphatase Inhibits Cancers Driven by Receptor Tyrosine Kinases. Nature. 2016; 535(7610):148–152. doi: 10.1038/nature18621. (Cited on S15)

[28] **Currie KS**, Kropf JE, Lee T, Blomgren P, Xu J, Zhao Z, Gallion S, Whitney JA, Maclin D, Lansdon EB, Maciejewski P, Rossi AM, Rong H, Macaluso J, Barbosa J, Di Paolo JA, Mitchell SA. Discovery of GS-9973, a Selective and Orally Efficacious Inhibitor of Spleen Tyrosine Kinase. Journal of Medicinal Chemistry. 2014; 57(9):3856–3873. doi: 10.1021/jm500228a. (Cited on S15)

[29] **Baum B**, Mohamed M, Zayed M, Gerlach C, Heine A, Hangauer D, Klebe G. More than a Simple Lipophilic Contact: A Detailed Thermodynamic Analysis of Nonbasic Residues in the S1 Pocket of Thrombin. Journal of Molecular Biology. 2009; 390(1):56–69. doi: 10.1016/j.jmb.2009.04.051. (Cited on S15)

[30] **Buchstaller HP**, Anlauf U, Dorsch D, Kuhn D, Lehmann M, Leuthner B, Musil D, Radtki D, Ritzert C, Rohdich F, Schneider R, Esdar C. Discovery and Optimization of 2-Arylquinazolin-4-Ones into a Potent and Selective Tankyrase Inhibitor Modulating Wnt Pathway Activity. Journal of Medicinal Chemistry. 2019; 62(17):7897–7909. doi: 10.1021/acs.jmedchem.9b00656. (Cited on S15)

[31] **Liang J**, Tsui V, Van Abbema A, Bao L, Barrett K, Beresini M, Berezhkovskiy L, Blair WS, Chang C, Driscoll J, Eigenbrot C, Ghilardi N, Gibbons P, Halladay J, Johnson A, Kohli PB, Lai Y, Liimatta M, Mantik P, Menghrajani K, et al. Lead Identification of Novel and Selective TYK2 Inhibitors. European Journal of Medicinal Chemistry. 2013; 67:175–187. doi: 10.1016/j.ejmech.2013.03.070. (Cited on S15)

[32] **Liang J**, van Abbema A, Balazs M, Barrett K, Berezhkovsky L, Blair W, Chang C, Delarosa D, DeVoss J, Driscoll J, Eigenbrot C, Ghilardi N, Gibbons P, Halladay J, Johnson A, Kohli PB, Lai Y, Liu Y, Lyssikatos J, Mantik P, et al. Lead Optimization of a 4-Aminopyridine Benzamide Scaffold To Identify Potent, Selective, and Orally Bioavailable TYK2 Inhibitors. Journal of Medicinal Chemistry. 2013; 56(11):4521–4536. doi: 10.1021/jm400266t. (Cited on S15)

[33] **Hahn DF**, Wagner J, openforcefield/protein-ligand-benchmark: 0.2.0 Addition of New Targets; 2021. doi: 10.5281/ZENODO.5679599. (Cited on S15)
